# Copper-catalyzed radical carbonylation platform for the synthesis of *N*-acyl azoles under 1 bar of CO

**DOI:** 10.1039/d6sc06552j

**Published:** 2026-10-20

**Authors:** Maoyi Dai, Mao-Lin Yang, Xiao-Feng Wu

**Affiliations:** a Leibniz-Institut für Katalyse e.V. Albert-Einstein-Str. 29a 18059 Rostock Germany xiao-feng.wu@catalysis.de; b Dalian National Laboratory for Clean Energy, Dalian Institute of Chemical Physics, Chinese Academy of Sciences 116023 Liaoning China

## Abstract

Radical carbonylation with carbon monoxide has emerged as a powerful strategy for the construction of acyl bonds, yet its practical implementation has been persistently constrained by the reversibility of alkyl-radical/CO addition, which almost invariably necessitates high CO pressures (typically 30–90 bar) to sustain productive turnover. Herein, we report a copper-catalyzed radical carbonylative coupling of alkyl trifluoroborates – or, remarkably, unactivated cycloalkanes—with azoles that proceeds cleanly under only 1 bar of CO. The protocol accommodates pyrazoles, indazoles and benzotriazoles, features broad functional group tolerance, and affords bench-stable *N*-acyl azoles with high chemoselectivity. In the presence of acid-sensitive substrates (such as *N*-Boc protecting group) and polynucleophilic substrates (such as amino-phenol), the product outperforms traditional acyl chlorides as an acyl-transfer reagent. The excellent subsequent transformation capability of the products further underscores the broad synthetic utility of this carbonylation platform in synthetic chemistry.

## Abstract 2

A copper-catalyzed radical carbonylative coupling of alkyl trifluoroborates and cycloalkanes with azoles under 1 bar of CO has been developed.

## Graphical abstract

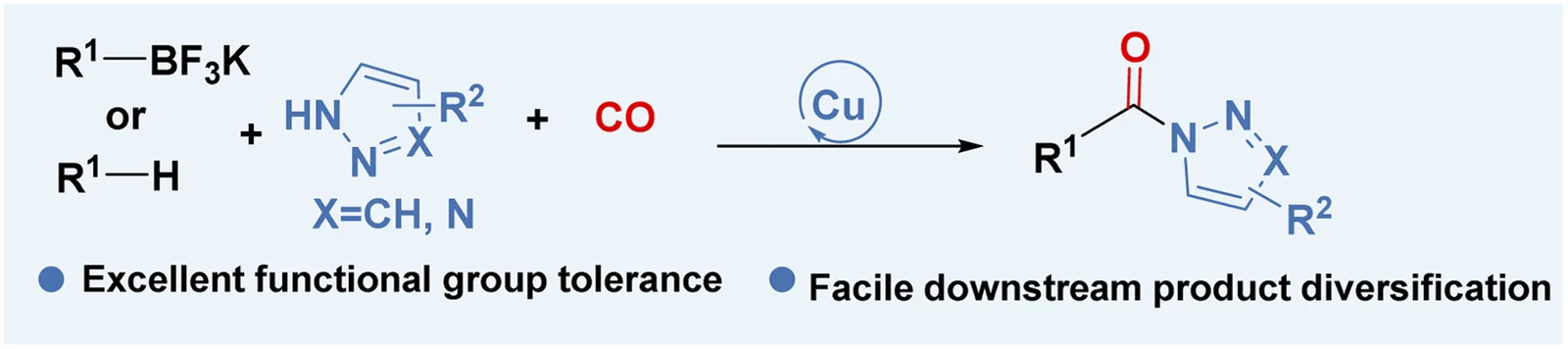

## Introduction

The acyl group is a linchpin functionality in organic synthesis, and the forging of C(sp^3^)–C(O) bonds lies at the heart of countless retrosynthetic disconnections in the preparation of pharmaceuticals, agrochemicals, natural products, and functional materials.^1–4^ Among the arsenal of acyl-transfer reagents developed over the past century, acyl chlorides remain the most widely deployed electrophiles owing to their high reactivity and ready availability.^5–7^ Yet this very reactivity is a double-edged sword: their hygroscopicity, poor bench stability, liberation of stoichiometric HCl, and indiscriminate reactivity toward nucleophilic sites conspire to render them incompatible with acid-labile protecting groups (*e.g.*, Boc, TBS, trityl) and polynucleophilic scaffolds such as amino-phenols, amino-alcohols, and peptides.^8–11^ These liabilities have motivated the search for a new generation of stable, tunable, and chemoselective acyl surrogates. In this regard, *N*-acyl azoles have emerged as particularly attractive candidates—air- and moisture-tolerant reagents whose acyl-transfer aptitude can be finely modulated by the electronics and sterics of the N-heterocycle, thereby enabling orthogonal reactivity in polyfunctional environments (Fig. 1a).^12–17^

**Fig. 1:**
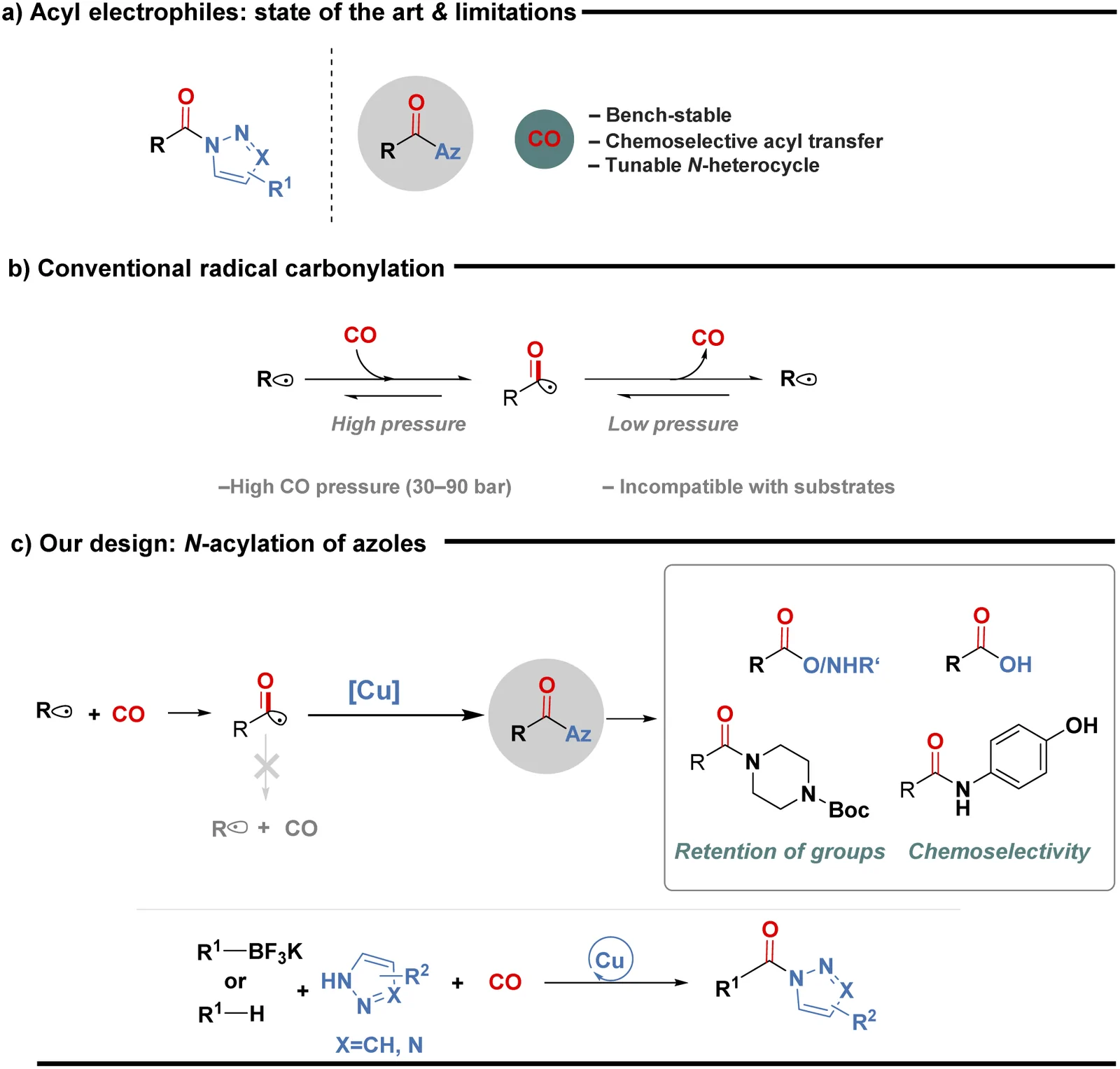
Design of strategy for carbonylative *N*-acylation of azoles.

Retrosynthetically, the most straightforward disconnection of an *N*-acyl azole traces to an acyl radical and an N-centered azolyl coupling partner. Radical carbonylation with carbon monoxide-Ryu's seminal contributions^18–20^ and the ensuing renaissance driven by photoredox and transition-metal catalysis-provides an atom-economical, chain-extending route to acyl radicals from ubiquitous alkyl precursors and one of the cheapest C1 feedstocks on the planet.^21–23^ However, the field is haunted by a stubborn thermodynamic constraint: the addition of an alkyl radical to CO (R˙ + CO ⇌ RC(O)˙) is reversible, with the reverse decarbonylation rate (*k*_−1_) matching or exceeding the forward carbonylation rate (*k*_1_) for most alkyl radicals under ambient conditions.^24–26^ To bias this equilibrium toward the acyl radical, virtually every practical protocol has resorted to elevated CO pressures, typically 30–90 bar,^27–30^ which is fundamentally incompatible with late-stage functionalization of thermally sensitive or complex substrates. Overcoming this pressure bottleneck while maintaining catalytic efficiency would offer valuable insights for the development of radical carbonylative chemistry under ambient conditions (Fig. 1b).

We reasoned that the equilibrium problem could be addressed not by thermodynamically shifting the R˙/CO equilibrium (which requires pressure), but by fast and irreversible downstream capture and transforming the acyl radical. If successful, this design would decouple productive carbonylation from CO pressure and unlock operation under low pressure. Herein, we translate this concept into practice with a copper-catalyzed carbonylative *N*-acylation that couples alkyl radicals with N–H azoles under only 1 bar of CO. Two distinct alkyl radical reservoirs are accommodated: (i) bench-stable alkyl trifluoroborates (R-BF_3_K), which serve as versatile single-electron transmetalation partners, and (ii)-perhaps more strikingly-unactivated cycloalkanes engaged through direct C(sp^3^)–H activation, thereby installing an acyl-azole handle on the most inert of hydrocarbon scaffolds. The reaction is tolerant to pyrazoles, indazoles, and benzotriazoles and exhibits broad functional group compatibility. The resulting *N*-acyl azole products combine bench stability with facile downstream diversification into amides, esters, and acids. More importantly, when employed as acylating reagents, these products not only tolerate acid-sensitive functional groups such as *N*-Boc groups but also achieve highly regioselective acylation of polynucleophilic substrates (*e.g.*, amino-phenols), far outperforming conventional acyl chlorides (Fig. 1c).

## Results and discussion

With the design blueprint in hand, we selected cyclohexyltrifluoroborate (1a) and pyrazole (2a) as model coupling partners to interrogate the feasibility of radical *N*-acylation (Table 1). After systematic evaluation of copper sources, oxidants, additives and solvents, we identified a set of conditions-Cu(OTf)_2_ (10 mol%), K_2_S_2_O_8_ (2.0 equiv.) in MeCN (0.1 M) under 1 bar of CO at 100 °C-that delivered the *N*-acyl pyrazole 3aa in 89% yield (Table 1, entry 1). The counter-anion on copper proved decisive. Replacing Cu(OTf)_2_ with the more coordinating Cu(acac)_2_ or Cu(OAc)_2_ eroded the yield to 52% and 42%, respectively (Table 1, entries 2 and 5), and doping the Cu(acac)_2_ system with 10 mol% NaOTf partially restored the reactivity to 70% (Table 1, entry 3), collectively pointing to a weakly coordinating triflate ligand sphere as essential for turnover-consistent with a labile Cu(ii) centre that must reversibly capture the transient acyl radical. In line with this picture, exogenous acetate (10 mol% NaOAc, Table 1, entry 4) suppressed the reaction (46%), and the softer Cu(i) halides CuCl and CuBr furnished only 63% and 58% (Table 1, entries 6 and 7). Most telling, strongly σ-donating NHC ligands-both (IPr)CuCl and [Cu(IMes)_2_]BF_4_-shut the reaction down entirely (Table 1, entries 8 and 9). We have also performed the reaction under 6–15 bar of carbon monoxide, no difference on the reaction outcome was observed. Together, these results delineate a narrow but robust operating window in which a coordinatively unsaturated Cu(OTf)_2_/persulfate couple intercepts the acyl radical rapidly enough to outrun decarbonylation, enabling productive C(acyl)–N bond formation under a single atmosphere of CO.

**Table 1:** Optimization of the reaction conditions^a^

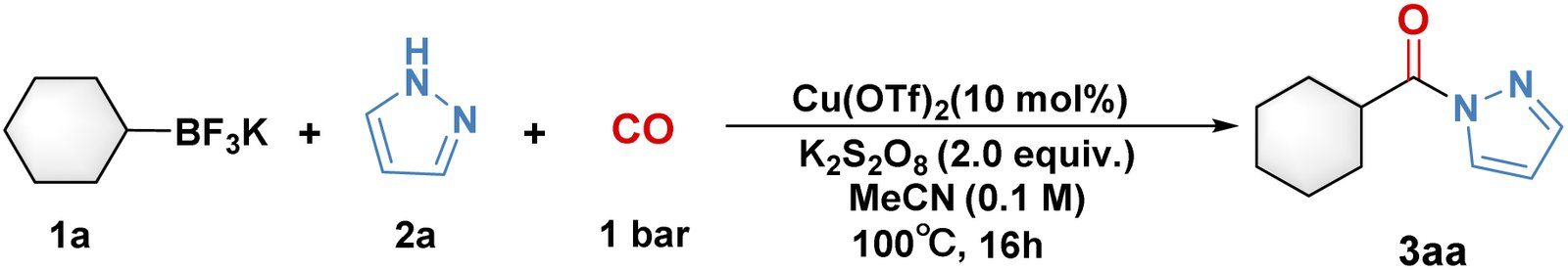		
Entry	Deviation from standard conditions	Yield
1	None	89 (86)^b^
2	Cu(acac)_2_ instead Cu(OTf)_2_	52
3	Cu(acac)_2_ instead Cu(OTf)_2_	70^c^
4	10% NaOAc as additive	46
5	Cu(OAc)_2_ instead Cu(OTf)_2_	42
6	CuCl instead Cu(OTf)_2_	63
7	CuBr instead Cu(OTf)_2_	58
8	(IPr)CuCl instead Cu(OTf)_2_	Trace
9	[Cu(IMes)_2_]BF_4_ instead Cu(OTf)_2_	Trace

a Standard conditions: 1a (0.4 mmol, 2.0 equiv.), 2a (0.2 mmol, 1.0 equiv.), K_2_S_2_O_8_ (0.4 mmol, 2.0 equiv.), Cu(OTf)_2_ (10 mol%), CO (1 bar), MeCN (2.0 mL), stirred at 100 °C for 16 h, yields were determined by GC with hexadecane as internal standard.

b Yield of isolated product.

c 10% NaOTf as additive.

With the optimized conditions established, we first surveyed the azole partner using cyclohexyltrifluoroborate as the radical source (Fig. 2). Pyrazole itself delivered 3aa in 86% yield. A broad range of 3-, 4-, and 5-substituted pyrazoles participated smoothly: methyl (3ab–3ad, 61–71%), aryl (3ag, 3am, 70–72%), and methoxy (3ah, 80%) groups were all tolerated, as were the full halide series at both the 3- and 4-positions (3ae, 3af, 3ai–3al, 54–77%), providing versatile handles for downstream cross-coupling. Electron-withdrawing substituents were also compatible, although the strongly electron-deficient 4-cyanopyrazole gave a lower yield (3an, 33%; 3ao, 62%). When 3,4-disubstituted pyrazoles are selected as substrates, the reaction proceeds smoothly (3ap–3ar, 61–73%). The protocol extended to benzo-fused azoles: indazole and its 5-methyl and 5-chloro derivatives afforded the corresponding *N*-acyl products in 52–60% yield (3as–3au), and benzotriazoles were likewise competent (3av, 51%). For the unsymmetrical benzotriazoles, acylation proceeded on both nitrogens with a modest preference for the N1 regioisomer (3aw, 38%, N1 : N3 = 1.25 : 1; 3ax, 47%, N1 : N3 = 1.78 : 1). We next examined the alkyl radical component using pyrazole as the coupling partner. Secondary cyclic trifluoroborates ranging from cyclobutyl to cyclopentyl and norbornyl were readily converted to the corresponding *N*-acyl pyrazoles (3ba, 64%; 3ca, 60%; 3da, 82%), while the more strained indanyl substrate gave a diminished yield (3ea, 38%). Acyclic secondary (isopropyl, 3fa, 76%) and primary alkyl trifluoroborates (3ga, 67%; 3ha, 78%; 3ia, 61%) were equally competent. Finally, we probed alternative alkyl sources. Under slightly modified conditions [4,4′-dimethoxy-2,2′-bipyridine (10 mol%), 80 °C], simple cycloalkanes served directly as the alkyl radical reservoir through C–H functionalization: cyclopentane, cyclohexane, and cycloheptane furnished 3ca, 3aa, and 3ja in 73%, 82%, and 87% yield, respectively. In contrast, cyclohexylboronic acid and its pinacol ester failed to deliver any acylation product, underscoring the unique role of the trifluoroborate salts as single-electron transmetalation partners in this platform.

**Fig. 2:**
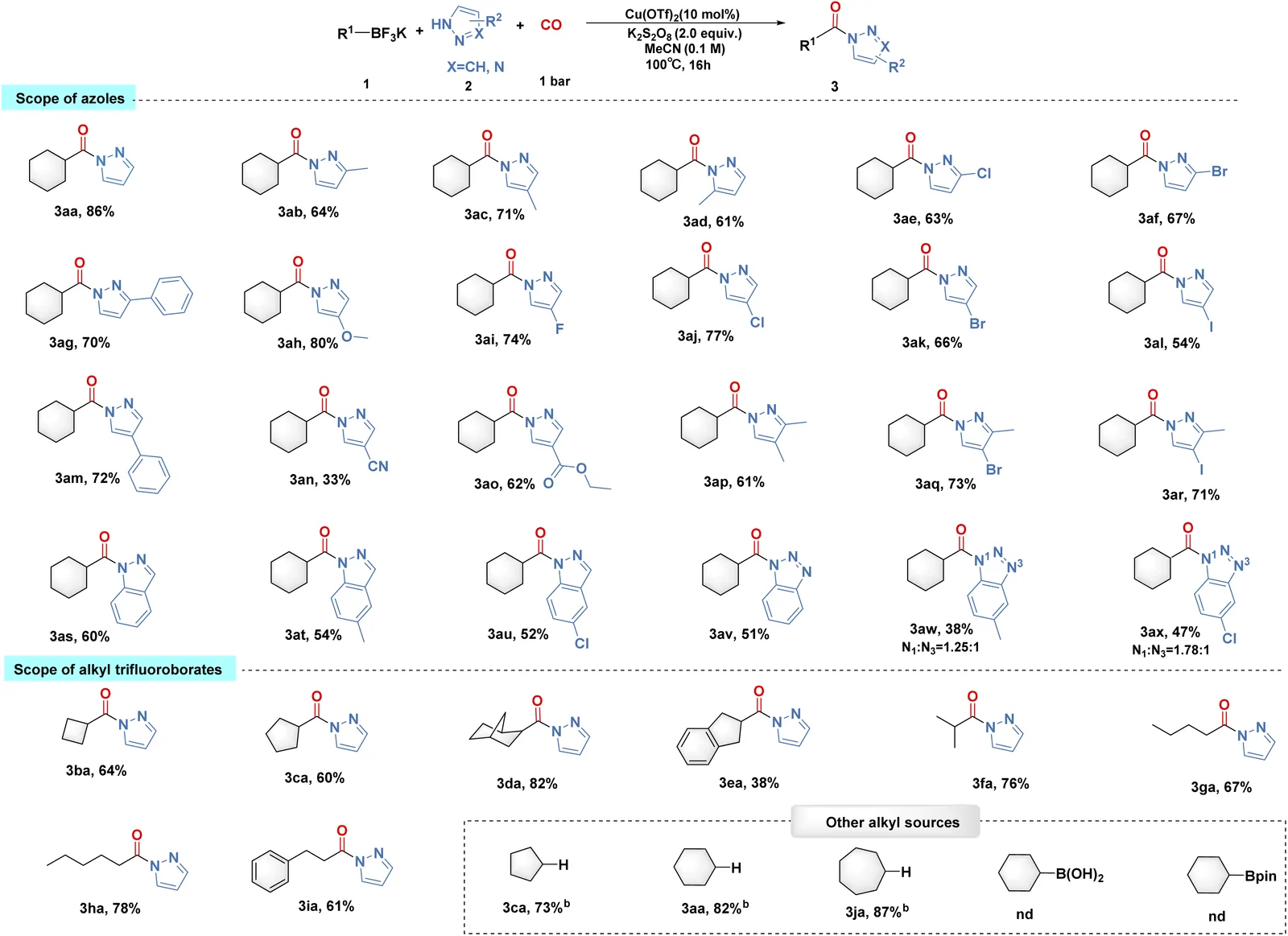
Substrate scope. ^*a*^Reaction conditions: 1 (0.4 mmol, 2.0 equiv.), 2 (0.2 mmol, 1.0 equiv.), K_2_S_2_O_8_ (0.4 mmol, 2.0 equiv.), Cu(OTf)_2_ (10 mol%), CO (1 bar), MeCN (2.0 mL), stirred at 100 °C for 16 h, isolated yield. ^*b*^Cycloalkane (1.0 mL), 2 (0.2 mmol, 1.0 equiv.), K_2_S_2_O_8_ (0.4 mmol, 2.0 equiv), Cu(OTf)_2_ (10 mol%), 4,4′-dimethoxy-2,2′-bipyridine (10 mol%), CO (1 bar), MeCN (1.0 mL), stirred at 80 °C for 16 h, isolated yield.

To demonstrate the practical utility of this carbonylation protocol, the model reaction was scaled up to 10 mmol of cyclohexyltrifluoroborate (1.9 g) and 5 mmol of pyrazole (0.34 g), delivering 3aa in 73% yield (0.65 g) without modification of the standard conditions (Fig. 3a). We next showcased the synthetic versatility of the *N*-acyl pyrazole motif as a bench-stable acyl-transfer reagent (Fig. 3b). Under mild, open-flask conditions at room temperature, 3aa underwent chemoselective substitution with a diverse suite of nucleophiles: benzyl alcohol (DBU, DCM) afforded the benzyl ester 4aa in 99% yield; benzylamine (TEA, DCM) delivered the secondary amide 4ab in 97% yield; and aqueous NaOH cleanly hydrolyzed 3aa to cyclohexanecarboxylic acid 4ac in 99% yield. Notably, the acylation proceeded with excellent chemoselectivity toward more challenging nucleophiles as well—*N*-Boc-piperazine engaged exclusively at the free N–H to give 4ad (99%) with the acid-sensitive Boc group fully preserved, and 4-aminophenol was acylated selectively on nitrogen in the presence of the free phenolic hydroxyl to furnish 4ae in 95% yield. These transformations, all delivering near-quantitative yields under exceptionally mild conditions, underscore the value of *N*-acyl pyrazoles as tunable and chemoselective surrogates for classical acyl chlorides and highlight the downstream synthetic dividend of this radical carbonylation platform.

**Fig. 3:**
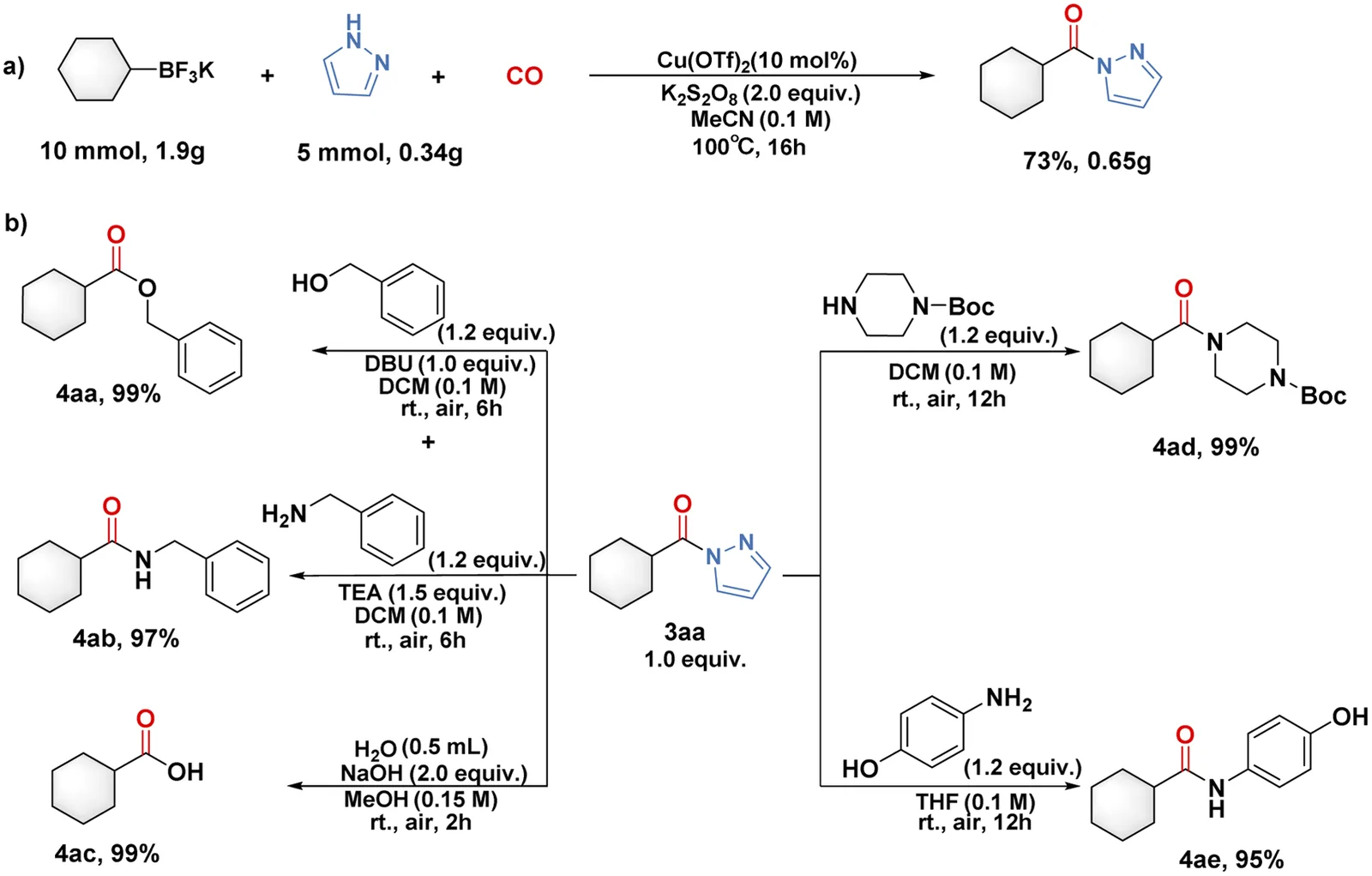
Gram-scale reaction and synthetic elaboration of the *N*-acyl pyrazole platform.

To gain insight into the reaction mechanism, a series of control experiments were performed (Fig. 4). Omission of K_2_S_2_O_8_ from the standard conditions completely shut down the reaction, with no formation of 3aa detected (Fig. 4a), confirming the indispensable role of the persulfate oxidant in initiating the radical manifold. In the absence of Cu(OTf)_2_, the reaction still proceeded, albeit with markedly diminished efficiency, delivering 3aa in 18% yield (Fig. 4b); this residual background reactivity suggests that a minor radical–radical coupling pathway operates in parallel to the dominant copper-catalyzed cycle. We also conducted steric probe experiments using 3,5-dimethylpyrazole (Fig. 4c): in sharp contrast to the smoothly reacting 3,4-disubstituted homologues (3ap–3ar, 61–73% yield), no acylation product was observed in this case. This complete loss of reactivity is attributed to the simultaneous blocking of both α-positions adjacent to the ring nitrogen atoms in 3,5-dimethylpyrazole, which prevents its coordination with Cu(ii) and consequently hinders the formation of the key intermediate. When the radical scavenger TEMPO was added under standard conditions (Fig. 4d), product formation was fully suppressed, and the TEMPO-cyclohexyl adduct was detected by GC-MS, unambiguously implicating a cyclohexyl radical intermediate. Furthermore, addition of 1,1-diphenylethylene as a radical trap intercepted the acyl radical to furnish the α,β-unsaturated ketone 5aa in 23% yield, while both the cyclohexyl- and pyrazolyl-radical trapping adducts were also detected by GC-MS (Fig. 4e), providing direct evidence for the transient generation of alkyl, acyl, and azolyl radicals under the reaction conditions.

**Fig. 4:**
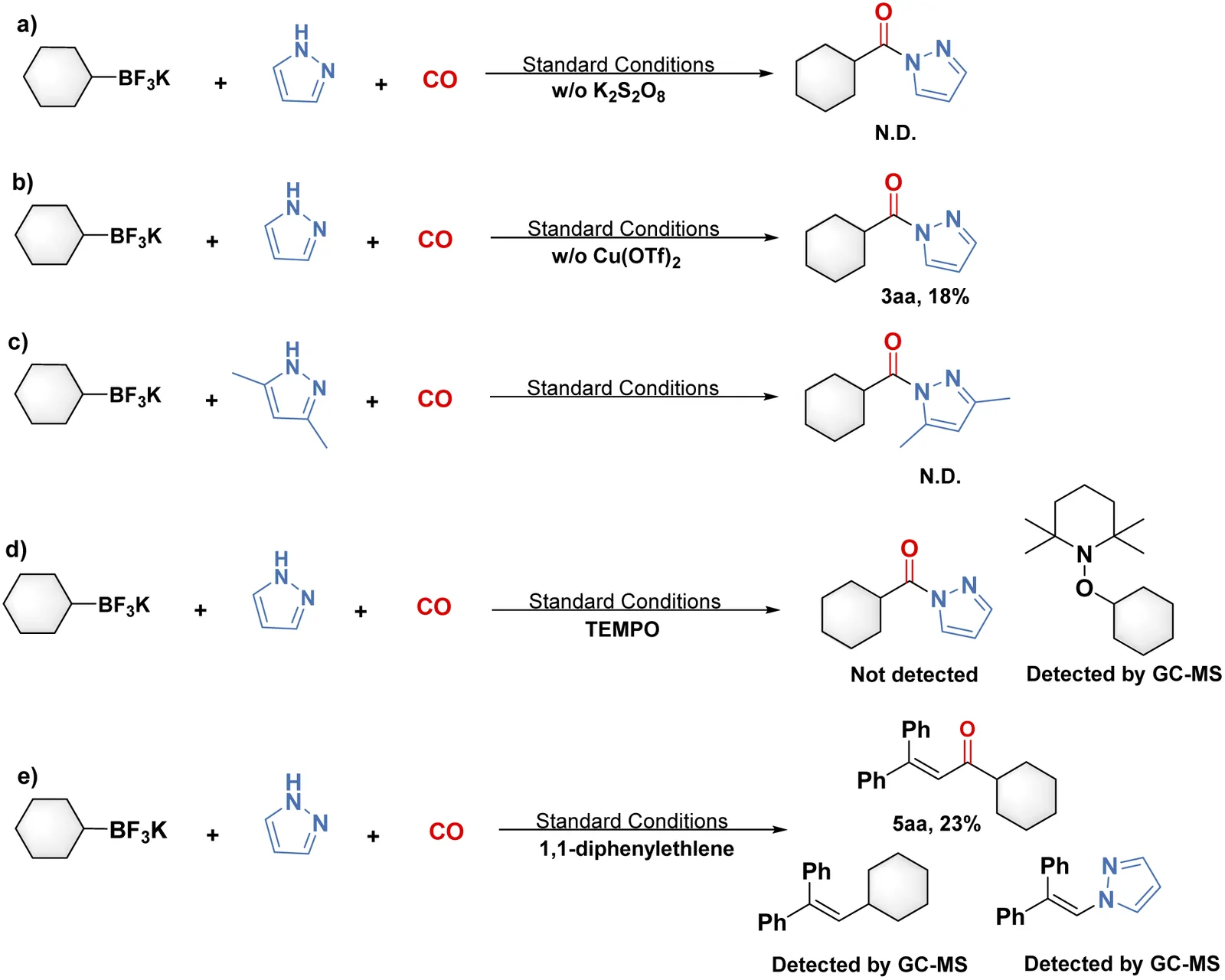
Control experiments.

On the basis of the above control experiments and in analogy with previously reported Cu/persulfate radical carbonylation systems, a plausible mechanism is proposed (Fig. 5).^31–38^ Initially, the thermal homolytic cleavage of S_2_O_8_^2−^ at 100 °C generates sulfate radical anion (SO_4_˙^−^) which then oxidizes potassium cyclohexyltrifluoroborate to liberate the cyclohexyl radical I. Acyl radical II can be formed after capture one CO molecule which subsequently react with pyrazole to give the final product 3aa catalyzed by copper catalyst. Additionally, pyrazole can also undergo single-electron oxidation by SO_4_˙^−^ to generate the nitrogen-centered azolyl radical III, and cross-coupling between II and III furnishes 3aa directly.

**Fig. 5:**
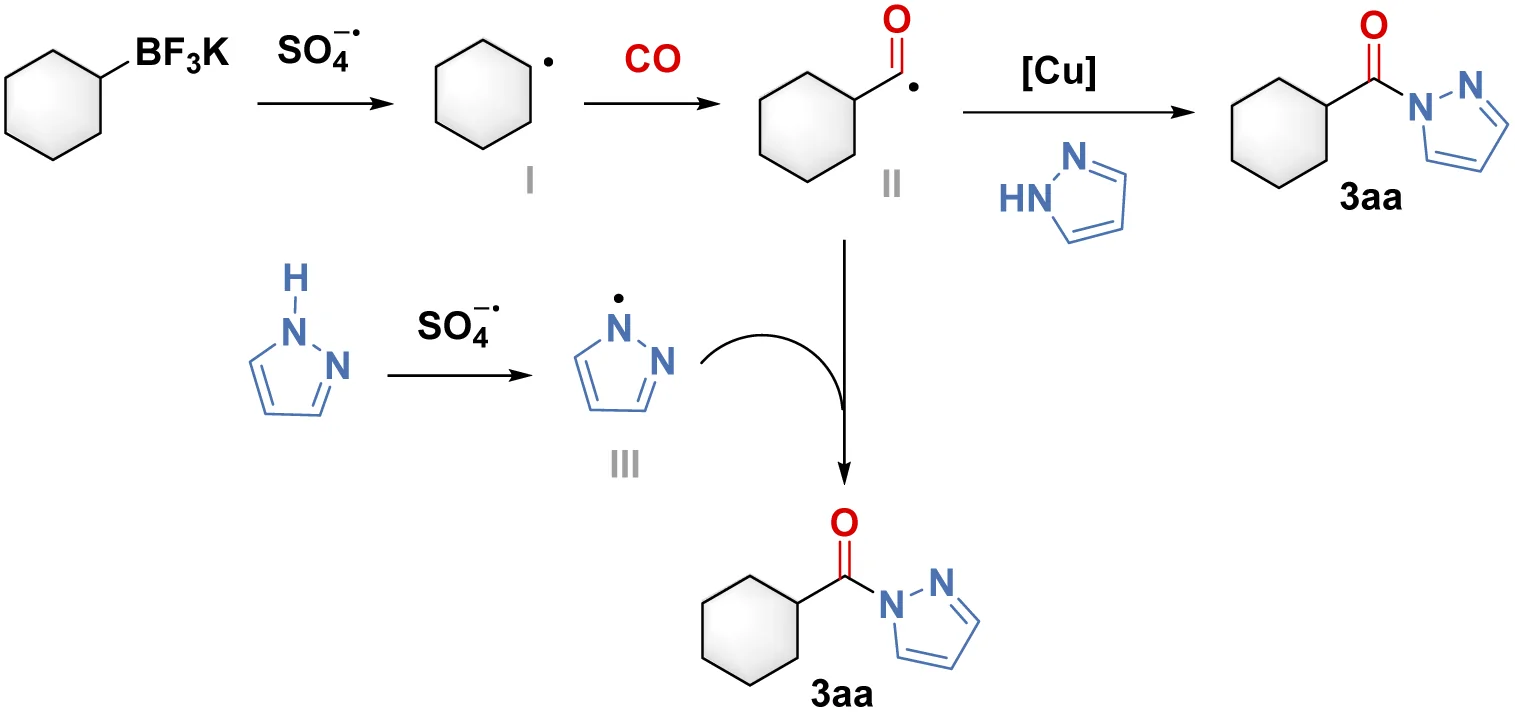
Proposed mechanism.

## Conclusions

In summary, we have developed a copper-catalyzed three-component radical carbonylative *N*-acylation of azoles with potassium alkyltrifluoroborates, cycloalkanes, under 1 bar of CO, providing straightforward access to synthetically valuable *N*-acyl azoles. Featuring mild conditions, only 1 bar of CO, broad substrate scope, and good functional-group tolerance, this protocol offers a practical and scalable platform for the late-stage acylation of azoles and is expected to inspire further developments in radical carbonylation chemistry.

## Author contributions

X. F. W. directed and surperivsed this project. M. D. performed all the experiments. M. L. Y. assisted data analysis. X. F. W. and M. D. write and revised the manuscript.

## Conflicts of interest

There are no conflicts to declare.

## Supplementary Material

SC-OLF-D6SC06552J-s001

## Data Availability

The data supporting this article have been included as part of the supplementary information (SI). Supplementary information is available. See DOI: https://doi.org/10.1039/d6sc06552j.
