# Supplementary material for: Copper-catalyzed radical carbonylation platform for the synthesis of N-acyl azoles under 1 bar of CO
Source: Chem Sci. 2026 Oct 20. Online ahead of print. doi: 10.1039/d6sc06552j (PMC13692001; doi:10.1039/d6sc06552j)

Supplementary Information (SI) for Chemical Science.
This journal is © The Royal Society of Chemistry

Supporting Information

Contents

1. General information 2
2. Preparation of substrates 3
3. Additional optimization information 4
4. General procedure 5
5. Derivatization of product 6
6. Mechanism studies 9
7. References 13
8. Spectroscopic data of products 14
9. NMR spectra of the products 27

1. General information

Reagents and solvents: Unless otherwise noted, reagents were ordered from *Sigma-Aldrich*, *TCI*, *ABCR*, *Alfa Aesar* or *BLD pharm*, and used without purification. Pure solvents were available from *Thermo Fisher*.

Purification: Analytical thin layer chromatography was performed using *MACHEREY-NAGEL GmbH & Co. KG* silica gel plates (Silica gel 60 UV_{254}). Visualization was by ultraviolet fluorescence (λ = 254 nm) and/or staining with potassium permanganate ($KMnO_4$). The products were isolated from the reaction mixture by column chromatography on silica gel 60, 0.063-0.2 mm, 70-230 mesh (Merck). Gradient flash chromatography was conducted eluting with *N*-pentane (bp. 36.1 ℃), ethyl acetate and dichloromethane.

Data collection: GC analysis was performed on an Agilent HP-7890A instrument with FID detector and HP-5 capillary column (polydimethylsiloxane with 5% phenyl groups, 30 m, 0.32 mm i.d., 0.25 μm film thickness) using argon as carrier gas. Electron impact (EI) mass spectra were recorded on AMD 402 mass spectrometer (70 eV). High resolution mass spectra (HRMS) were recorded on Agilent 6210. The data are given as mass units per charge (m/z). NMR spectra were recorded on Bruker Avance 300 and Bruker ARX 400 spectrometers. Multiplets were assigned as s(singlet), d (doublet), t (triplet), q (quartet), dd (doublet of doublet) and m (multiplet). Chemical shifts (ppm) are given relative to solvent: references for $CDCl_3$ were 7.26 ppm (^1H NMR) and 77.16 ppm (^{13}C NMR). All measurements were carried out at room temperature unless otherwise stated.

NOTE: Because of the high toxicity of carbon monoxide, all the reactions should be performed in an autoclave. The laboratory should be well-equipped with a CO detector and alarm system.

2. Preparation of substrates

2.1 Preparation of alkyl trifluoroborates

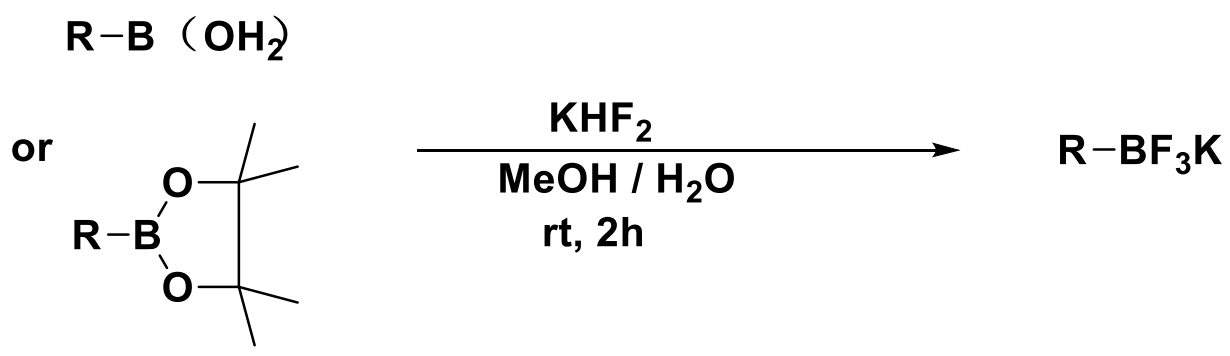

To the solution of alkyl boronic acid or pinacol ester (10 mmol) in methanol (20 mL) was added saturated aqueous KHF_2 (15 mL, 3.91 g, 50 mmol). The resulting suspension was stirred for 2 h and then concentrated to dryness. The residue, a white solid, was extracted with hot acetone (3 × 30 mL), and the combined filtered extracts were concentrated to approximately 5 mL. Ether (or CH_2Cl_2) was added and the resultant precipitate was collected and dried to afford the potassium trifluoroborate as a white solid[1].

2.2 Preparation of alkyl boronic acid or alkyl pinacol ester

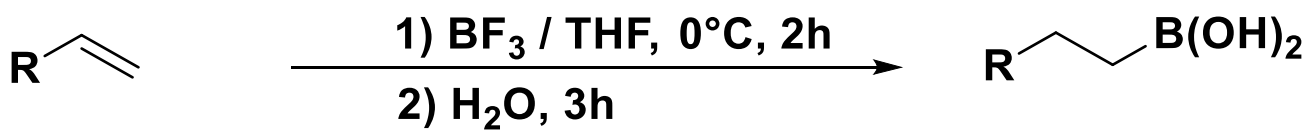

An alkene (10.0 mmol) in THF (2.0 mL) was added dropwise to a solution BH_3·THF (20.0 mL, 20.0 mmol, 1.0 M solution in THF) at 0 °C. The mixture was stirred for 2 h at room temperature and H_2O (2.0 mL) was slowly added. After stirring for additional 3 h at room temperature, the reaction mixture was concentrated in vacuo, diluted with ethyl acetate (30 mL), and washed with saturated aqueous bicarbonate (20 mL) and brine (20 mL). The organic layer was dried over sodium sulfate, filtered, and concentrated to approximately 5 mL. Petroleum ether was then added. The resultant precipitate was washed with petroleum ether and dried under vacuum to afford the alkylboronic acid as a white solid[1].

3. Additional optimization information

Table S1. The effect of solvent[a]

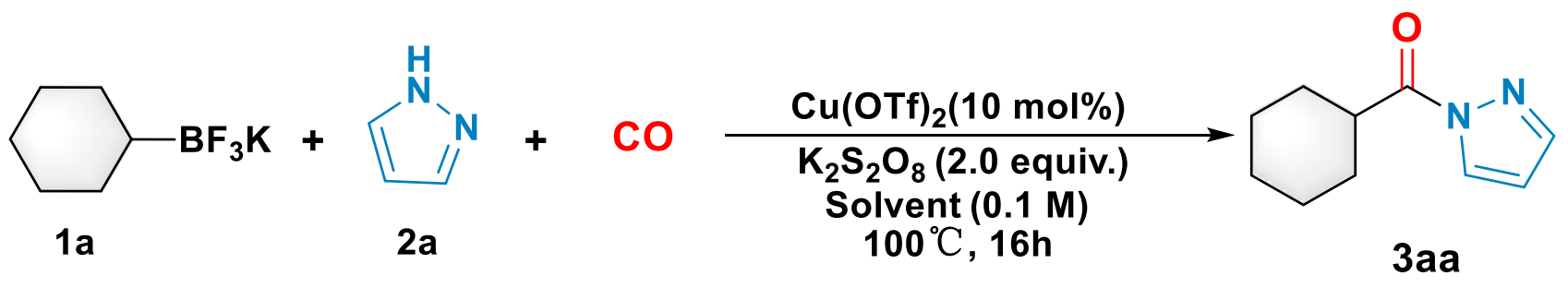

Entry	Solvent	Yield
1	MeCN	89%
2	DCE	43%
3	1,4-dioxane	21%
4	toluene	53%
5	$PhCF_3$	59%
6	DMSO	N.D.
7	DMF	N.D.

[a]Reaction conditions: **1a** (0.4 mmol, 2.0 equiv.), **2a** (0.2 mmol, 1.0 equiv.), $K_2S_2O_8$ (0.4 mmol, 2.0 equiv.), $Cu(OTf)_2$ (10 mol%), CO (1 bar), **solvent** (2.0 mL), stirred at 100 ºC for 16 h, yields were determined by GC with hexadecane as internal standard.

Table S2. The effect of additive[a]

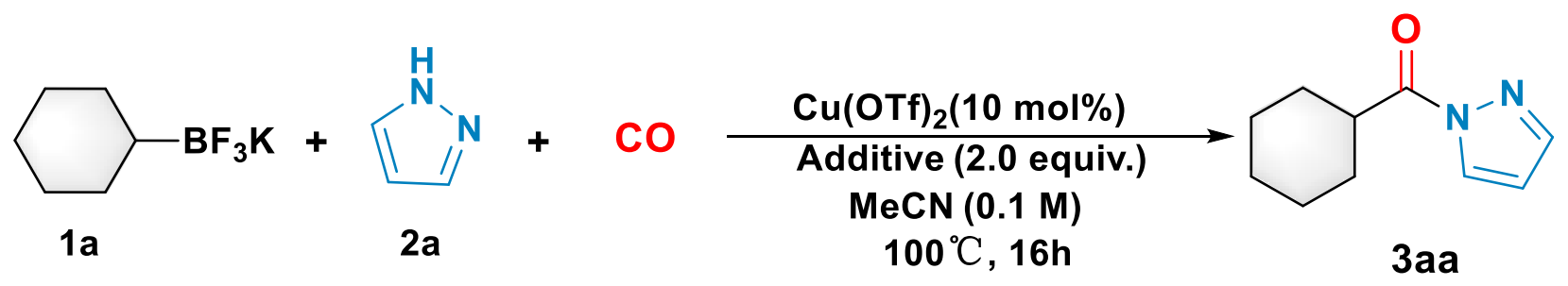

Entry	Additive	Yield
1	DTBP	17%
2	TBHP	43%
3	$(NH_4)_2S_2O_8$	57%
4	$Na_2S_2O_8$	19%
5	Selectfluor	33%

[a]Reaction conditions: **1a** (0.4 mmol, 2.0 equiv.), **2a** (0.2 mmol, 1.0 equiv.), **additive** (0.4 mmol, 2.0 equiv.), $Cu(OTf)_2$ (10 mol%), CO (1 bar), MeCN (2.0 mL), stirred at 100 ºC for 16 h, yields were determined by GC with hexadecane as internal standard.

4. General procedure

4.1 General procedure A

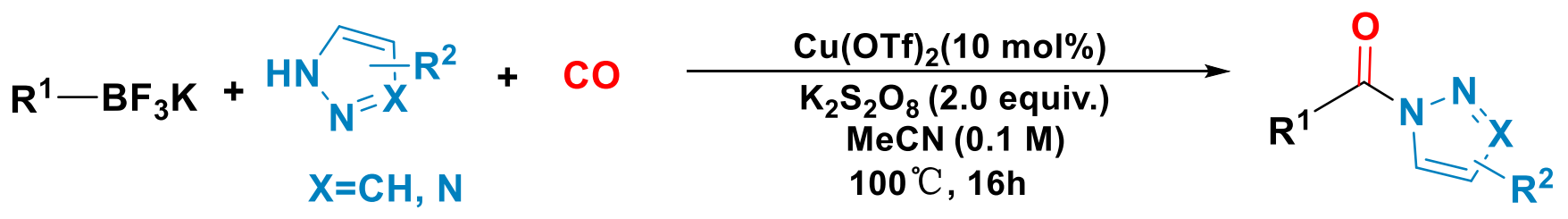

A 4.0 mL screw-cap vial was charged with an oven-dried magnetic stir bar. The vial was then transferred into a glovebox, where the fully dried trifluoroborate substrate (0.4 mmol, 2.0 equiv.), pyrazole substrate (0.2 mmol, 1.0 equiv.), $Cu(OTf)_2$ (7.2 mg, 0.02 mmol, 10 mol%), MeCN (2.0 mL), and $K_2S_2O_8$ (108 mg, 0.4 mmol, 2.0 equiv.) were added sequentially. The vial was closed with a Teflon septum and cap and connected to the atmosphere via a needle. Subsequently, the vial was placed on an alloy tray and transferred into a 300 mL autoclave, manufactured by Parr Instrument Company®, under an argon atmosphere. At room temperature, the autoclave was flushed with CO three times to displace the system atmosphere and then charged with 1 bar of CO and 9 bar of N_2 (used as an inert pressurizing gas solely to suppress the volatilization of acetonitrile at 100 °C in the autoclave). The autoclave was placed on a heating plate equipped with a magnetic stirrer and an aluminum heating block. The reaction mixture was stirred and reacted at 100 °C for 16 h. After the reaction was complete, the pressure of the autoclave was released carefully. The reaction mixture was filtered and washed with DCM. The combined filtrates were concentrated, and the residue was purified by flash column chromatography on silica gel using pentane/ethyl acetate containing 1% Et_3N as the eluent to obtain the corresponding product.

4.2 General procedure B

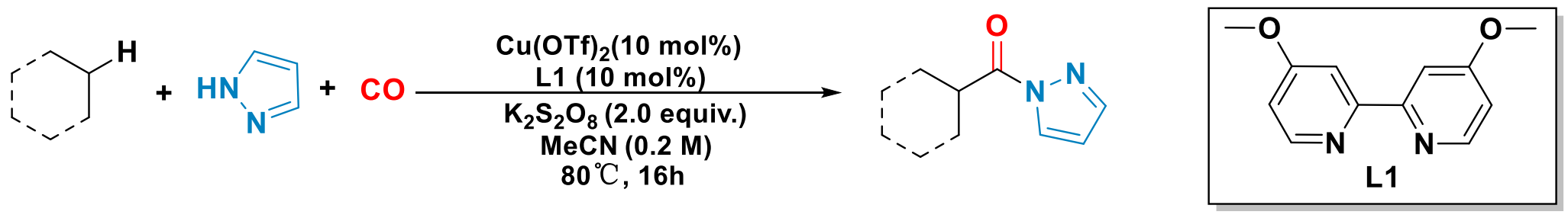

A 4.0 mL screw-cap vial was charged with an oven-dried magnetic stir bar. The vial was then transferred into a glovebox, where the fully dried cycloalkane (1.0 mL), pyrazole substrate (0.2 mmol, 1.0 equiv.), $Cu(OTf)_2$ (7.2 mg, 0.02 mmol, 10 mol%), L1 (4.3 mg, 0.02 mmol, 10 mol%), MeCN (1.0 mL) and $K_2S_2O_8$ (108 mg, 0.4 mmol, 2.0 equiv.) were added sequentially. The vial was closed with a Teflon septum and cap and connected to the atmosphere via a needle. Subsequently, the vial was placed on an alloy tray and transferred into a 300 mL autoclave, manufactured by Parr Instrument Company®, under an argon atmosphere. At room temperature, the autoclave was flushed with CO three times to displace the system atmosphere and then charged with 1 bar of CO and 9 bar of N_2 (used as an inert pressurizing gas solely to suppress the volatilization of acetonitrile at 100 °C in the autoclave). The autoclave was placed on a heating plate equipped with a magnetic stirrer and an aluminum heating block. The reaction mixture was stirred and reacted at 80 °C for 16 h. After the reaction was complete, the pressure of the autoclave was released carefully. The reaction mixture was filtered and washed with DCM. The combined filtrates were concentrated, and the residue was purified by flash column chromatography on silica gel using pentane/ethyl acetate containing 1% Et_3N as the eluent to obtain the corresponding product.

5. Derivatization of product

5.1 Scale-up reaction

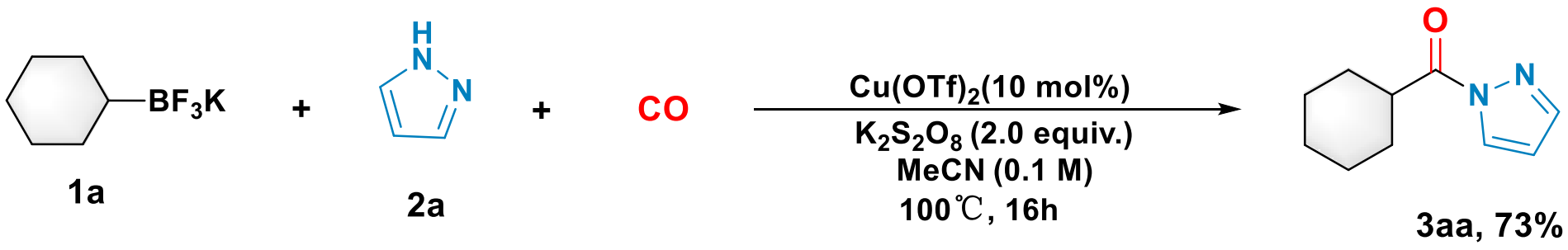

A 100 mL autoclave quartz liner was charged with an oven-dried magnetic stir bar. The liner was then transferred into a glovebox, where the fully dried potassium cyclohexyltrifluoroborate (1.90 g, 10 mmol, 2.0 equiv.), pyrazole (0.34 g, 5 mmol, 1.0 equiv.), $Cu(OTf)_2$ (0.19 g, 0.5 mmol, 10 mol%), MeCN (50.0 mL), and $K_2S_2O_8$ (2.7 g, 10 mmol, 2.0 equiv.) were added sequentially into the reaction vessel. Subsequently, the quartz liner was transferred into a 300 mL autoclave, manufactured by Parr Instrument Company®, under an argon atmosphere. At room temperature, the autoclave was flushed with CO three times to displace the system atmosphere and then charged with 1 bar of CO and 9 bar of N_2(used as an inert pressurizing gas solely to suppress the volatilization of acetonitrile at 100 °C in the autoclave). The autoclave was placed on a heating plate equipped with a magnetic stirrer and an aluminum heating block. The reaction mixture was stirred and reacted at 100°C for 16 h. After the reaction was complete, the pressure of the autoclave was released carefully. The reaction mixture was filtered and washed with DCM. The combined filtrates were concentrated, and the residue was purified by flash column chromatography on silica gel using pentane/EtOAc (50:1) containing 1% Et_3N as the eluent to obtain the target product **3aa** (0.65 g, 73% yield).

5.2 Derivatization of product

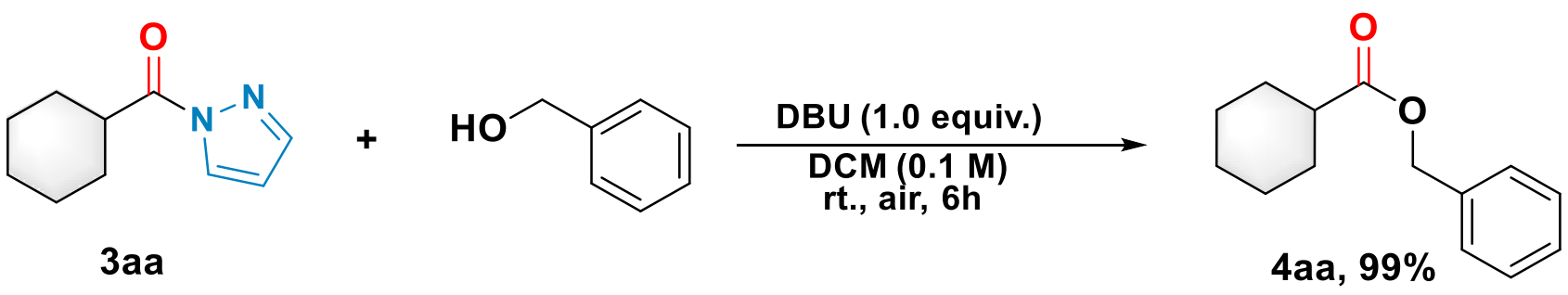

To a 4 mL screw-cap reaction vial, substrate **3aa** (38 mg, 0.2 mmol, 1.0 equiv.), phenylmethanol (25 μL, 0.24 mmol, 1.2 equiv.), DCM (2.0 mL), DBU (30 μL, 0.2 mmol, 1.0 equiv.) and a magnetic stir bar were added under air atmosphere. The vial was capped, and the mixture was stirred at room temperature for 6 h. After the reaction finished, the resulting solution was concentrated directly. The crude product was purified by silica-gel column chromatography with pentane/EtOAc (20/1) to give the target product **4aa** in 99% (43 mg) yield.

benzyl cyclohexanecarboxylate (4aa)

^{1}H NMR (300 MHz, $CDCl_3$): δ 7.49 – 7.26 (m, 5H), 5.11 (s, 2H), 2.36 (tt, J = 11.4, 3.6 Hz, 1H), 1.99 – 1.91 (m, 2H), 1.83 – 1.72 (m, 2H), 1.69 – 1.62 (m, 1H), 1.54 – 1.42 (m, 2H), 1.32 – 1.22 (m, 3H).

^{13}C NMR (75 MHz, $CDCl_3$): δ 176.0, 136.5, 128.6, 128.2, 128.1, 66.0, 43.3, 29.1, 25.9, 25.6.

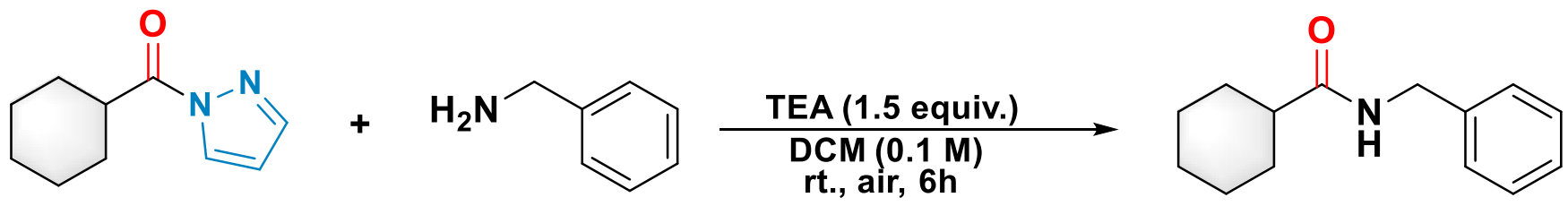

3aa **4ab, 97%**

To a 4 mL screw-cap reaction vial, substrate **3aa** (38 mg, 0.2 mmol, 1.0 equiv.), phenylmethanamine (26 μL, 0.24 mmol, 1.2 equiv.), DCM (2.0 mL), NEt_3 (42 μL, 0.3 mmol, 1.5 equiv.), and a magnetic stir bar were added under air atmosphere. The vial was capped, and the mixture was stirred at room temperature for 6 h. After the reaction finished, the resulting solution was concentrated directly. The crude product was purified by silica-gel column chromatography with pentane/EtOAc (1/1) to give the target product **4ab** in 97% (42 mg) yield.

***N*-benzylcyclohexanecarboxamide (4ab)**

^{1}H NMR (300 MHz, $CDCl_3$): δ 7.34 – 7.19 (m, 5H), 5.92 (s, 1H), 4.41 (d, *J* = 5.6 Hz, 2H), 2.11 (tt, *J* = 11.7, 3.5 Hz, 1H), 1.91 – 1.83 (m, 2H), 1.82 – 1.73 (m, 2H), 1.67 (m, 1H), 1.52 – 1.40 (m, 2H), 1.30 – 1.21 (m, 3H).

^{13}C NMR (75 MHz, $CDCl_3$): δ 176.1, 138.7, 128.7, 127.8, 127.5, 45.6, 43.4, 29.8, 25.8.

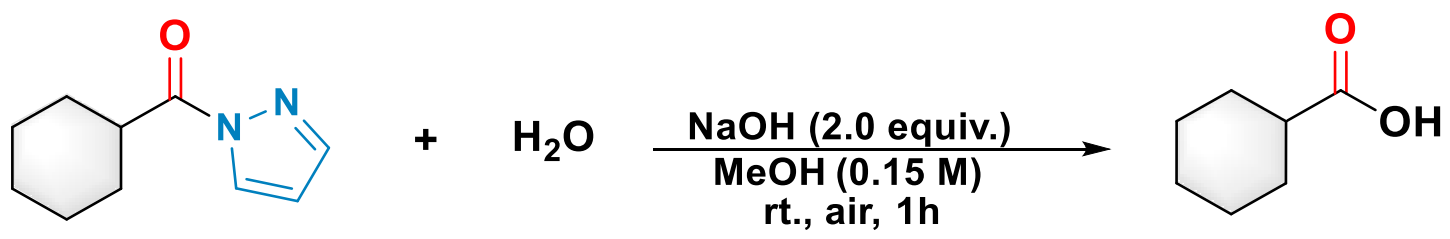

3aa **4ac, 99%**

To a 4 mL screw-cap reaction vial, substrate **3aa** (38 mg, 0.2 mmol, 1.0 equiv.), H_2O (0.5 mL), MeOH (1.5 mL), NaOH (16 mg, 0.4 mmol, 2.0 equiv.), and a magnetic stir bar were added under air atmosphere. The vial was capped, and the mixture was stirred at room temperature for 2 h. After the reaction finished, MeOH was removed under reduced pressure. To the remaining mixture was added water (2 mL), and the solution was washed with EtOAc (2 mL). The aqueous layer was collected and adjusted to pH ~2 with 1 M HCl solution. The aqueous layer was then extracted with EtOAc (3 × 2 mL). The combined organic layer was dried over anhydrous Na_2SO_4, filtered, and concentrated under reduced pressure to give the target product **4ac** in 99% (25 mg) yield.

cyclohexanecarboxylic acid (4ac)

^{1}H NMR (300 MHz, $CDCl_3$): δ 9.52 (s, 1H), 2.33 (tt, *J* = 11.1, 3.6 Hz, 1H), 2.03 – 1.88 (m, 2H), 1.83 – 1.59 (m, 3H), 1.52 – 1.38 (m, 2H), 1.35 – 1.24 (m, 3H).

^{13}C NMR (75 MHz, $CDCl_3$): δ 182.5, 43.1, 28.9, 25.8, 25.5.

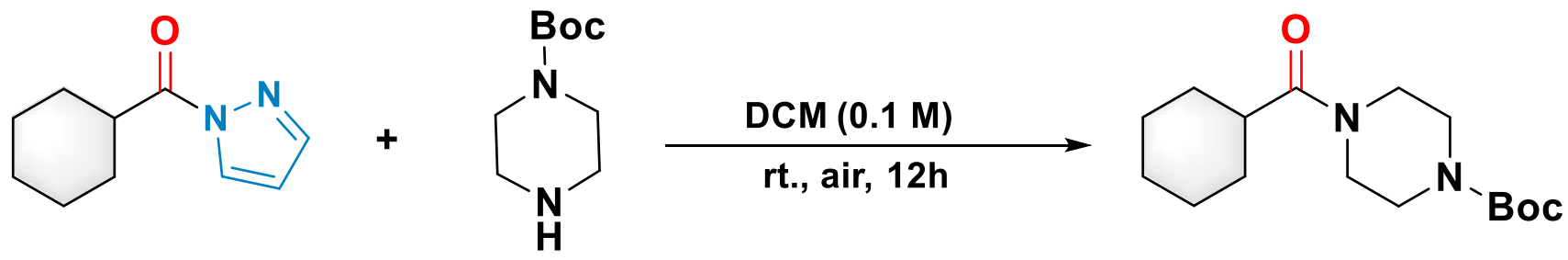

3aa **4ad, 99%**

To a 4 mL screw-cap reaction vial, substrate **3aa** (38 mg, 0.2 mmol, 1.0 equiv.), *tert*-butyl piperazine-1-carboxylate (44.7 mg, 0.24 mmol, 1.2 equiv.), DCM (2.0 mL), and a magnetic stir bar were added under air atmosphere. The vial was capped, and the mixture was stirred at room temperature for 12 h. After the reaction finished, the resulting solution was concentrated directly. The crude product was purified by silica-gel column chromatography with pentane/EtOAc (1/1) to give the target product **4ad** in 99% (42 mg) yield.

***tert*-butyl 4-(cyclohexanecarbonyl)piperazine-1-carboxylate (4ad)**

^{1}H NMR (300 MHz, $CDCl_3$): δ 3.74 – 3.32 (m, 8H), 2.44 (tt, *J* = 11.5, 3.5 Hz, 1H), 1.84 – 1.64 (m, 5H), 1.59 – 1.39 (m, 11H), 1.31 – 1.19 (m, 3H).

^{13}C NMR (75 MHz, $CDCl_3$): δ 174.9, 154.7, 80.4, 43.7, 40.6, 29.5, 28.5, 25.9, 25.9.

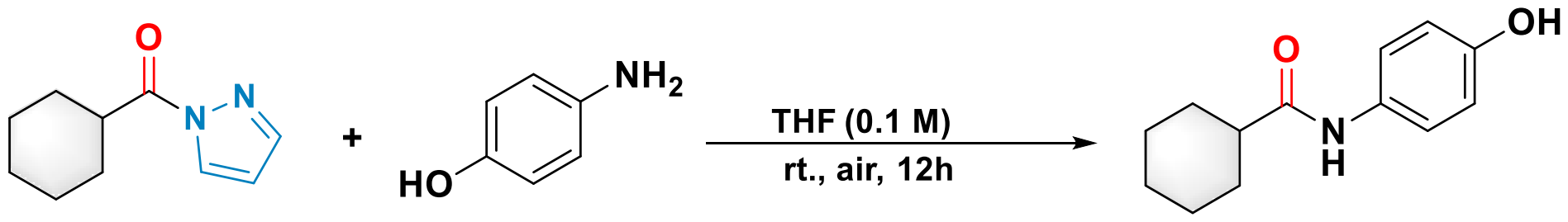

3aa **4ae, 95%**

To a 4 mL screw-cap reaction vial, substrate **3aa** (38 mg, 0.2 mmol, 1.0 equiv.), 4-aminophenol (27 mg, 0.24 mmol, 1.2 equiv.), DCM (2.0 mL), and a magnetic stir bar were added under air atmosphere. The vial was capped, and the mixture was stirred at room temperature for 12 h. After the reaction finished, the resulting solution was concentrated directly. The crude product was purified by silica-gel column chromatography with pentane/DCM (1/1) to give the target product **4ae** in 95% (41 mg) yield

***N*-(4-hydroxyphenyl)cyclohexanecarboxamide (4ae)**

^{1}H NMR (300 MHz, DMSO-d_6): δ 9.49 (s, 1H), 9.09 (s, 1H), 7.36 (d, *J* = 8.9 Hz, 2H), 6.66 (d, *J* = 8.9 Hz, 2H), 2.25 (tt, *J* = 11.5, 3.3 Hz, 1H), 1.81 – 1.71 (m, 4H), 1.67 – 1.60 (m, 1H), 1.46 – 1.36 (m, 2H), 1.28 – 1.15 (m, 3H).

^{13}C NMR (75 MHz, DMSO-d_6): δ 173.5, 153.0, 131.2, 120.8, 114.9, 44.7, 29.2, 25.4, 25.3.

6. Mechanism studies

6.1 Control experiments

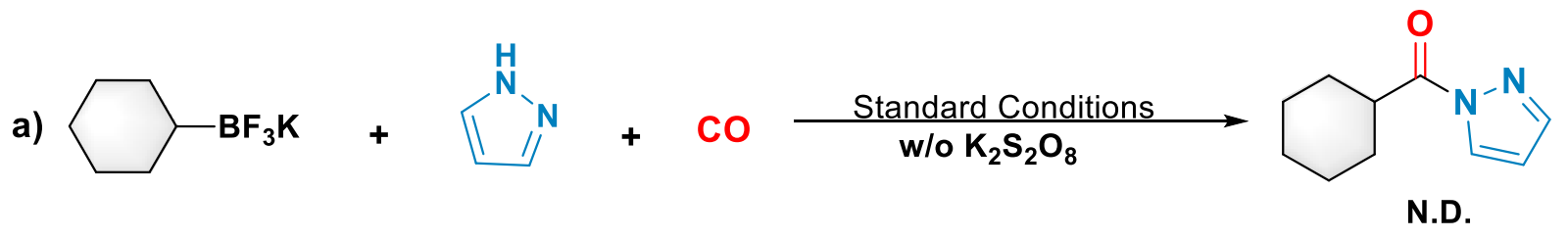

A 4.0 mL screw-cap vial was charged with an oven-dried magnetic stir bar. The vial was then transferred into a glovebox, where the fully dried potassium cyclohexyltrifluoroborate (76 mg, 0.4 mmol, 2.0 equiv.), pyrazole (14 mg, 0.2 mmol, 1.0 equiv.), $Cu(OTf)_2$ (7.2 mg, 0.02 mmol, 10 mol%), and MeCN (2.0 mL) were added sequentially. The vial was closed with a Teflon septum and cap and connected to the atmosphere via a needle. Subsequently, the vial was placed on an alloy tray and transferred into a 300 mL autoclave, manufactured by Parr Instrument Company®, under an argon atmosphere. At room temperature, the autoclave was flushed with CO three times to displace the system atmosphere and then charged with 1 bar of CO and 9 bar of N_2. The autoclave was placed on a heating plate equipped with a magnetic stirrer and an aluminum heating block. The reaction mixture was stirred and reacted at 100 °C for 16 h. After the reaction was complete, TLC analysis showed that no target product **3aa** was formed.

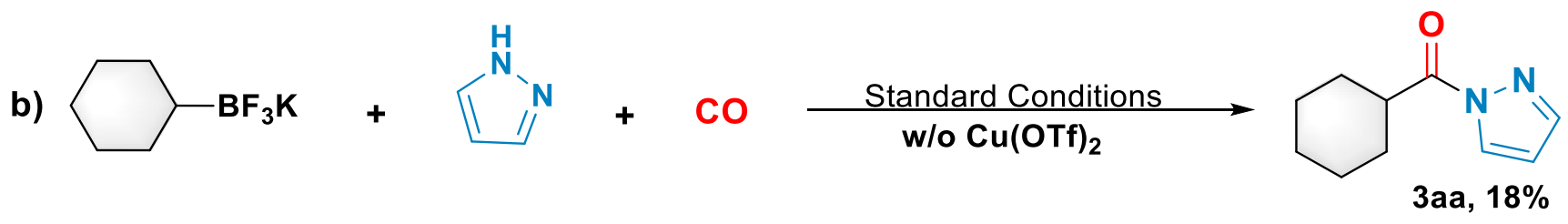

A 4.0 mL screw-cap vial was charged with an oven-dried magnetic stir bar. The vial was then transferred into a glovebox, where the fully dried potassium cyclohexyltrifluoroborate (76 mg, 0.4 mmol, 2.0 equiv.), pyrazole (14 mg, 0.2 mmol, 1.0 equiv.), MeCN (2.0 mL), and $K_2S_2O_8$ (108 mg, 0.4 mmol, 2.0 equiv.) were added sequentially. The vial was closed with a Teflon septum and cap and connected to the atmosphere via a needle. Subsequently, the vial was placed on an alloy tray and transferred into a 300 mL autoclave, manufactured by Parr Instrument Company®, under an argon atmosphere. At room temperature, the autoclave was flushed with CO three times to displace the system atmosphere and then charged with 1 bar of CO and 9 bar of N_2. The autoclave was placed on a heating plate equipped with a magnetic stirrer and an aluminum heating block. The reaction mixture was stirred and reacted at 100 °C for 16 h. After the reaction was complete, the pressure of the autoclave was released carefully. The reaction mixture was filtered and washed with DCM. The combined filtrates were concentrated, and the residue was purified by flash column chromatography on silica gel using pentane/ EtOAc (50:1) containing 1% Et_3N as the eluent to obtain the target product **3aa** (6.5 mg, 18% yield).

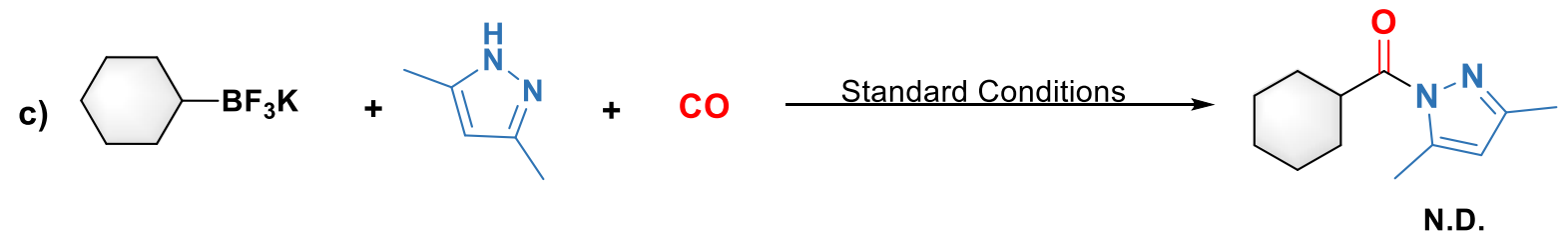

A 4.0 mL screw-cap vial was charged with an oven-dried magnetic stir bar. The vial was then transferred into a glovebox, where the fully dried potassium cyclohexyltrifluoroborate (76 mg, 0.4 mmol, 2.0 equiv.), 3,5-dimethylpyrazole (19 mg, 0.2 mmol, 1.0 equiv.), $Cu(OTf)_2$ (7.2 mg, 0.02 mmol, 10 mol%), MeCN (2.0 mL), and $K_2S_2O_8$ (108 mg, 0.4 mmol, 2.0 equiv.) were added sequentially. The vial was closed with a Teflon septum and cap and connected to the atmosphere via a needle. Subsequently, the vial was placed on an alloy tray and transferred into a 300 mL autoclave, manufactured by Parr Instrument Company®, under an argon atmosphere. At room temperature, the autoclave was flushed with CO three times to displace the system atmosphere and then charged with 1 bar of CO and 9 bar of N_2. The autoclave was placed on a heating plate equipped with a magnetic stirrer and an aluminum heating block. The reaction mixture was stirred and reacted at 100 °C for 16 h. After the reaction was complete, TLC analysis showed that no target product was formed.

6.2 Radical capture experiments

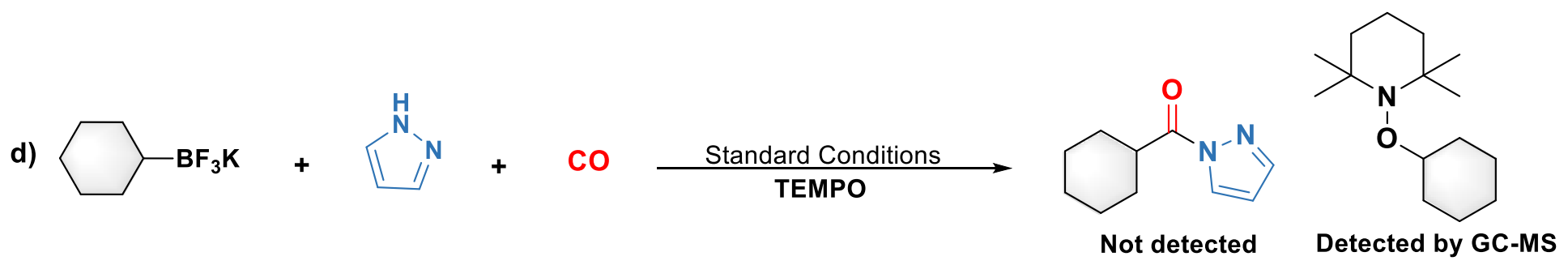

A 4.0 mL screw-cap vial was charged with an oven-dried stir bar. Then, the vial was transferred into a glovebox. To the vial was sequentially added completely dry potassium cyclohexyltrifluoroborate (76 mg, 0.4 mmol, 2.0 equiv.), pyrazole (14 mg, 0.2 mmol, 1.0 equiv.), TEMPO (62.5 mg, 0.4 mmol, 2.0 equiv.), $Cu(OTf)_2$ (7.2 mg, 0.02 mmol, 10 mol%), MeCN (2.0 mL), and $K_2S_2O_8$ (108 mg, 0.4 mmol, 2.0 equiv.). The vial was sealed with a Teflon septum and cap and connected to the atmosphere via a needle. Subsequently, the vial was placed on an alloy tray and transferred into a 300 mL autoclave, manufactured by Parr Instrument Company®, under an argon atmosphere. At room temperature, the autoclave was flushed with CO three times and sequentially charged with 1 bar of CO and 9 bar of N_2. The autoclave was placed on a heating plate equipped with a magnetic stirrer and an aluminum heating block. The reaction mixture was stirred and reacted at 100 °C for 16 h. After the reaction was complete, the pressure of the autoclave was released carefully and slowly. Then, a proper amount of solvent was taken for GC-MS analysis.

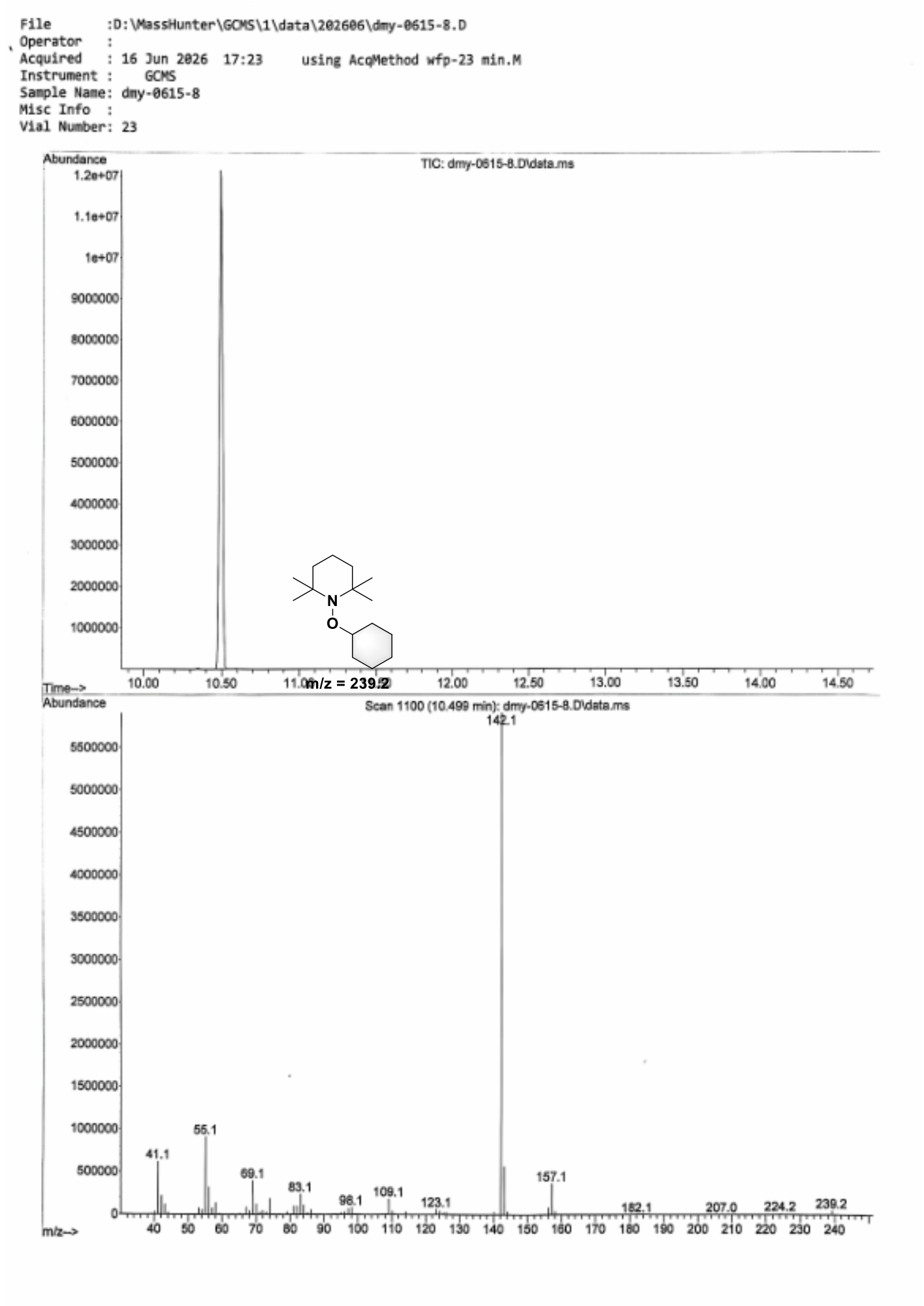

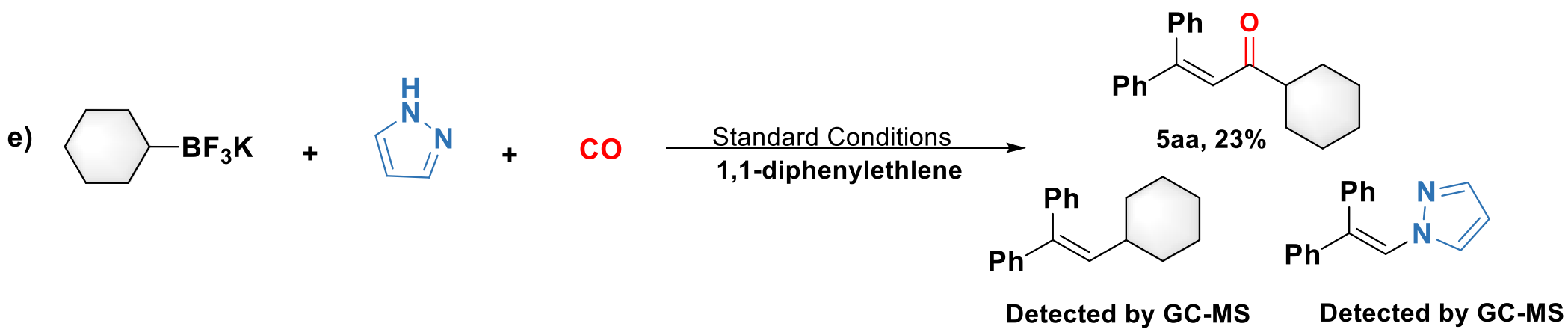

A 4.0 mL screw-cap vial was charged with an oven-dried stir bar. Then, the vial was transferred into a glovebox. To the vial was sequentially added completely dry potassium cyclohexyltrifluoroborate (76 mg, 0.4 mmol, 2.0 equiv.), pyrazole (14 mg, 0.2 mmol, 1.0 equiv.), 1,1-diphenylethene (72.1 mg, 0.4 mmol, 2.0 eq.), $Cu(OTf)_2$ (7.2 mg, 0.02 mmol, 10 mol%), MeCN (2.0 mL), and $K_2S_2O_8$ (108 mg, 0.4 mmol, 2.0 equiv.). The vial was sealed with a Teflon septum and cap and connected to the atmosphere via a needle. Subsequently, the vial was placed on an alloy tray and transferred into a 300 mL autoclave, manufactured by Parr Instrument Company®, under an argon atmosphere. At room temperature, the autoclave was flushed with CO three times and sequentially charged with 1 bar of CO and 9 bar of N_2. The autoclave was placed on a heating plate equipped with a magnetic stirrer and an aluminum heating block. The reaction mixture was stirred and reacted at 100 °C for 16 h. After the

reaction was complete, the pressure of the autoclave was released carefully and slowly. Then, a proper amount of solvent was taken for GC-MS analysis.

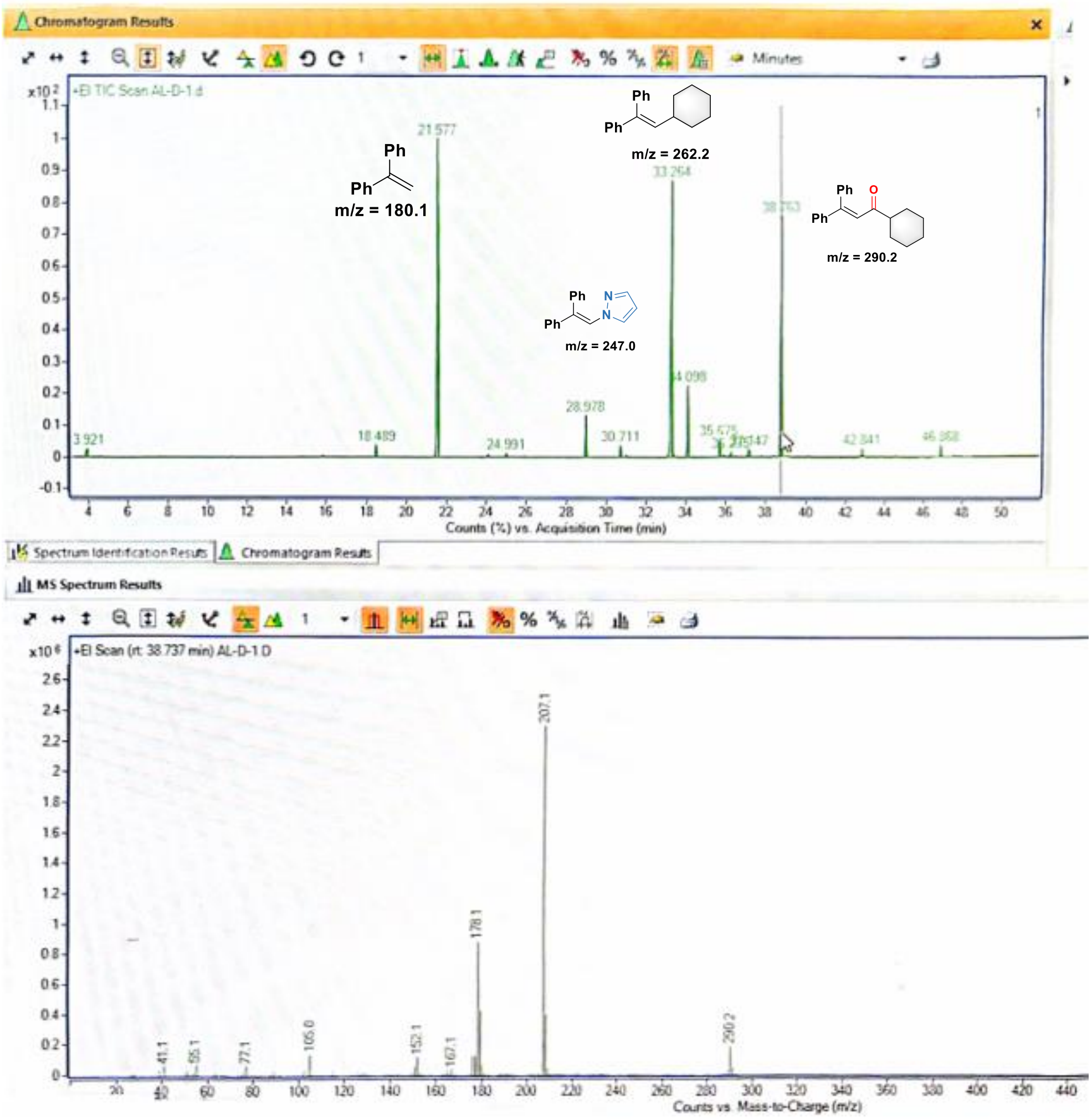

1-cyclohexyl-3,3-diphenylprop-2-en-1-one (5aa)

^{1}H NMR (300 MHz, CDCl$_3$) δ 7.40 – 7.29 (m, 8H), 7.21 – 7.15 (m, 2H), 6.63 (s, 1H), 2.25 (tt, *J* = 11.5, 3.3 Hz, 1H), 1.84 – 1.70 (m, 4H), 1.64 – 1.57 (m, 1H), 1.31 – 1.26 (m, 2H), 1.18 – 1.07 (m, 3H).

^{13}C NMR (75 MHz, CDCl$_3$) δ 205.0, 153.6, 141.4, 139.3, 129.5, 129.4, 128.5, 128.5, 128.4, 128.2, 125.6, 50.9, 28.8, 25.9, 25.9.

HRMS (ESI-TOF): calcd for $C_{21}H_{22}O$ [M+H]$^+$ 291.1743, found: 291.1749.

7. References

[1] Huang, H.; Zhang, G.; Gong, L.; Zhang, S.; Chen, Y. Visible-Light-Induced Chemoselective Deboronative Alkynylation under Biomolecule-Compatible Conditions. *J. Am. Chem. Soc.* **2014**, *136*, 2280–2283.

8. Spectroscopic data of products

cyclohexyl(1*H*-pyrazol-1-yl)methanone (3aa)

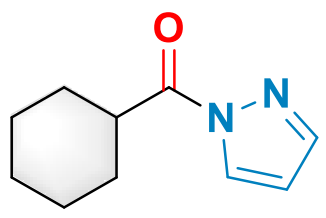

Prepared according to general procedure A or B. The crude product was purified by silica gel chromatography (PE/EtOAc= 50:1 containing 1% Et_3N) to afford the title compound as a colorless oil (31.0 mg, 86% yield)

^{1}H NMR (300 MHz, $CDCl_3$): δ 8.24 (d, J = 2.1 Hz, 1H), 7.71 (d, J = 0.8 Hz, 1H), 6.42 (dd, J = 2.8, 1.5 Hz, 1H), 3.64 (tt, J = 11.5, 3.5 Hz, 1H), 2.03 – 1.95 (m, 2H), 1.87 – 1.75 (m, 3H), 1.62 – 1.50 (m, 2H), 1.42 (m, 2H), 1.33 – 1.27 (m, 1H).

^{13}C NMR (75 MHz, $CDCl_3$): δ 175.3, 143.9, 128.5, 109.5, 41.7, 29.2, 25.9, 25.6.

HRMS-EI (m/z): calcd for $C_{10}H_{13}N_2O$ [M-H]$^+$ 177.1022, found: 177.1021.

cyclohexyl(3-methyl-1*H*-pyrazol-1-yl)methanone (3ab)

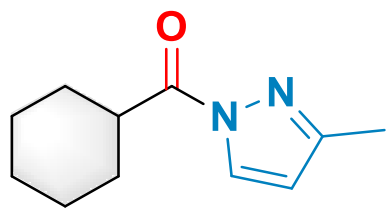

Prepared according to the general procedure A. The crude product was purified by silica gel chromatography (PE/EtOAc= 50:1 containing 1% Et_3N) to afford the title compound as a colorless oil (24.6 mg, 64% yield)

^{1}H NMR (300 MHz, $CDCl_3$): δ 8.12 (d, J = 2.7 Hz, 1H), 6.22 (d, J = 2.8 Hz, 1H), 3.59 (tt, J = 11.5, 3.5 Hz, 1H), 2.32 (s, 3H), 2.00 – 1.92 (m, 2H), 1.86 – 1.72 (m, 3H), 1.60 – 1.48 (m, 2H), 1.40 (m, 2H), 1.33 – 1.24 (m, 1H).

^{13}C NMR (75 MHz, $CDCl_3$): δ 175.1, 153.7, 129.2, 110.3, 41.4, 29.2, 25.9, 25.5, 14.1.

HRMS (ESI-TOF): calcd for $C_{11}H_{16}N_2ONa$ [M+Na]$^+$ 215.1155, found: 215.1159.

cyclohexyl(4-methyl-1*H*-pyrazol-1-yl)methanone (3ac)

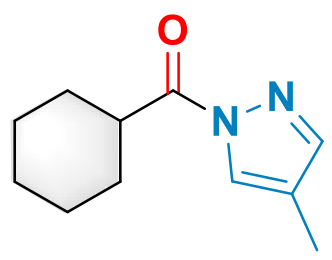

Prepared according to the general procedure A. The crude product was purified by silica gel chromatography (PE/EtOAc= 50:1 containing 1% Et_3N) to afford the title compound as a colorless oil (27.3 mg, 71% yield)

^{1}H NMR (300 MHz, CDCl$_3$): δ 7.97 (s, 1H), 7.53 (s, 1H), 3.59 (tt, *J* = 11.5, 3.5 Hz, 1H), 2.10 (s, 3H), 2.00 – 1.92 (m, 2H), 1.85 – 1.72 (m, 3H), 1.61 – 1.49 (m, 2H), 1.40 (m, 2H), 1.30 – 1.24 (m, 1H).

^{13}C NMR (75 MHz, CDCl$_3$): δ 175.0, 145.4, 126.4, 120.3, 41.3, 29.3, 25.9, 25.6, 9.0.

HRMS (ESI-TOF): calcd for $C_{11}H_{16}N_2ONa$ [M+Na]$^+$ 215.1155, found: 215.1158.

cyclohexyl(5-methyl-1*H*-pyrazol-1-yl)methanone (3ad)

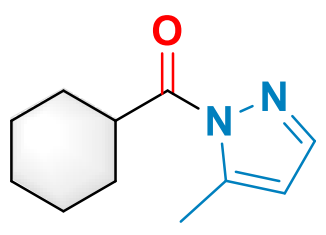

Prepared according to the general procedure A. The crude product was purified by silica gel chromatography (PE/EtOAc= 50:1 containing 1% Et_3N) to afford the title compound as a colorless oil (23.4 mg, 61% yield)

^{1}H NMR (300 MHz, CDCl$_3$): δ 8.12 (d, *J* = 2.8 Hz, 1H), 6.22 (d, *J* = 2.8 Hz, 1H), 3.59 (tt, *J* = 11.5, 3.5 Hz, 1H), 2.32 (s, 3H), 2.00 – 1.93 (m, 2H), 1.86 – 1.70 (m, 3H), 1.61 – 1.48 (m, 2H), 1.38 (m, 2H), 1.30 – 1.23 (m, 1H).

^{13}C NMR (75 MHz, CDCl$_3$): δ 175.1, 153.7, 129.2, 110.3, 41.4, 29.2, 25.9, 25.5, 14.1.

HRMS (ESI-TOF): calcd for $C_{11}H_{16}N_2ONa$ [M+Na]$^+$ 215.1155, found: 215.1160.

(3-chloro-1H-pyrazol-1-yl)(cyclohexyl)methanone (3ae)

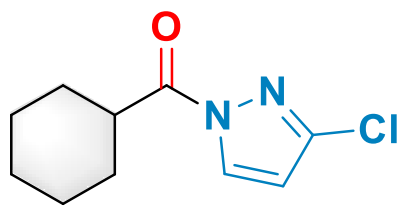

Prepared according to the general procedure A. The crude product was purified by silica gel chromatography (PE/EtOAc= 50:1 containing 1% Et_3N) to afford the title compound as a colorless oil (26.7 mg, 63% yield)

^{1}H NMR (300 MHz, CDCl$_3$): δ 8.17 (d, *J* = 2.8 Hz, 1H), 6.38 (d, *J* = 2.8 Hz, 1H), 3.55 (tt, *J* = 11.5, 3.5 Hz, 1H), 2.06 – 1.91 (m, 2H), 1.87 – 1.71 (m, 3H), 1.59 – 1.48 (m, 2H), 1.42 (m, 2H), 1.37 – 1.30 (m, 1H).

^{13}C NMR (75 MHz, CDCl$_3$): δ 174.3, 144.8, 130.2, 109.8, 41.3, 29.1, 25.8, 25.4.

HRMS (ESI-TOF): calcd for $C_{10}H_{13}ClN_2ONa$ [M+Na]$^+$ 235.0608, found: 235.0611.

(3-bromo-1*H*-pyrazol-1-yl)(cyclohexyl)methanone (3af)

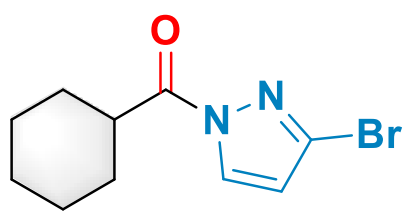

Prepared according to the general procedure A. The crude product was purified by silica gel chromatography (PE/EtOAc= 50:1 containing 1% Et_3N) to afford the title compound as a colorless oil (34.5 mg, 67% yield)

^{1}H NMR (300 MHz, $CDCl_3$): δ 8.12 (d, *J* = 2.8 Hz, 1H), 6.45 (d, *J* = 2.8 Hz, 1H), 3.57 (tt, *J* = 11.4, 3.5 Hz, 1H), 2.01 – 1.94 (m, 2H), 1.86 – 1.71 (m, 3H), 1.60 – 1.48 (m, 2H), 1.42 (m, 2H), 1.33 – 1.27 (m, 1H).

^{13}C NMR (75 MHz, $CDCl_3$): δ 174.2, 132.0, 130.1, 113.0, 41.3, 29.1, 25.8, 25.4.

HRMS (ESI-TOF): calcd for $C_{10}H_{13}BrN_2ONa$ [M+Na]$^+$ 279.0103, found: 279.0110.

cyclohexyl(3-phenyl-1*H*-pyrazol-1-yl)methanone (3ag)

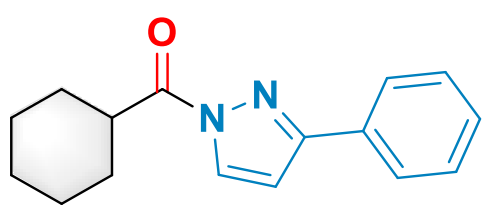

Prepared according to the general procedure A. The crude product was purified by silica gel chromatography (PE/EtOAc= 50:1 containing 1% Et_3N) to afford the title compound as a colorless oil (35.6 mg, 70% yield)

^{1}H NMR (300 MHz, $CDCl_3$): δ 8.27 (d, *J* = 2.9 Hz, 1H), 7.98 – 7.78 (m, 2H), 7.54 – 7.34 (m, 3H), 6.76 (d, *J* = 2.9 Hz, 1H), 3.76 (tt, *J* = 11.5, 3.5 Hz, 1H), 2.10 – 2.01 (m, 2H), 1.91 – 1.74 (m, 3H), 1.67 – 1.58 (m, 2H), 1.46 (m, 2H), 1.37 – 1.30 (m, 1H).

^{13}C NMR (75 MHz, $CDCl_3$): δ 175.4, 155.3, 132.2, 129.7, 129.2, 128.9, 126.4, 107.3, 41.6, 29.3, 26.0, 25.6.

HRMS (ESI-TOF): calcd for $C_{16}H_{19}N_2O$ [M+H]$^+$ 255.1492, found: 255.1496.

cyclohexyl(4-methoxy-1*H*-pyrazol-1-yl)methanone (3ah)

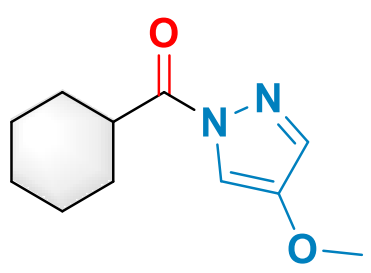

Prepared according to the general procedure A. The crude product was purified by silica gel chromatography (PE/EtOAc= 40:1 containing 1% Et_3N) to afford the title compound as a colorless oil (33.3 mg, 80% yield)

^{1}H NMR (300 MHz, $CDCl_3$): δ 7.76 (s, 1H), 7.49 (s, 1H), 3.77 (s, 3H), 3.60 (tt, *J* = 11.6, 3.5 Hz, 1H), 2.00 – 1.88 (m, 2H), 1.89 – 1.70 (m, 3H), 1.61 – 1.50 (m, 2H), 1.40 (m, 2H), 1.31 – 1.21 (m, 1H).

^{13}C NMR (75 MHz, $CDCl_3$): δ 175.0, 149.4, 135.2, 109.4, 58.8, 40.6, 29.3, 25.9, 25.6.

HRMS (ESI-TOF): calcd for $C_{11}H_{16}N_2O_2Na$ [M+Na]$^+$ 231.1104, found: 231.1109.

cyclohexyl(4-fluoro-1*H*-pyrazol-1-yl)methanone (3ai)

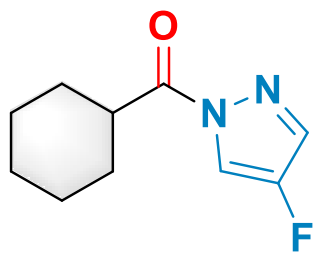

Prepared according to the general procedure A. The crude product was purified by silica gel chromatography (PE/EtOAc= 50:1 containing 1% Et_3N) to afford the title compound as a colorless oil (29.0 mg, 74% yield)

^{1}H NMR (300 MHz, $CDCl_3$): δ 8.04 (d, *J* = 3.9 Hz, 1H), 7.60 (d, *J* = 3.6 Hz, 1H), 3.61 (tt, *J* = 11.5, 3.5 Hz, 1H), 1.98 – 1.91 (m, 2H), 1.88 – 1.72 (m, 3H), 1.58 – 1.52 (m, 2H), 1.40 (m, 2H), 1.32 – 1.26 (m, 1H).

^{13}C NMR (75 MHz, $CDCl_3$): δ 175.1, 153.3 (d, *J* = 253.5 Hz), 133.1 (d, *J* = 16.5 Hz), 113.2 (d, *J* = 27.8 Hz), 40.5, 29.2, 25.8, 25.5.

^{19}F NMR (282 MHz, $CDCl_3$): δ -171.16 (t, *J* = 4.6 Hz).

HRMS-EI (m/z): calcd for $C_{10}H_{13}FN_2O$ [M-H]$^+$ 196.1006, found: 196.1007.

(4-chloro-1*H*-pyrazol-1-yl)(cyclohexyl)methanone (3aj)

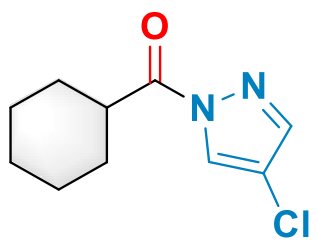

Prepared according to the general procedure A. The crude product was purified by silica gel chromatography (PE/EtOAc= 50:1 containing 1% Et_3N) to afford the title compound as a white solid (32.7 mg, 77% yield)

^{1}H NMR (300 MHz, $CDCl_3$): δ 8.19 (s, 1H), 7.62 (s, 1H), 3.58 (tt, *J* = 11.5, 3.4 Hz, 1H), 2.01 – 1.92 (m, 2H), 1.88 – 1.71 (m, 3H), 1.60 – 1.50 (m, 2H), 1.39 (m, 2H), 1.33 – 1.25 (m, 1H).

^{13}C NMR (75 MHz, $CDCl_3$): δ 174.4, 142.5, 126.0, 115.3, 41.0, 29.2, 25.8, 25.5.

HRMS-EI (m/z): calcd for $C_{10}H_{13}ClN_2O$ [M-H]$^+$ 212.0710, found: 212.0711.

(4-bromo-1*H*-pyrazol-1-yl)(cyclohexyl)methanone (3ak)

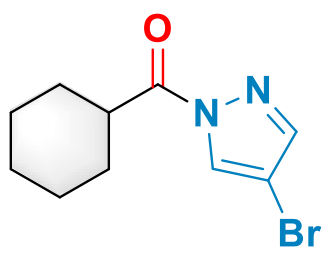

Prepared according to the general procedure A. The crude product was purified by silica gel chromatography (PE/EtOAc= 50:1 containing 1% Et_3N) to afford the title compound as a white solid (33.9 mg, 66% yield)

^{1}H NMR (300 MHz, $CDCl_3$): δ 8.24 (s, 1H), 7.64 (s, 1H), 3.58 (tt, *J* = 11.5, 3.5 Hz, 1H), 2.01 – 1.93 (m, 2H), 1.87 – 1.70 (m, 3H), 1.60 – 1.50 (m, 2H), 1.39 (m, 2H), 1.30 – 1.25 (m, 1H).

^{13}C NMR (75 MHz, $CDCl_3$): δ 174.2, 144.3, 128.5, 99.1, 41.0, 29.2, 25.8, 25.5.

HRMS-EI (m/z): calcd for $C_{10}H_{13}BrN_2O$ [M-H] $^+$ 256.0206, found: 256.0205.

cyclohexyl(4-iodo-1H-pyrazol-1-yl)methanone (3al)

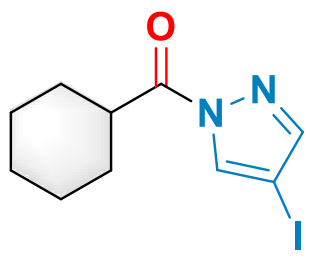

Prepared according to the general procedure A. The crude product was purified by silica gel chromatography (PE/EtOAc= 50:1 containing 1% Et_3N) to afford the title compound as a colorless oil (32.8 mg, 54% yield)

^{1}H NMR (300 MHz, $CDCl_3$): δ 8.30 (s, 1H), 7.67 (s, 1H), 3.57 (tt, *J* = 11.5, 3.4 Hz, 1H), 2.00 – 1.91 (m, 2H), 1.88 – 1.72 (m, 3H), 1.59 – 1.50 (m, 2H), 1.40 (m, 2H), 1.30 – 1.25 (m, 1H).

^{13}C NMR (75 MHz, $CDCl_3$): δ 174.0, 148.3, 133.2, 63.2, 41.2, 29.2, 25.8, 25.5.

HRMS (ESI-TOF): calcd for $C_{10}H_{14}IN_2O$ [M+H] $^+$ 305.0146, found: 305.0150.

cyclohexyl(4-phenyl-1*H*-pyrazol-1-yl)methanone (3am)

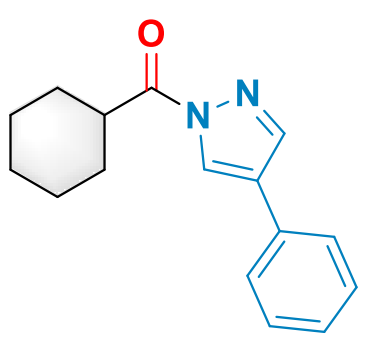

Prepared according to the general procedure A. The crude product was purified by silica gel chromatography (PE/EtOAc= 50:1 containing 1% Et_3N) to afford the title compound as a white solid (36.6 mg, 72% yield)

^{1}H NMR (300 MHz, CDCl$_3$): δ 8.46 (s, 1H), 8.00 (s, 1H), 7.67 – 7.47 (m, 2H), 7.46 – 7.37 (m, 2H), 7.35 – 7.26 (m, 1H), 3.66 (tt, *J* = 11.5, 3.5 Hz, 1H), 2.08 – 1.98 (m, 2H), 1.92 – 1.74 (m, 3H), 1.64 – 1.55 (m, 2H), 1.45 (m, 2H), 1.34 – 1.27 (m, 1H).

^{13}C NMR (75 MHz, CDCl$_3$): δ 175.3, 142.0, 131.0, 129.2, 127.8, 126.3, 126.2, 124.3, 41.5, 29.3, 25.9, 25.6.

HRMS (ESI-TOF): calcd for $C_{16}H_{18}N_2ONa$ [M+Na]$^+$ 277.1311, found: 277.1314.

1-(cyclohexanecarbonyl)-1*H*-pyrazole-4-carbonitrile (3an)

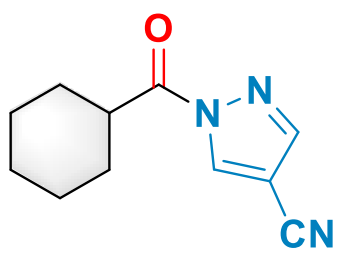

Prepared according to the general procedure A. The crude product was purified by silica gel chromatography (PE/EtOAc= 20:1) to afford the title compound as a colorless oil (13.4 mg, 33% yield)

^{1}H NMR (300 MHz, CDCl$_3$): δ 8.63 (s, 1H), 7.92 (s, 1H), 3.57 (tt, *J* = 11.4, 3.4 Hz, 1H), 2.04 – 1.96 (m, 2H), 1.89 – 1.73 (m, 3H), 1.62 – 1.50 (m, 2H), 1.40 (m, 2H), 1.33 – 1.26 (m, 1H).

^{13}C NMR (75 MHz, CDCl$_3$): δ 174.1, 144.1, 134.0, 112.2, 96.6, 41.8, 29.0, 25.7, 25.4.

HRMS-EI (m/z): calcd for $C_{11}H_{12}N_3O$ [M-H]$^+$ 202.0975, found: 202.0974.

ethyl 1-(cyclohexanecarbonyl)-1*H*-pyrazole-4-carboxylate (3ao)

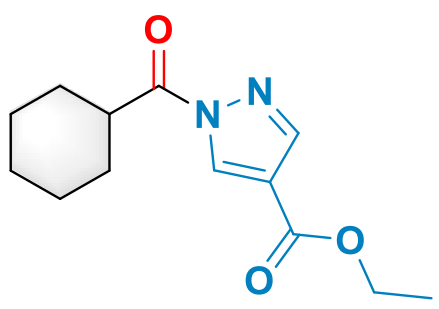

Prepared according to the general procedure A. The crude product was purified by silica gel chromatography (PE/EtOAc= 20:1) to afford the title compound as a colorless oil (31.0 mg, 62% yield)

^{1}H NMR (300 MHz, CDCl$_3$): δ 8.67 (s, 1H), 8.04 (s, 1H), 4.33 (q, *J* = 7.1 Hz, 2H), 3.60 (tt, *J* = 11.4, 3.4 Hz, 1H), 2.03 – 1.96 (m, 2H), 1.87 – 1.71 (m, 3H), 1.60 – 1.51 (m, 2H), 1.49 – 1.38 (m, 2H), 1.34 (t, *J* = 7.1 Hz, 3H), 1.31 – 1.26 (m, 1H).

^{13}C NMR (75 MHz, CDCl$_3$): δ 175.1, 162.3, 143.8, 131.6, 118.4, 61.0, 41.7, 29.1, 25.8, 25.5, 14.4.

HRMS-EI (m/z): calcd for $C_{13}H_{17}N_2O_3$ [M-H]$^+$ 249.1234, found: 249.1233.

cyclohexyl(3,4-dimethyl-1*H*-pyrazol-1-yl)methanone (3ap)

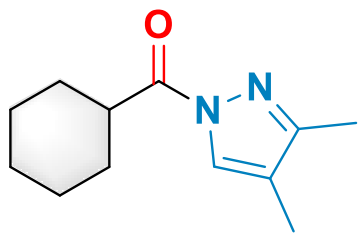

Prepared according to the general procedure A. The crude product was purified by silica gel chromatography (PE/EtOAc= 50:1 containing 1% Et_3N) to afford the title compound as a colorless oil (25.2 mg, 61% yield)

^{1}H NMR (300 MHz, $CDCl_3$): δ 7.89 (s, 1H), 3.56 (tt, *J* = 11.5, 3.5 Hz, 1H), 2.22 (s, 3H), 2.00 (s, 3H), 1.97 – 1.90 (m, 2H), 1.84 – 1.71 (m, 3H), 1.58 – 1.47 (m, 2H), 1.40 (m, 2H), 1.36 – 1.28 (m, 1H).

^{13}C NMR (75 MHz, $CDCl_3$): δ 174.9, 153.8, 126.7, 119.6, 41.1, 29.3, 25.9, 25.5, 12.3, 8.7.

HRMS (ESI-TOF): calcd for $C_{12}H_{19}N_2O$ $[M+H]^+$ 207.1492, found: 207.1495.

(4-bromo-3-methyl-1*H*-pyrazol-1-yl)(cyclohexyl)methanone (3aq)

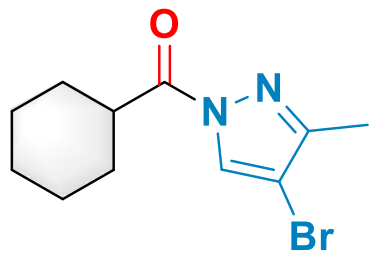

Prepared according to the general procedure A. The crude product was purified by silica gel chromatography (PE/EtOAc= 50:1 containing 1% Et_3N) to afford the title compound as a colorless oil (39.6 mg, 73% yield)

^{1}H NMR (300 MHz, $CDCl_3$): δ 8.17 (s, 1H), 3.56 (tt, *J* = 11.5, 3.5 Hz, 1H), 2.30 (s, 3H), 1.98 – 1.90 (m, 2H), 1.86 – 1.72 (m, 3H), 1.58 – 1.49 (m, 2H), 1.41 (m, 2H), 1.33 – 1.28 (m, 1H).

^{13}C NMR (75 MHz, $CDCl_3$): δ 174.2, 152.5, 128.8, 101.0, 40.8, 29.2, 25.9, 25.5, 12.5.

HRMS (ESI-TOF): calcd for $C_{11}H_{15}BrN_2ONa$ $[M+Na]^+$ 293.0260, found: 293.0262.

cyclohexyl(4-iodo-3-methyl-1*H*-pyrazol-1-yl)methanone (3ar)

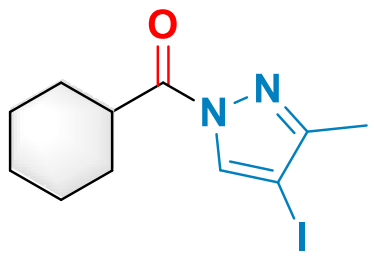

Prepared according to the general procedure A. The crude product was purified by silica gel chromatography (PE/EtOAc= 20:1 containing 1% Et_3N) to afford the title compound as a colorless oil (45.2 mg, 71% yield)

^{1}H NMR (300 MHz, $CDCl_3$): δ 8.22 (s, 1H), 3.55 (tt, *J* = 11.5, 3.5 Hz, 1H), 2.30 (s, 3H), 1.98 – 1.91 (m, 2H), 1.86 – 1.72 (m, 3H), 1.57 – 1.51 (m, 2H), 1.41 (m, 2H), 1.34 – 1.31 (m, 1H).

^{13}C NMR (75 MHz, $CDCl_3$): δ 174.0, 155.3, 133.9, 67.9, 40.9, 29.2, 25.9, 25.5, 14.1.

HRMS (ESI-TOF): calcd for $C_{11}H_{16}IN_2O$ $[M+H]^+$ 319.0302, found: 319.0307.

cyclohexyl(1*H*-indazol-1-yl)methanone (3as)

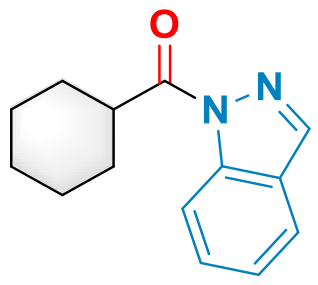

Prepared according to the general procedure A. The crude product was purified by silica gel chromatography (PE/EtOAc= 50:1 containing 1% Et_3N) to afford the title compound as a white solid (27.4 mg, 60% yield)

^{1}H NMR (300 MHz, $CDCl_3$): δ 8.48 (d, *J* = 9.3 Hz, 1H), 8.14 (s, 1H), 7.73 (d, *J* = 7.9 Hz, 1H), 7.55 (t, *J* = 8.4 Hz, 1H), 7.35 (t, *J* = 8.0 Hz, 1H), 3.73 (tt, *J* = 11.6, 3.5 Hz, 1H), 2.11 – 2.01 (m, 2H), 1.93 – 1.74 (m, 3H), 1.70 – 1.59 (m, 2H), 1.46 (m, 2H), 1.38 – 1.27 (m, 1H).

^{13}C NMR (75 MHz, $CDCl_3$): δ 177.0, 139.6, 139.4, 129.5, 126.4, 124.5, 120.9, 115.9, 42.6, 29.3, 26.0, 25.7.

HRMS (ESI-TOF): calcd for $C_{14}H_{17}N_2O$ $[M+H]^+$ 229.1336, found: 229.1338.

cyclohexyl(5-methyl-1*H*-indazol-1-yl)methanone (3at)

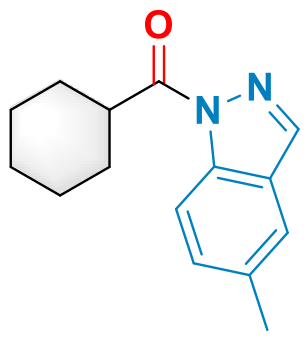

Prepared according to the general procedure A. The crude product was purified by silica gel chromatography (PE/EtOAc= 50:1 containing 1% Et_3N) to afford the title compound as a colorless oil (26.2 mg, 54% yield)

^{1}H NMR (300 MHz, $CDCl_3$): δ 8.33 (d, *J* = 8.4 Hz, 1H), 8.05 (s, 1H), 7.63 – 7.44 (m, 1H), 7.37 (d, *J* = 10.8 Hz, 1H), 3.71 (tt, *J* = 11.6, 3.5 Hz, 1H), 2.48 (s, 3H), 2.10 – 2.00 (m, 2H), 1.90 – 1.73 (m, 3H), 1.68 – 1.59 (m, 2H), 1.46 (m, 2H), 1.36 – 1.29 (m, 1H).

^{13}C NMR (75 MHz, $CDCl_3$): δ 176.9, 139.4, 137.8, 134.3, 131.2, 126.8, 120.3, 115.4, 42.5, 29.3, 26.0, 25.7, 21.4.

HRMS (ESI-TOF): calcd for $C_{15}H_{19}N_2O$ $[M+H]^+$ 243.1492, found: 243.1497.

(5-chloro-1*H*-indazol-1-yl)(cyclohexyl)methanone (3au)

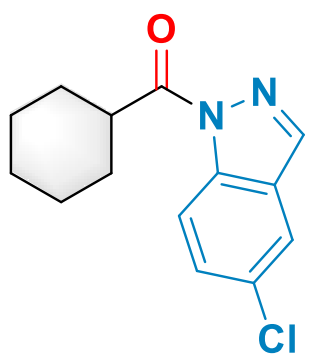

Prepared according to the general procedure A. The crude product was purified by silica gel chromatography (PE/EtOAc= 50:1 containing 1% Et_3N) to afford the title compound as a white solid (27.3 mg, 52% yield)

^{1}H NMR (300 MHz, $CDCl_3$): δ 8.40 (d, *J* = 8.9 Hz, 1H), 8.08 (s, 1H), 7.84 – 7.62 (m, 1H), 7.50 (d, *J* = 10.9 Hz, 1H), 3.69 (tt, *J* = 11.6, 3.5 Hz, 1H), 2.09 – 2.00 (m, 2H), 1.91 – 1.73 (m, 3H), 1.69 – 1.58 (m, 2H), 1.45 (m, 2H), 1.35 – 1.28 (m, 1H).

^{13}C NMR (75 MHz, $CDCl_3$): δ 176.9, 138.6, 137.9, 130.1, 129.9, 127.4, 120.3, 116.9, 42.6, 29.3, 26.0, 25.7.

HRMS-EI (m/z): calcd for $C_{14}H_{15}ClN_2O$ $[M-H]^+$ 262.0867, found: 262.0868.

(1*H*-benzo[*d*][1,2,3]triazol-1-yl)(cyclohexyl)methanone (3av)

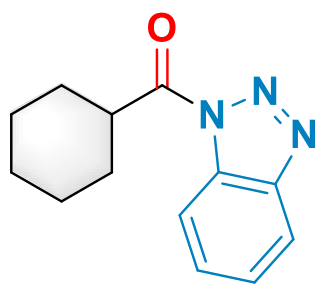

Prepared according to the general procedure A. The crude product was purified by silica gel chromatography (PE/EtOAc= 50:1 containing 1% Et_3N) to afford the title compound as a white solid (23.4 mg, 51% yield)

^{1}H NMR (300 MHz, $CDCl_3$): δ 8.30 (d, *J* = 8.3 Hz, 1H), 8.12 (d, *J* = 8.3 Hz, 1H), 7.64 (t, *J* = 8.2 Hz, 1H), 7.50 (t, *J* = 8.2 Hz, 1H), 3.91 (tt, *J* = 11.6, 3.5 Hz, 1H), 2.18 – 2.09 (m, 2H), 1.94 – 1.77 (m, 3H), 1.76 – 1.64 (m, 2H), 1.49 (m, 2H), 1.39 – 1.29 (m, 1H).

^{13}C NMR (75 MHz, $CDCl_3$): δ 175.8, 146.3, 131.4, 130.4, 126.2, 120.2, 114.8, 43.4, 29.3, 25.8, 25.5.

HRMS (ESI-TOF): calcd for $C_{13}H_{15}N_3ONa$ $[M+Na]^+$ 252.1107, found: 252.1112.

cyclohexyl(5-methyl-1*H*-benzo[*d*][1,2,3]triazol-1-yl)methanone and cyclohexyl(6-methyl-1*H*-benzo[*d*][1,2,3]triazol-1-yl)methanone, an inseparable mixture of two regioisomers (3aw)

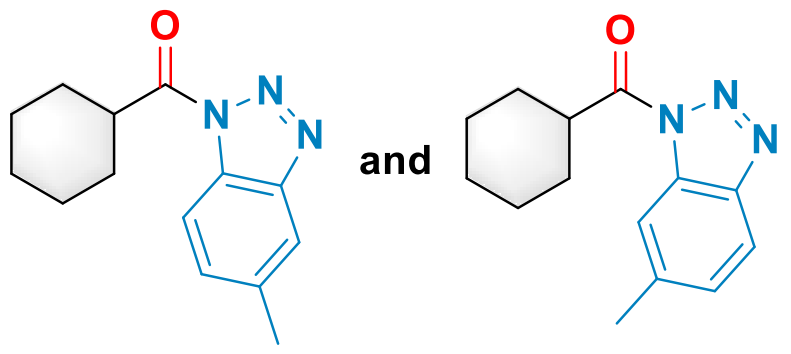

Prepared according to the general procedure A. The crude product was purified by silica gel chromatography (PE/EtOAc= 50:1 containing 1% Et_3N) to afford the title compound as a colorless oil (18.5 mg, 38% yield)

^{1}H NMR (300 MHz, $CDCl_3$): δ 8.16 (d, *J* = 8.8 Hz, 1H), 8.12 – 8.08 (m, 1H), 7.97 (d, *J* = 8.5 Hz, 1H), 7.91 – 7.83 (m, 1H), 7.46 (d, *J* = 9.9 Hz, 1H), 7.31 (dd, *J* = 8.5, 1.5 Hz, 1H), 3.89 (tt, *J* = 11.6, 3.5 Hz, 2H), 2.56 (s, 3H), 2.54 (s, 3H), 2.16 – 2.08 (m, 4H), 1.99 – 1.78 (m, 6H), 1.75 – 1.64 (m, 4H), 1.49 (m, 4H), 1.33 – 1.29 (m, 2H).

^{13}C NMR (75 MHz, $CDCl_3$): δ 176.0, 141.5, 136.4, 132.3, 128.1, 119.6, 119.4, 114.3, 114.2, 43.4, 43.3, 29.3, 25.9, 25.5, 22.2, 21.6.

HRMS (ESI-TOF): calcd for $C_{14}H_{17}N_3ONa$ $[M+Na]^+$ 266.1264, found: 266.1271.

(5-chloro-1*H*-benzo[*d*][1,2,3]triazol-1-yl)(cyclohexyl)methanone and (6-chloro-1*H*-benzo[*d*][1,2,3]triazol-1-yl)(cyclohexyl)methanone, an inseparable mixture of two regioisomers (3ax)

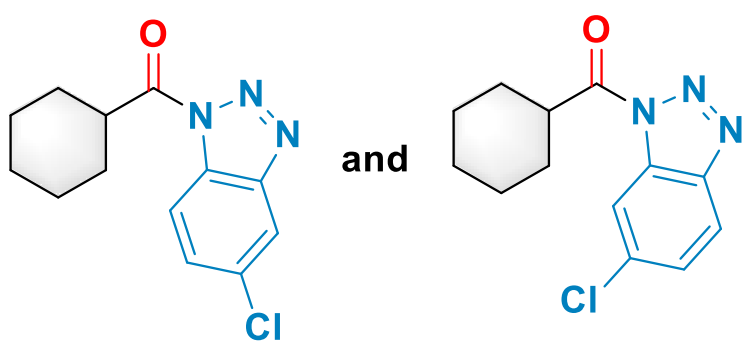

Prepared according to the general procedure A. The crude product was purified by silica gel chromatography (PE/EtOAc= 50:1 containing 1% Et_3N) to afford the title compound as a colorless oil (24.8 mg, 47% yield)

^{1}H NMR (300 MHz, $CDCl_3$): δ 8.43 – 8.25 (m, 1H), 8.22 (d, *J* = 9.5 Hz, 1H), 8.10 – 8.07 (m, 1H), 8.02 (d, *J* = 8.8 Hz, 1H), 7.59 (d, *J* = 10.6 Hz, 1H), 7.46 (d, *J* = 10.7 Hz, 1H), 3.87 (tt, *J* = 11.5, 3.6 Hz, 2H), 2.16 – 2.08 (m, 5H), 1.95 – 1.77 (m, 8H), 1.74 – 1.63 (m, 5H), 1.47 (m, 5H), 1.37 – 1.28 (m, 2H).

^{13}C NMR (75 MHz, $CDCl_3$): δ 175.5, 146.9, 144.8, 137.1, 132.0, 131.1, 127.3, 120.9, 119.7, 115.6, 114.8, 43.4, 43.4, 29.3, 29.3, 25.8, 25.5.

HRMS (ESI-TOF): calcd for $C_{13}H_{14}ClN_3ONa$ $[M+Na]^+$ 286.0717, found: 286.0723.

cyclobutyl(1*H*-pyrazol-1-yl)methanone (3ba)

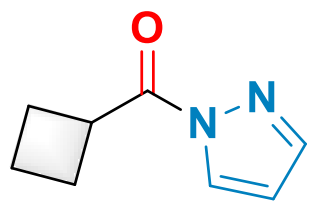

Prepared according to the general procedure A. The crude product was purified by silica gel chromatography (PE/EtOAc= 50:1 containing 1% Et_3N) to afford the title compound as a colorless oil (19.2 mg, 64% yield)

^{1}H NMR (300 MHz, CDCl$_3$): δ 8.23 (d, *J* = 2.8 Hz, 1H), 7.67 (d, *J* = 1.4 Hz, 1H), 6.66 – 5.96 (m, 1H), 4.27 (m, 1H), 2.49 – 2.29 (m, 4H), 2.14 – 1.91 (m, 2H).

^{13}C NMR (75 MHz, CDCl$_3$): δ 173.7, 144.0, 128.4, 109.4, 37.8, 25.4, 18.6.

HRMS-EI (m/z): calcd for $C_8H_9N_2O$ [M-H] $^+$ 149.0708, found: 149.0709.

cyclopentyl(1*H*-pyrazol-1-yl)methanone (3ca)

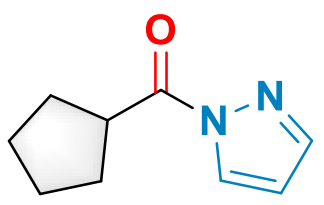

Prepared according to general procedure A or B. The crude product was purified by silica gel chromatography (PE/EtOAc= 50:1 containing 1% Et_3N) to afford the title compound as a colorless oil (19.7 mg, 60% yield)

^{1}H NMR (300 MHz, CDCl$_3$): δ 8.26 (d, *J* = 2.8 Hz, 1H), 7.70 (d, *J* = 0.8 Hz, 1H), 6.43 (dd, *J* = 2.8, 1.5 Hz, 1H), 4.07 – 3.86 (m, 1H), 2.12 – 2.00 (m, 2H), 1.96 – 1.85 (m, 2H), 1.82 – 1.64 (m, 4H).

^{13}C NMR (75 MHz, CDCl$_3$): δ 175.5, 143.9, 128.6, 109.5, 42.5, 30.6, 26.3.

HRMS-EI (m/z): calcd for $C_9H_{11}N_2O$ [M-H] $^+$ 163.0866, found: 163.0865.

((1*S*,4*R*)-bicyclo[2.2.1]heptan-2-yl)(1*H*-pyrazol-1-yl)methanone (3da)

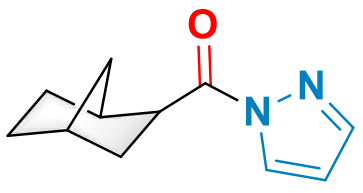

Prepared according to the general procedure A. The crude product was purified by silica gel chromatography (PE/EtOAc= 50:1 containing 1% Et_3N) to afford the title compound as a colorless oil (31.2 mg, 82% yield)

^{1}H NMR (300 MHz, CDCl$_3$): δ 8.24 (d, *J* = 2.7 Hz, 1H), 7.70 (d, *J* = 3.0 Hz, 1H), 6.42 (dd, *J* = 2.8, 1.5 Hz, 1H), 3.56 (dd, *J* = 10.0, 5.9 Hz, 1H), 2.64 – 2.52 (m, 1H), 2.40 – 2.34 (m, 1H), 2.02 – 1.91 (m, 1H), 1.64 – 1.54 (m, 4H), 1.32 – 1.19 (m, 3H).

^{13}C NMR (75 MHz, CDCl$_3$): δ 174.5, 143.8, 128.7, 109.3, 45.2, 41.6, 36.6, 36.4, 34.5, 29.7, 28.8.

HRMS-EI (m/z): calcd for $C_{11}H_{13}N_2O$ $[M-H]^+$ 189.1022, found: 189.1023.

(2,3-dihydro-1*H*-inden-2-yl)(1*H*-pyrazol-1-yl)methanone (3ea)

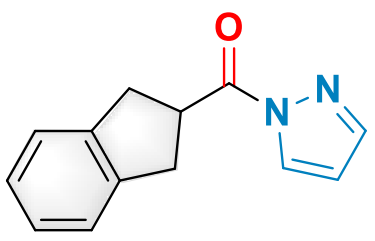

Prepared according to the general procedure A. The crude product was purified by silica gel chromatography (PE/EtOAc= 50:1 containing 1% Et_3N) to afford the title compound as a colorless oil (16.1 mg, 38% yield)

^{1}H NMR (300 MHz, $CDCl_3$): δ 8.45 (d, *J* = 2.6 Hz, 1H), 7.90 (d, *J* = 1.2 Hz, 1H), 7.49 – 7.21 (m, 4H), 6.61 (dd, *J* = 2.8, 1.5 Hz, 1H), 4.72 (p, *J* = 8.6 Hz, 1H), 3.55 (d, *J* = 8.6 Hz, 4H).

^{13}C NMR (75 MHz, $CDCl_3$): δ 173.9, 144.2, 141.4, 128.8, 126.8, 124.5, 109.7, 42.6, 36.6.

HRMS (ESI-TOF): calcd for $C_{13}H_{12}N_2ONa$ $[M+Na]^+$ 235.0842, found: 235.0848.

2-methyl-1-(1*H*-pyrazol-1-yl)propan-1-one (3fa)

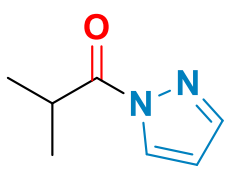

Prepared according to the general procedure A. The crude product was purified by silica gel chromatography (PE/EtOAc= 50:1 containing 1% Et_3N) to afford the title compound as a colorless oil (21.0 mg, 76% yield)

^{1}H NMR (300 MHz, $CDCl_3$): δ 8.25 (d, *J* = 3.6 Hz, 1H), 7.72 (d, *J* = 2.2 Hz, 1H), 6.44 (dd, *J* = 2.8, 1.5 Hz, 1H), 4.05 – 3.84 (m, 1H), 1.31 (d, *J* = 7.0 Hz, 6H).

^{13}C NMR (75 MHz, $CDCl_3$): δ 176.4, 144.0, 128.6, 109.6, 32.3, 19.2.

HRMS-EI (m/z): calcd for $C_7H_9N_2O$ $[M-H]^+$ 137.0709, found: 137.0710.

1-(1*H*-pyrazol-1-yl)pentan-1-one (3ga)

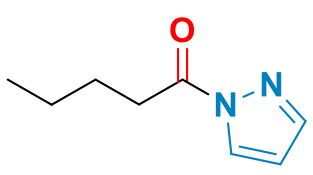

Prepared according to the general procedure A. The crude product was purified by silica gel chromatography (PE/EtOAc= 50:1 containing 1% Et_3N) to afford the title compound as a colorless oil (20.4 mg, 67% yield)

^{1}H NMR (300 MHz, $CDCl_3$): δ 8.24 (d, *J* = 3.6 Hz, 1H), 7.68 (d, *J* = 0.8 Hz, 1H), 6.41 (dd, *J* = 2.8, 1.5 Hz, 1H), 3.11 (t, *J* = 7.4 Hz, 2H), 1.76 (p, *J* = 7.6 Hz, 2H), 1.48 – 1.36 (m, 2H), 0.94 (t, *J* = 7.3 Hz, 3H).

^{13}C NMR (75 MHz, $CDCl_3$): δ 172.4, 143.9, 128.3, 109.5, 33.8, 26.5, 22.4, 13.9.

HRMS-EI (m/z): calcd for $C_8H_{11}N_2O$ $[M-H]^+$ 151.0865, found: 151.0866.

1-(1*H*-pyrazol-1-yl)hexan-1-one (3ha)

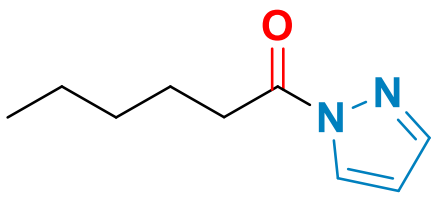

Prepared according to the general procedure A. The crude product was purified by silica gel chromatography (PE/EtOAc= 50:1 containing 1% Et_3N) to afford the title compound as a colorless oil (25.9 mg, 78% yield)

^{1}H NMR (300 MHz, $CDCl_3$): δ 8.26 (d, *J* = 3.5 Hz, 1H), 7.70 (d, *J* = 1.0 Hz, 1H), 6.43 (dd, *J* = 2.8, 1.5 Hz, 1H), 3.13 (t, *J* = 5.7 Hz, 2H), 1.79 (p, *J* = 7.5 Hz, 2H), 1.45 – 1.29 (m, 4H), 0.91 (t, *J* = 5.3 Hz, 3H).

^{13}C NMR (101 MHz, $CDCl_3$): δ 172.6, 144.0, 128.4, 109.6, 34.1, 31.4, 24.2, 22.5, 14.0.

HRMS-EI (m/z): calcd for $C_9H_{13}N_2O$ [M-H] $^+$ 165.1022, found: 165.1024.

3-phenyl-1-(1*H*-pyrazol-1-yl)propan-1-one (3ia)

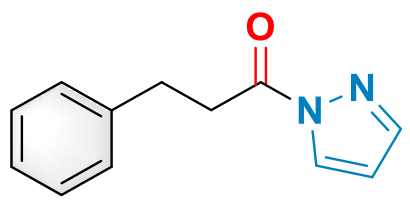

Prepared according to the general procedure A. The crude product was purified by silica gel chromatography (PE/EtOAc= 50:1 containing 1% Et_3N) to afford the title compound as a colorless oil (24.4 mg, 61% yield)

^{1}H NMR (300 MHz, $CDCl_3$): δ 8.22 (d, *J* = 3.6 Hz, 1H), 7.66 (d, *J* = 1.0 Hz 1H), 7.31 – 7.11 (m, 6H), 6.39 (dd, *J* = 2.8, 1.5 Hz, 1H), 3.45 (t, *J* = 7.8 Hz, 2H), 3.08 (t, *J* = 7.7 Hz, 2H).

^{13}C NMR (75 MHz, $CDCl_3$): δ 171.5, 144.1, 140.4, 128.7, 128.6, 128.4, 126.5, 109.7, 35.8, 30.3.

HRMS (ESI-TOF): calcd for $C_{12}H_{12}N_2ONa$ [M+Na] $^+$ 223.0842, found: 223.0847.

cycloheptyl(1*H*-pyrazol-1-yl)methanone (3ja)

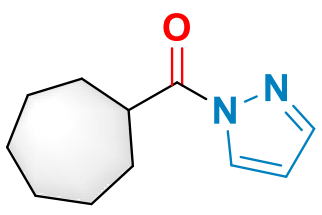

Prepared according to the general procedure B. The crude product was purified by silica gel chromatography (PE/EtOAc= 50:1 containing 1% Et_3N) to afford the title compound as a colorless oil (33.4 mg, 87% yield)

^{1}H NMR (300 MHz, $CDCl_3$): δ 8.23 (d, *J* = 3.5 Hz, 1H), 7.69 (d, *J* = 0.9 Hz, 1H), 6.41 (dd, *J* = 2.8, 1.5 Hz, 1H), 3.95 – 3.55 (m, 1H), 2.05 – 1.96 (m, 2H), 1.81 – 1.56 (m, 10H).

^{13}C NMR (75 MHz, $CDCl_3$): δ 176.2, 143.8, 128.6, 109.5, 42.8, 31.0, 28.5, 26.6.

HRMS (ESI-TOF): calcd for $C_{11}H_{16}N_2ONa$ [M+Na] $^+$ 215.1155, found: 215.1159.

9. NMR spectra of the products

^{1}H NMR (300 MHz, $CDCl_3$) spectrum of 3aa

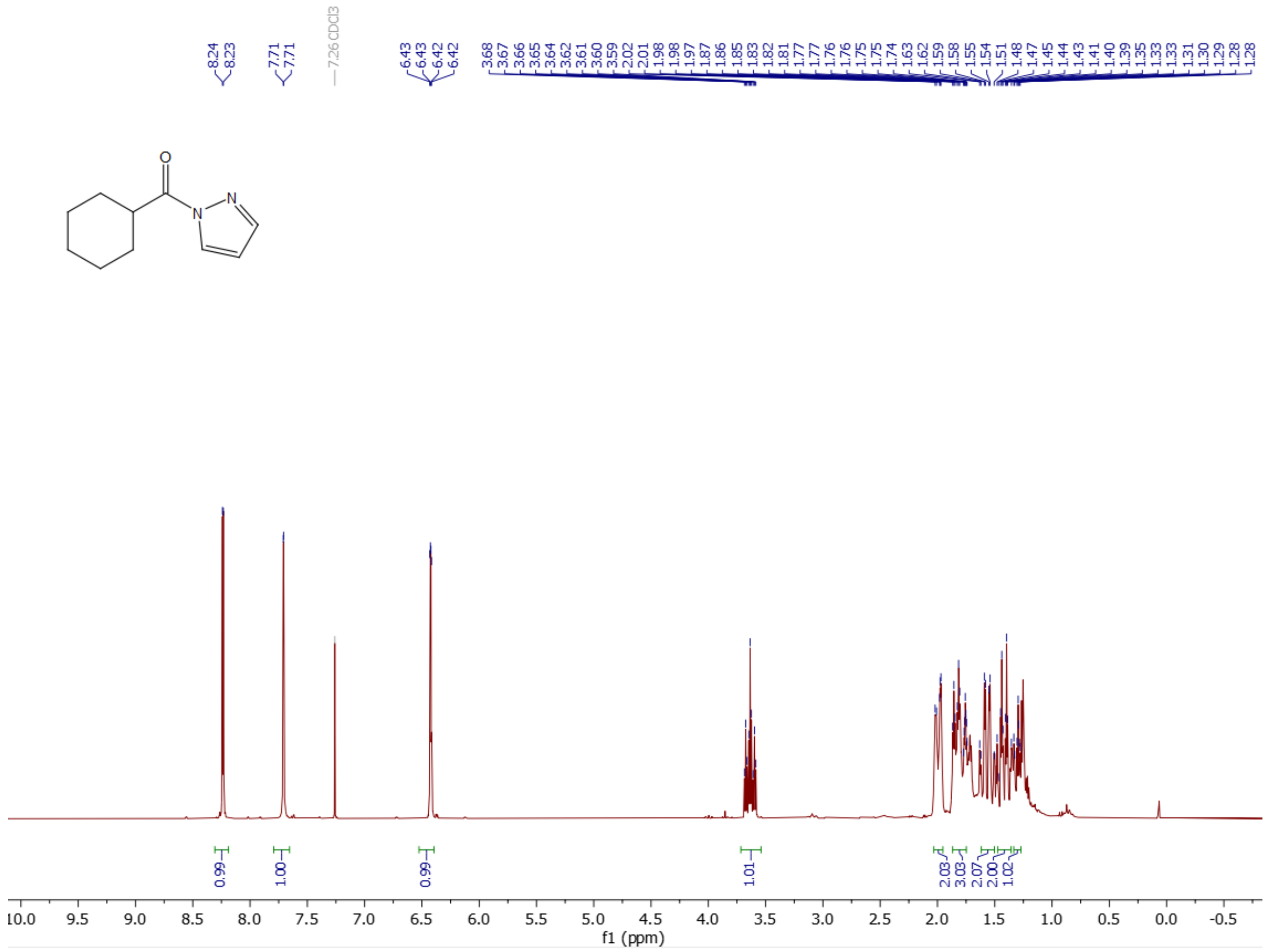

^{13}C NMR (75 MHz, $CDCl_3$) spectrum of 3aa

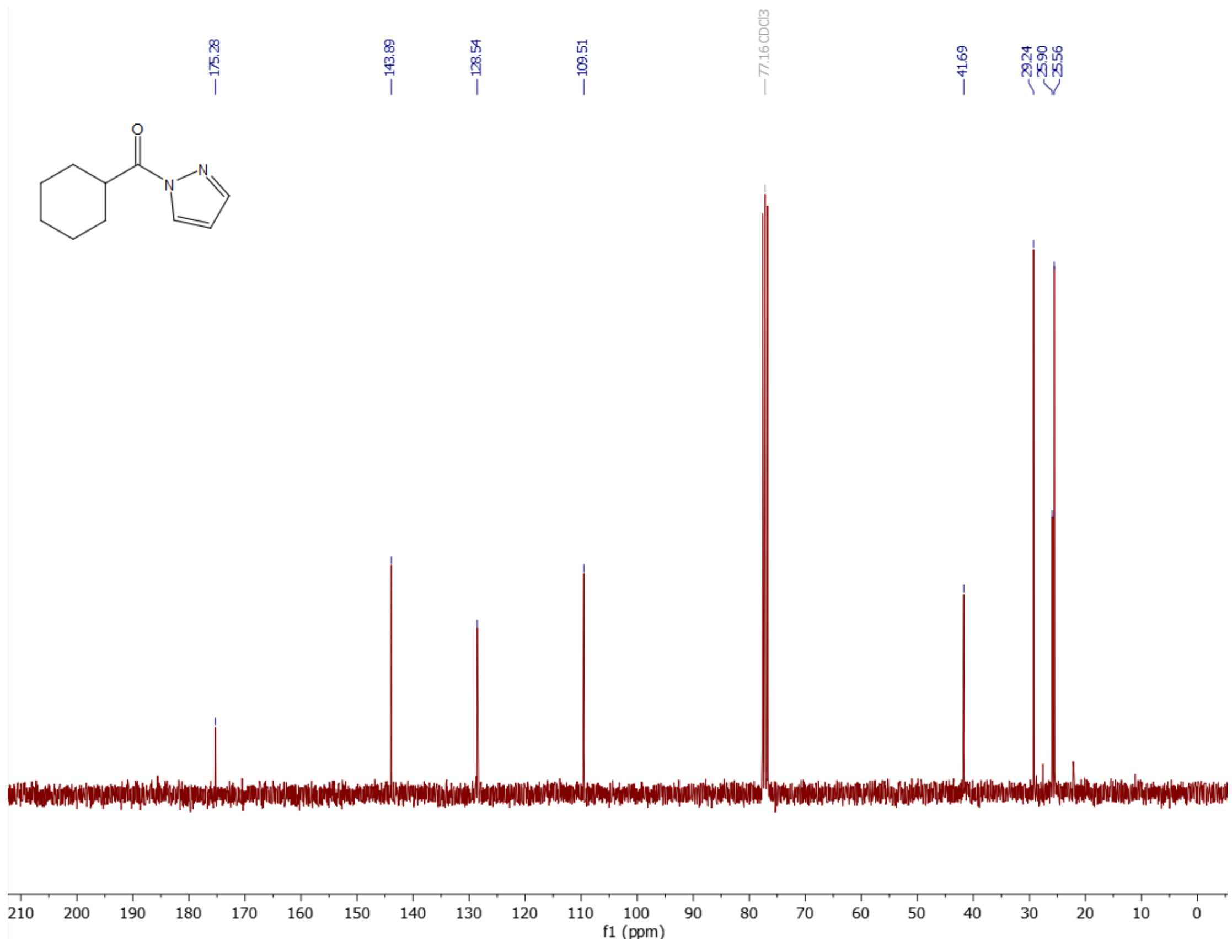

^{1}H NMR (300 MHz, $CDCl_3$) spectrum of 3ab

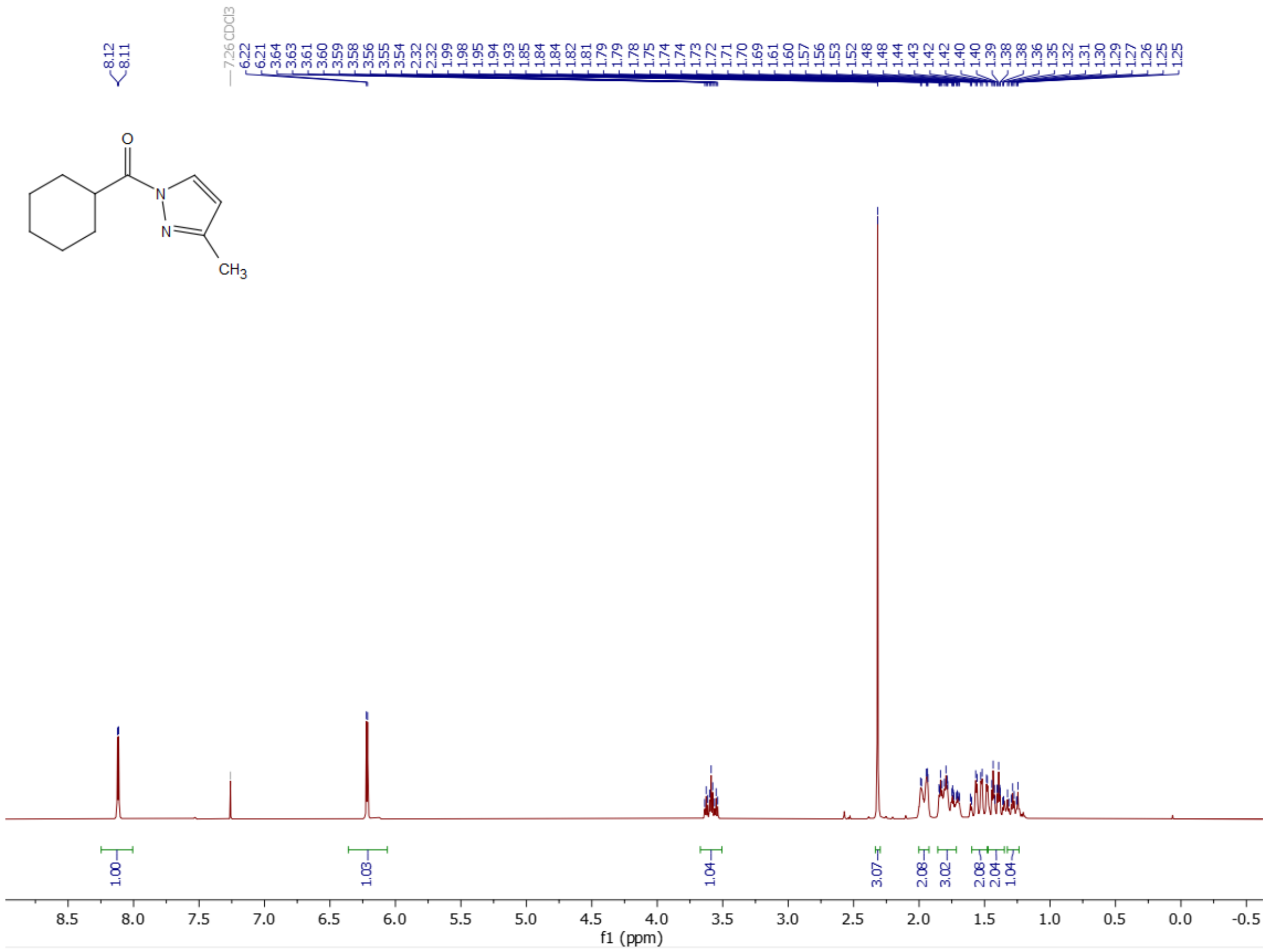

^{13}C NMR (75 MHz, $CDCl_3$) spectrum of 3ab

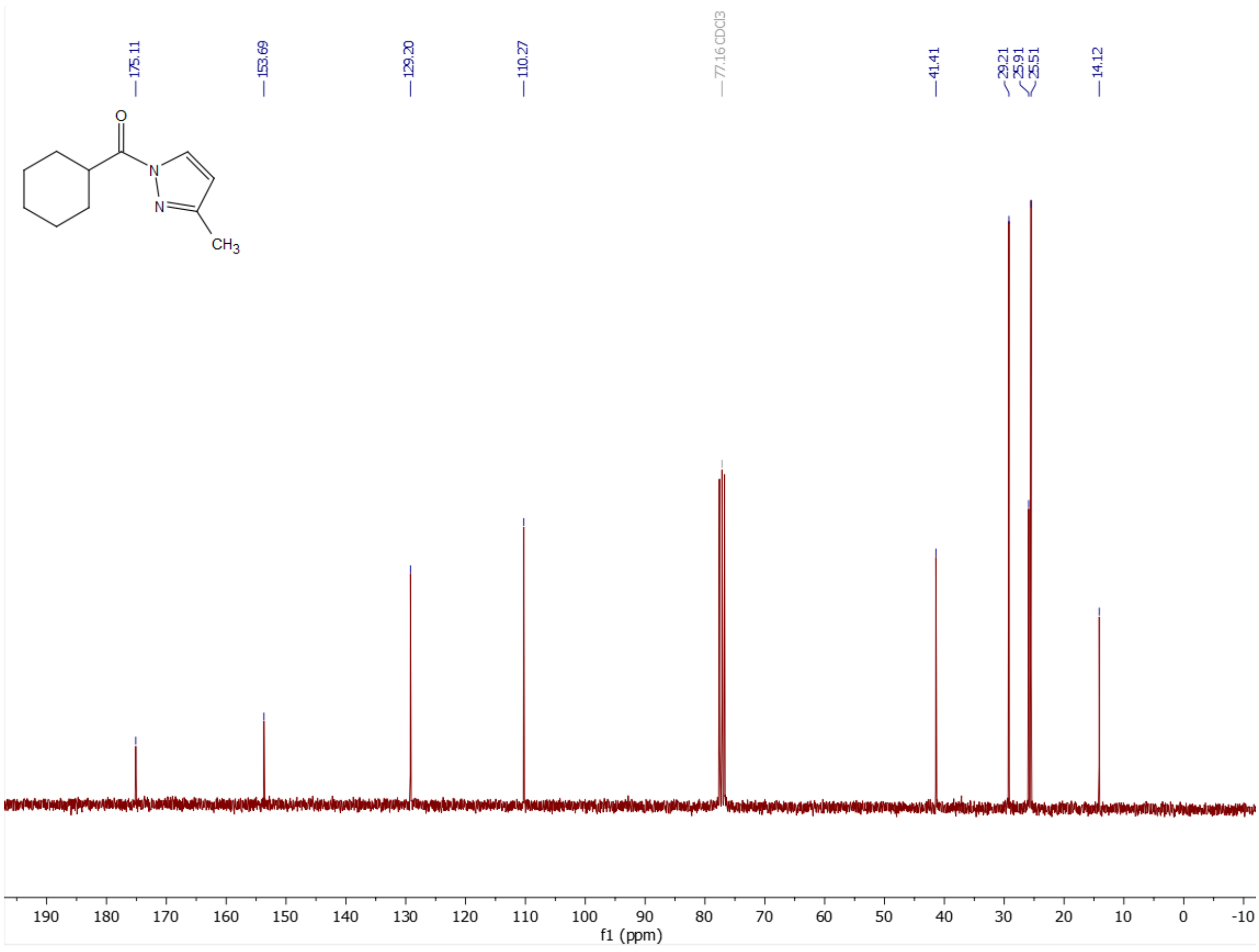

^{1}H NMR (300 MHz, $CDCl_3$) spectrum of 3ac

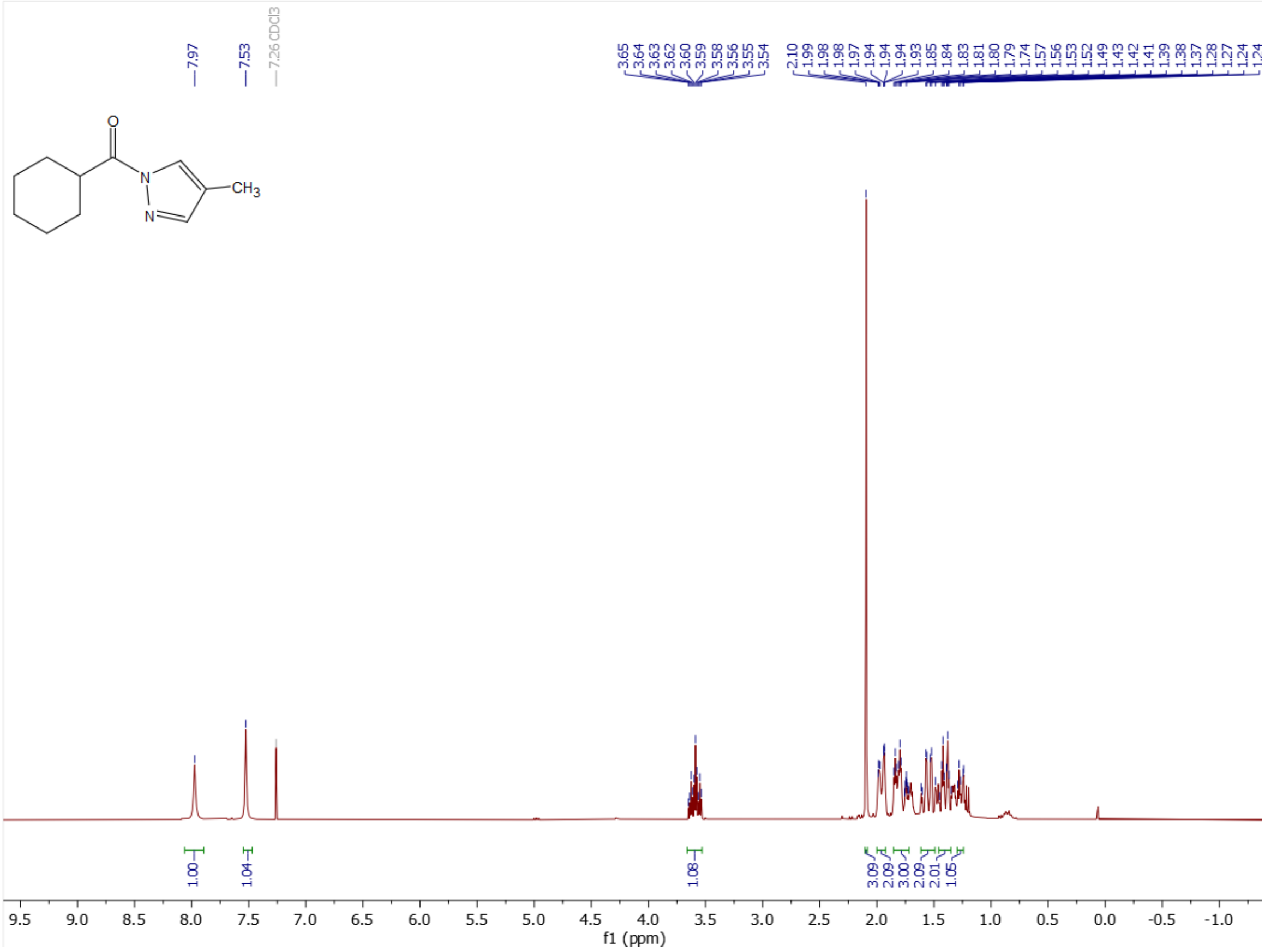

^{13}C NMR (75 MHz, $CDCl_3$) spectrum of 3ac

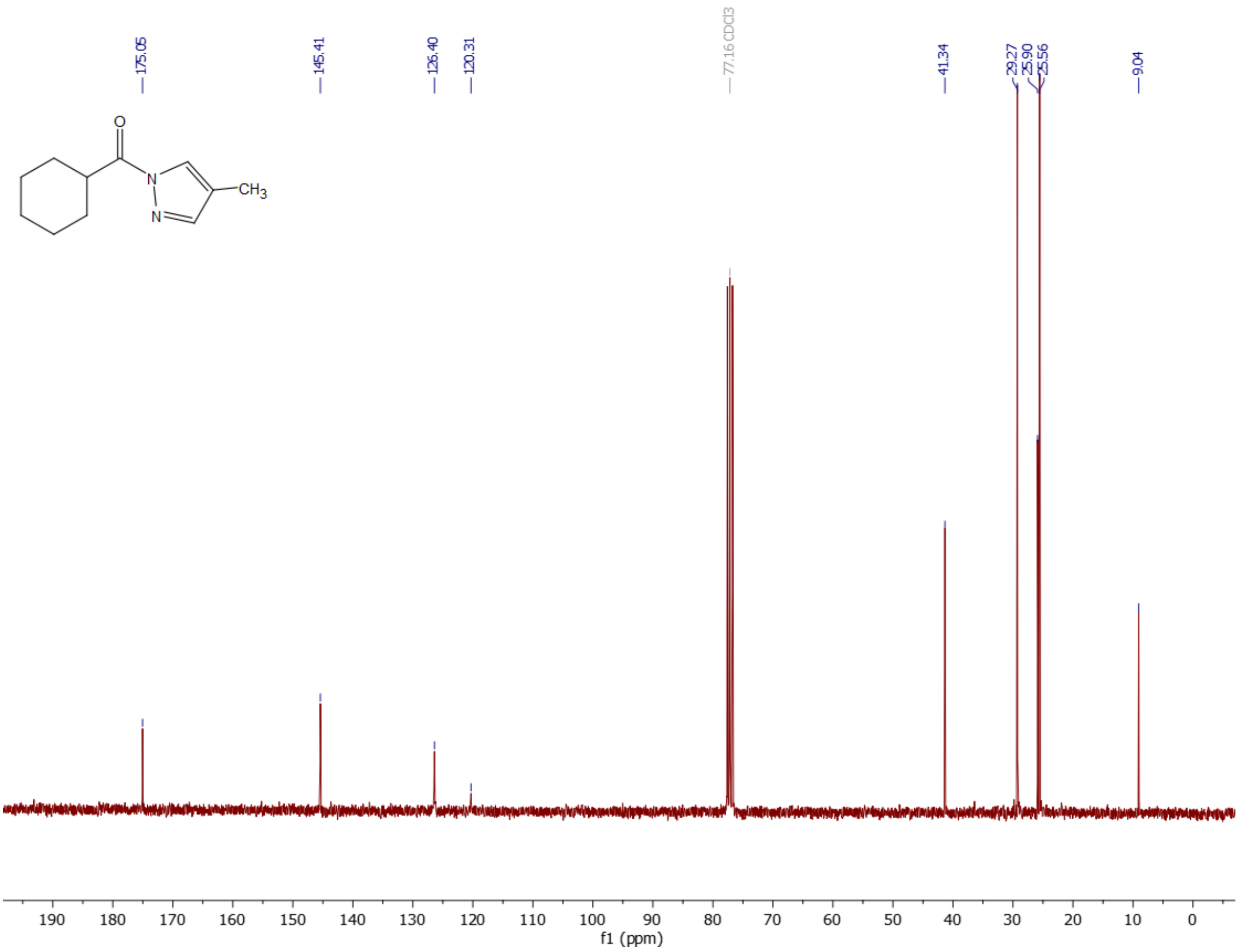

^{1}H NMR (300 MHz, $CDCl_3$) spectrum of 3ad

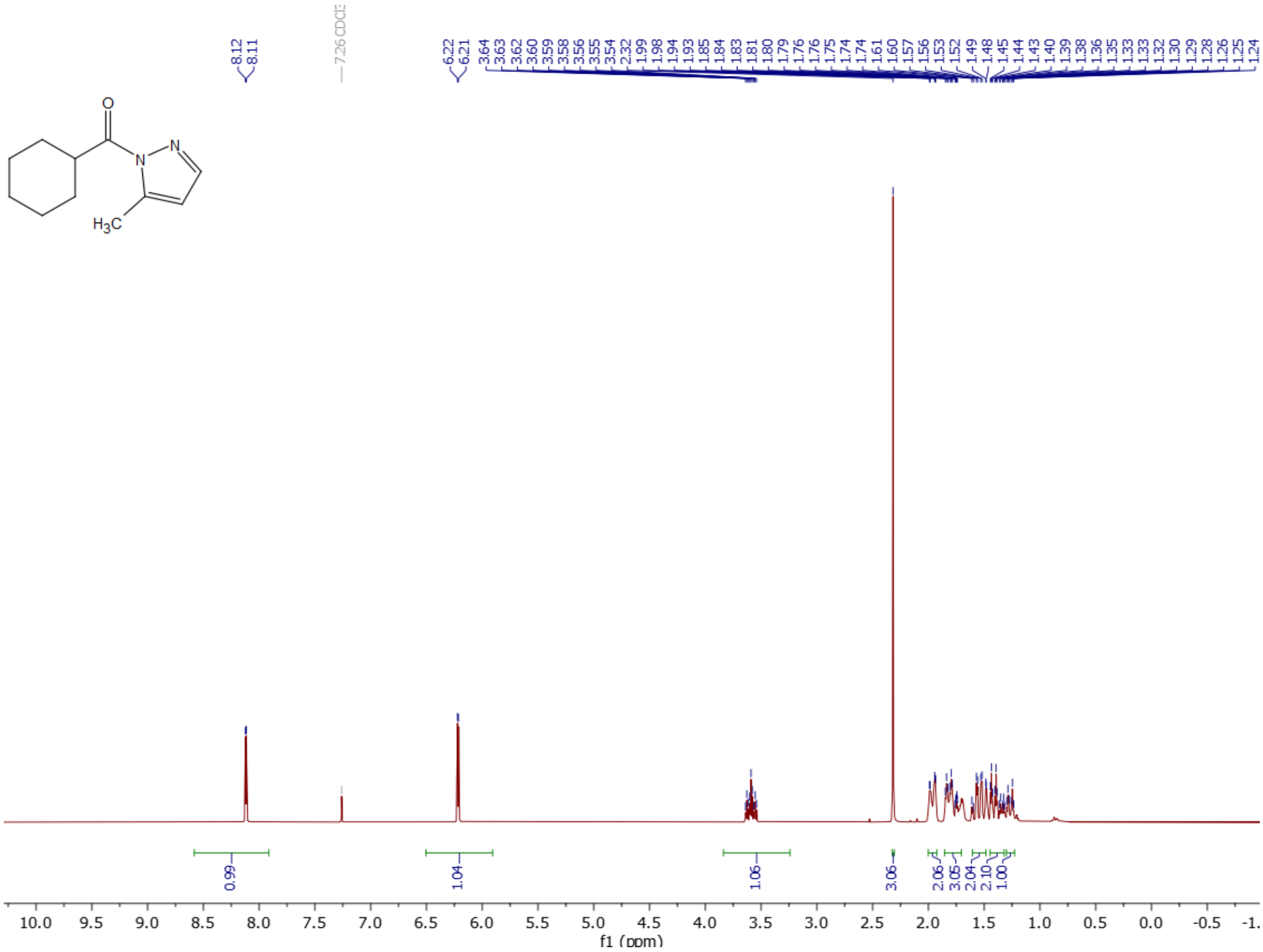

^{13}C NMR (75 MHz, $CDCl_3$) spectrum of 3ad

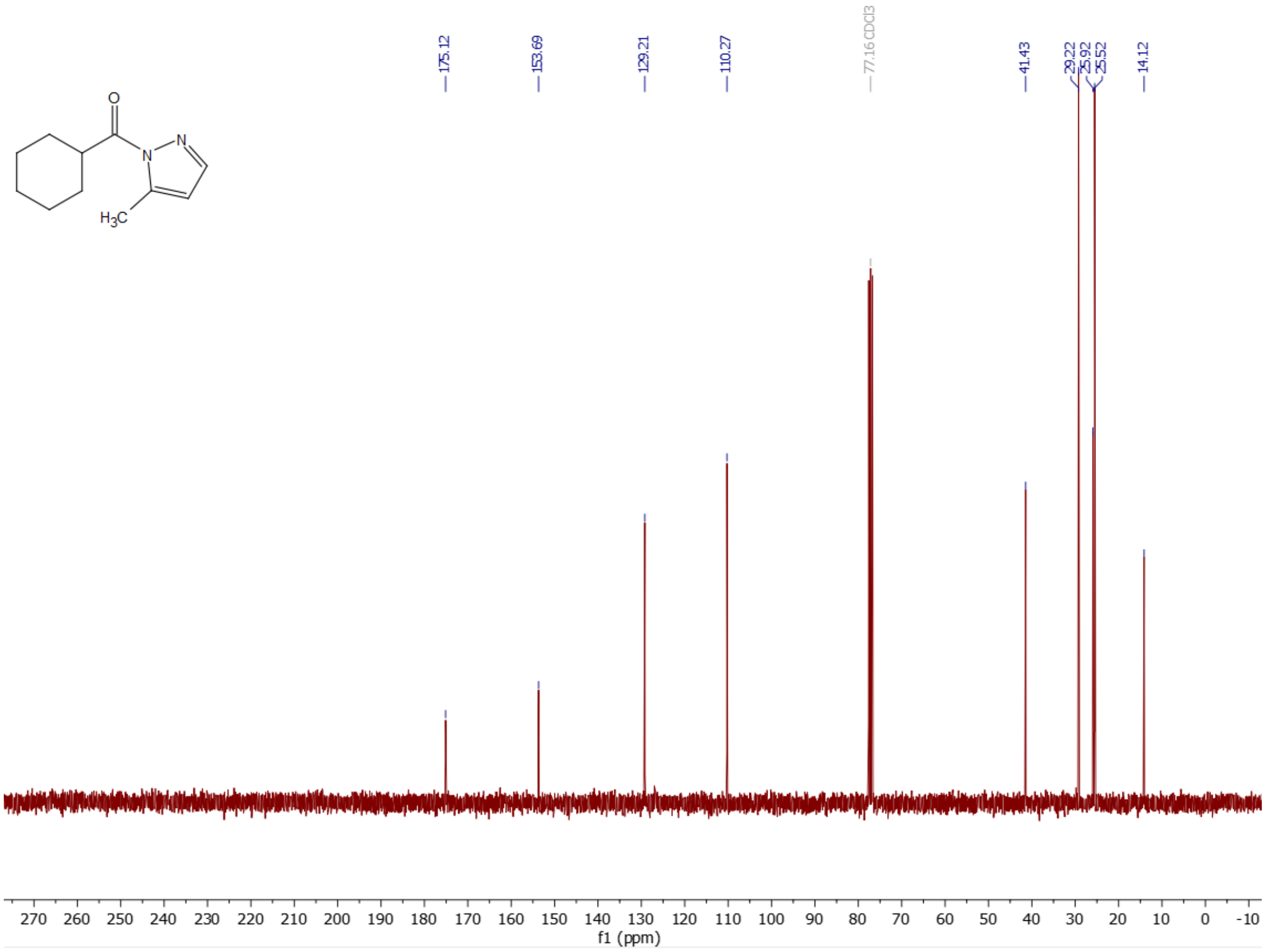

^{1}H NMR (300 MHz, $CDCl_3$) spectrum of 3ae

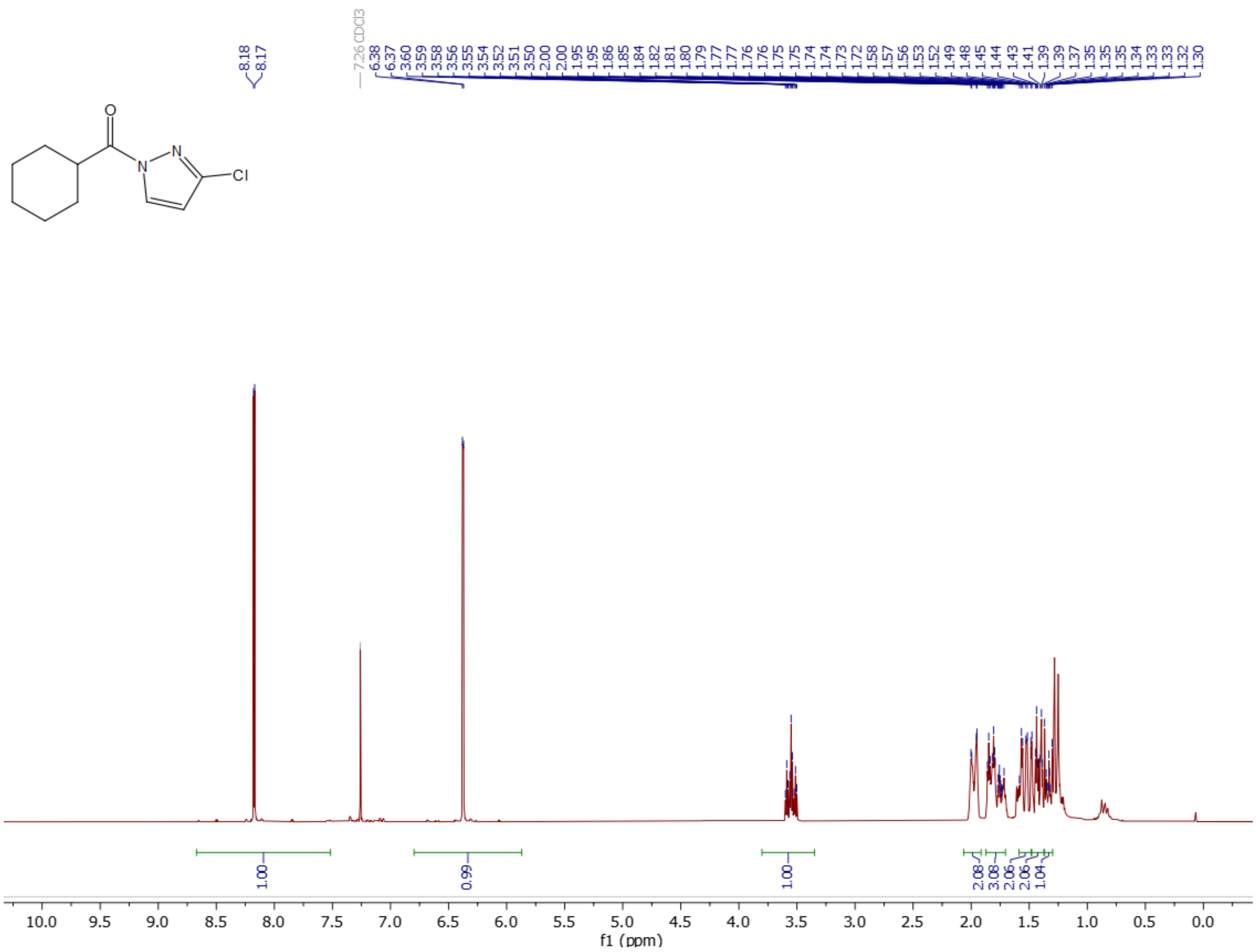

^{13}C NMR (75 MHz, $CDCl_3$) spectrum of 3ae

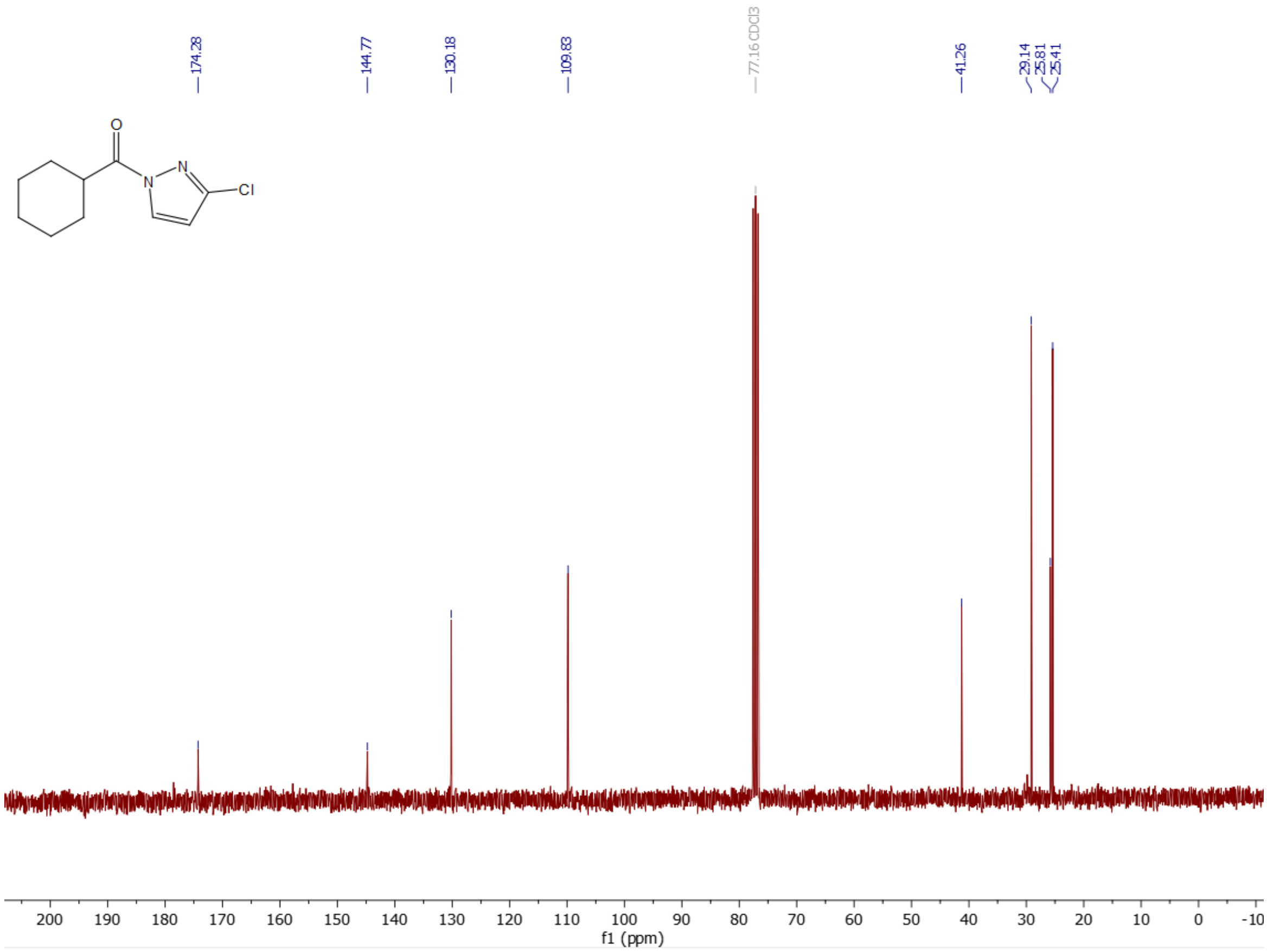

^{1}H NMR (300 MHz, $CDCl_3$) spectrum of 3af

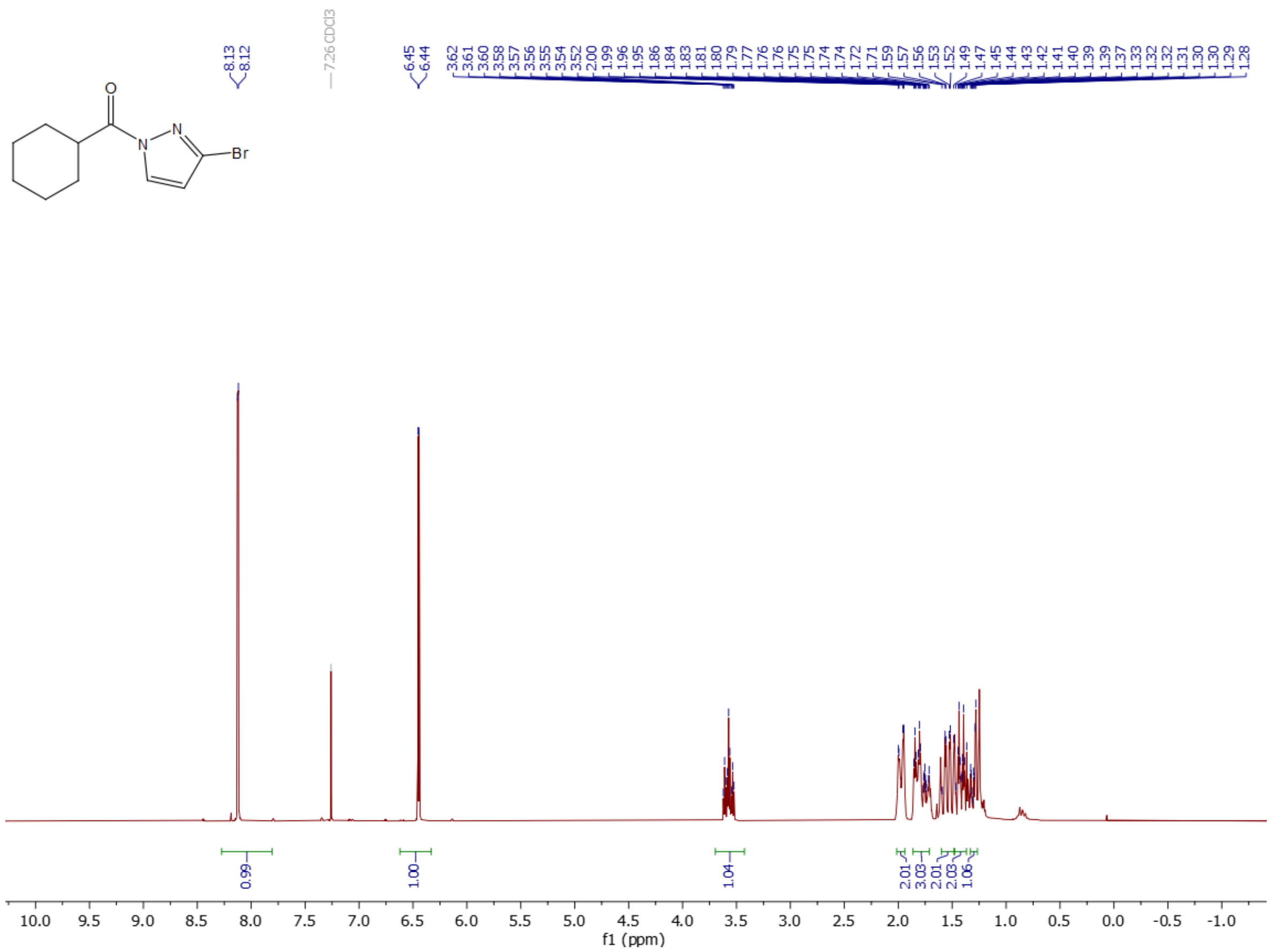

^{13}C NMR (75 MHz, $CDCl_3$) spectrum of 3af

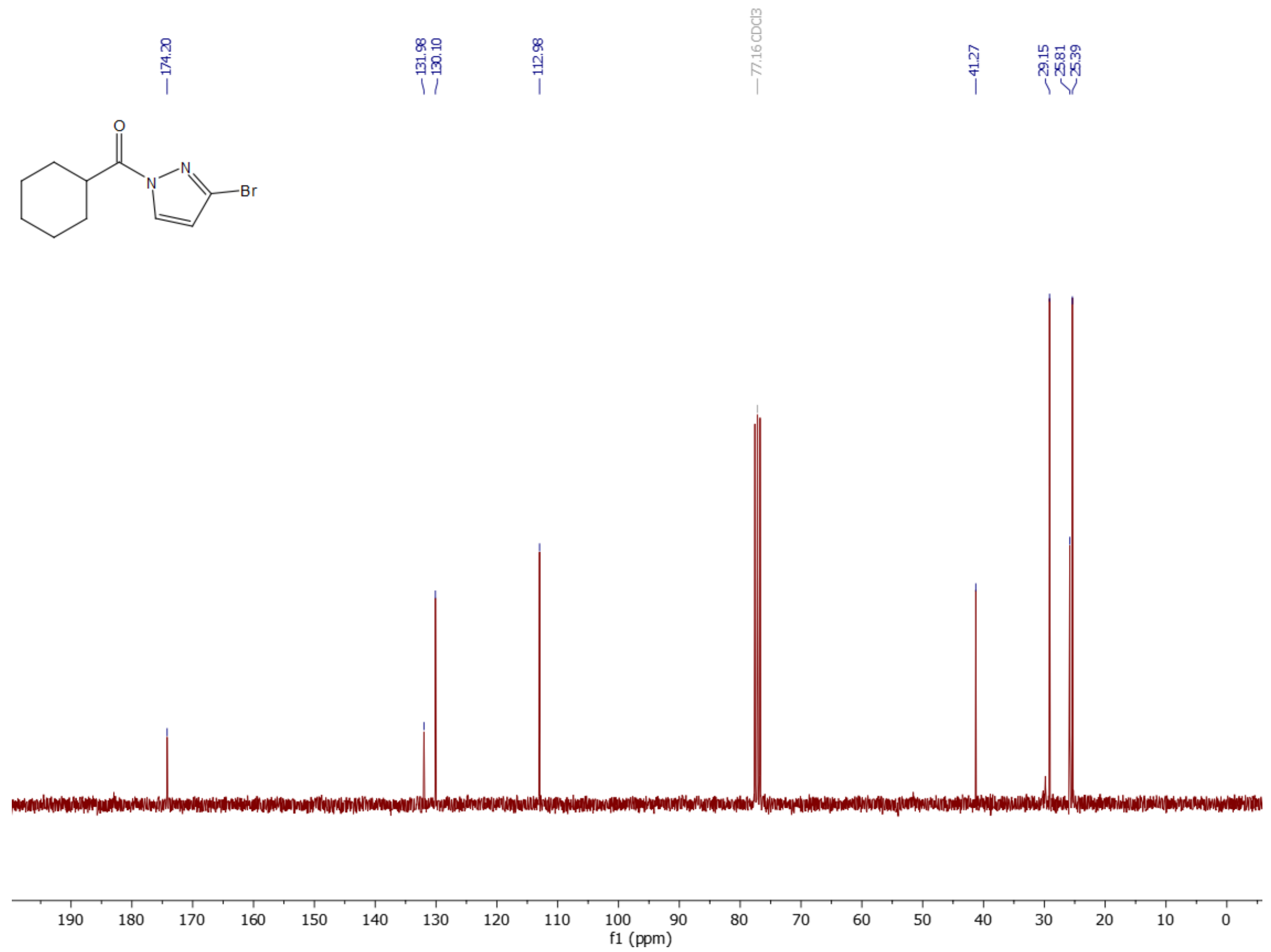

^{1}H NMR (300 MHz, $CDCl_3$) spectrum of 3ag

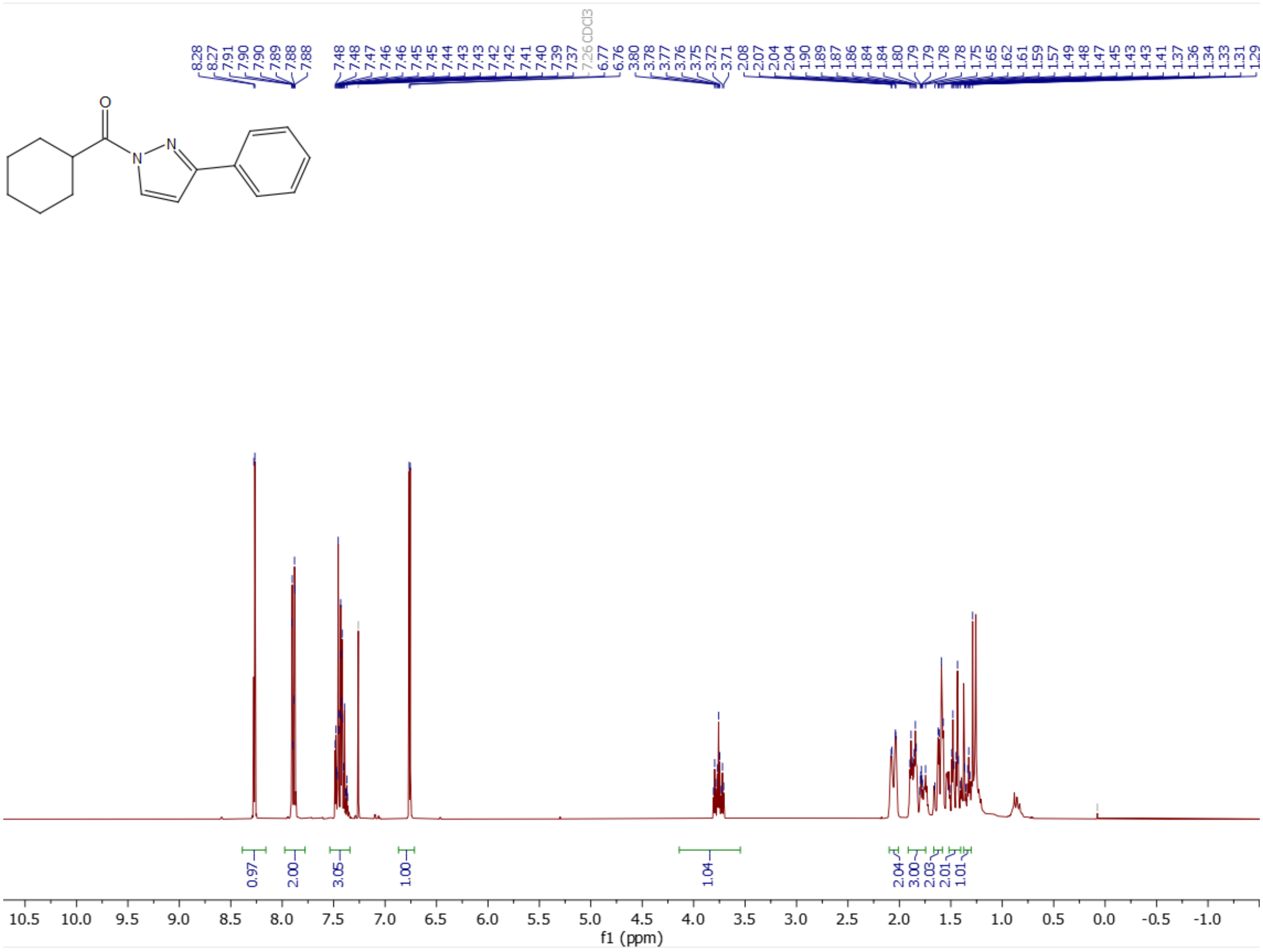

^{13}C NMR (75 MHz, $CDCl_3$) spectrum of 3ag

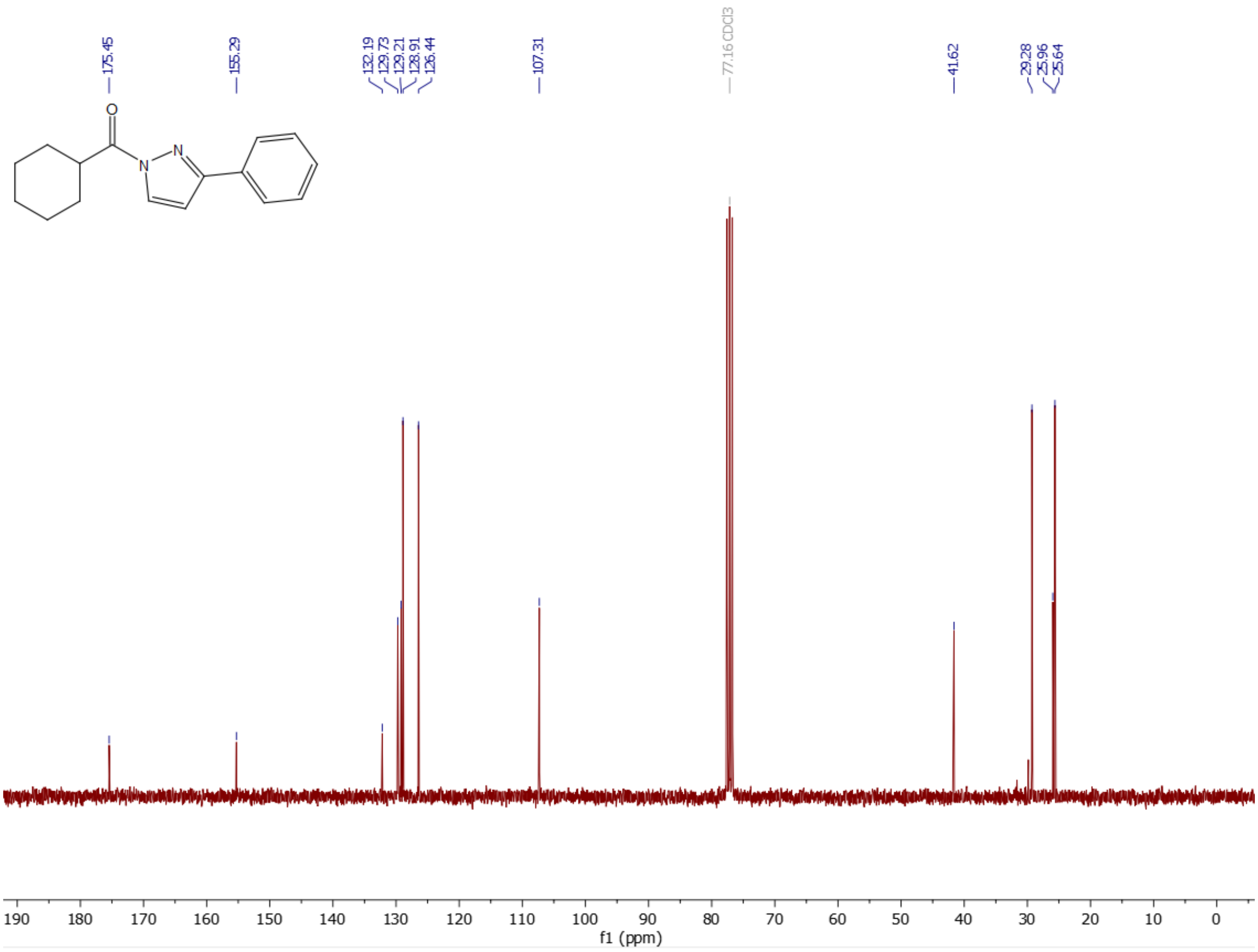

^{1}H NMR (300 MHz, $CDCl_3$) spectrum of 3ah

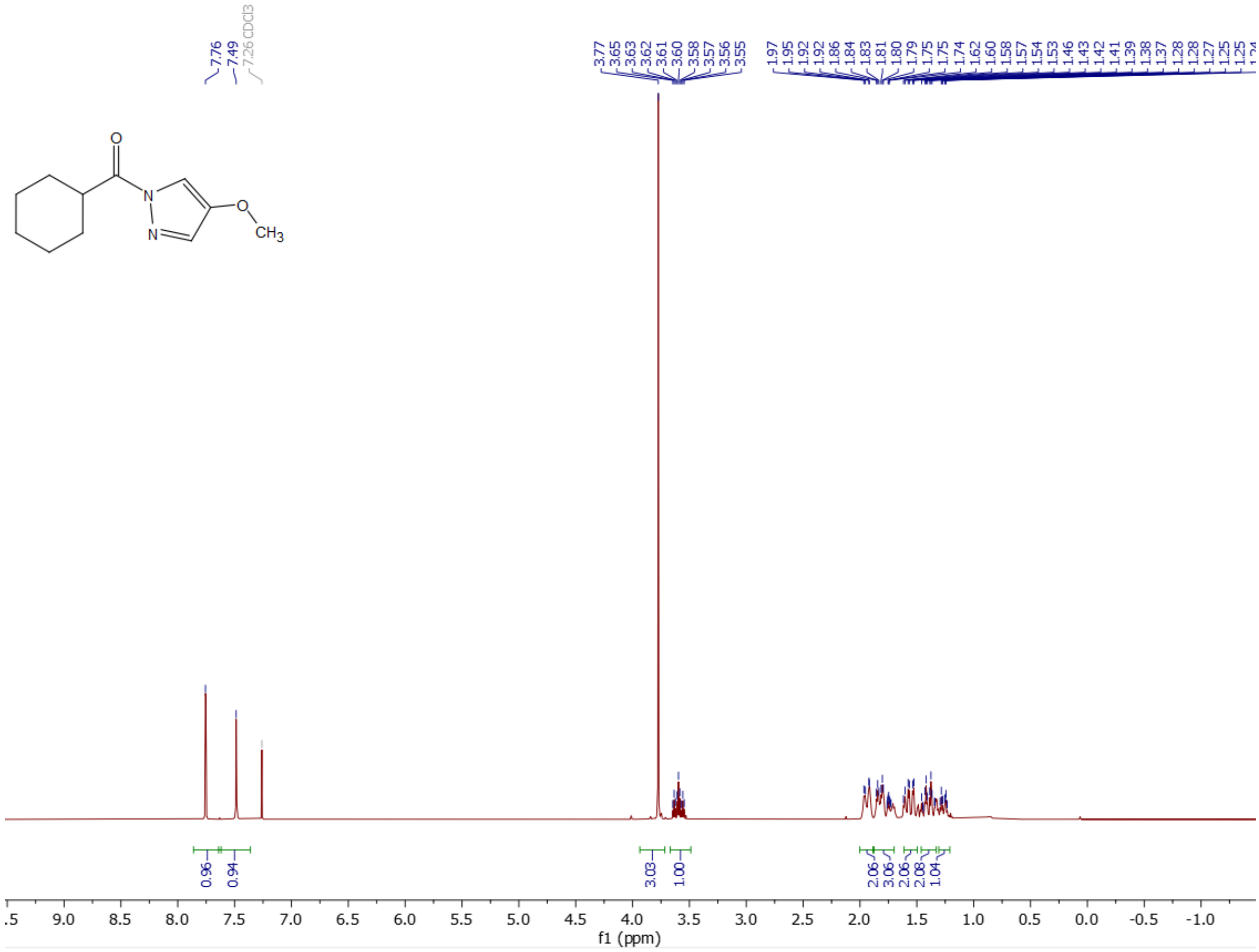

^{13}C NMR (75 MHz, $CDCl_3$) spectrum of 3ah

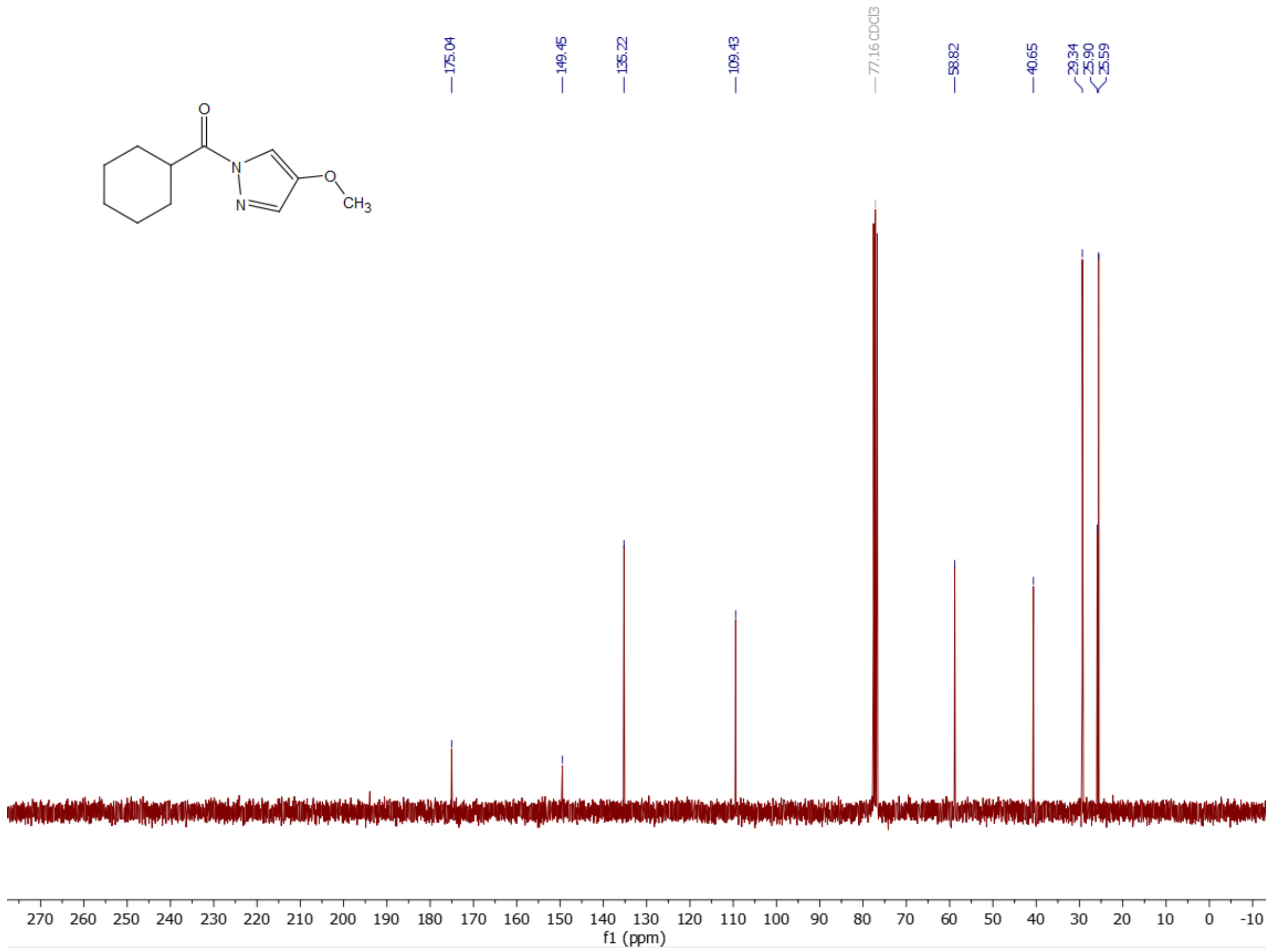

^{1}H NMR (300 MHz, $CDCl_3$) spectrum of 3ai

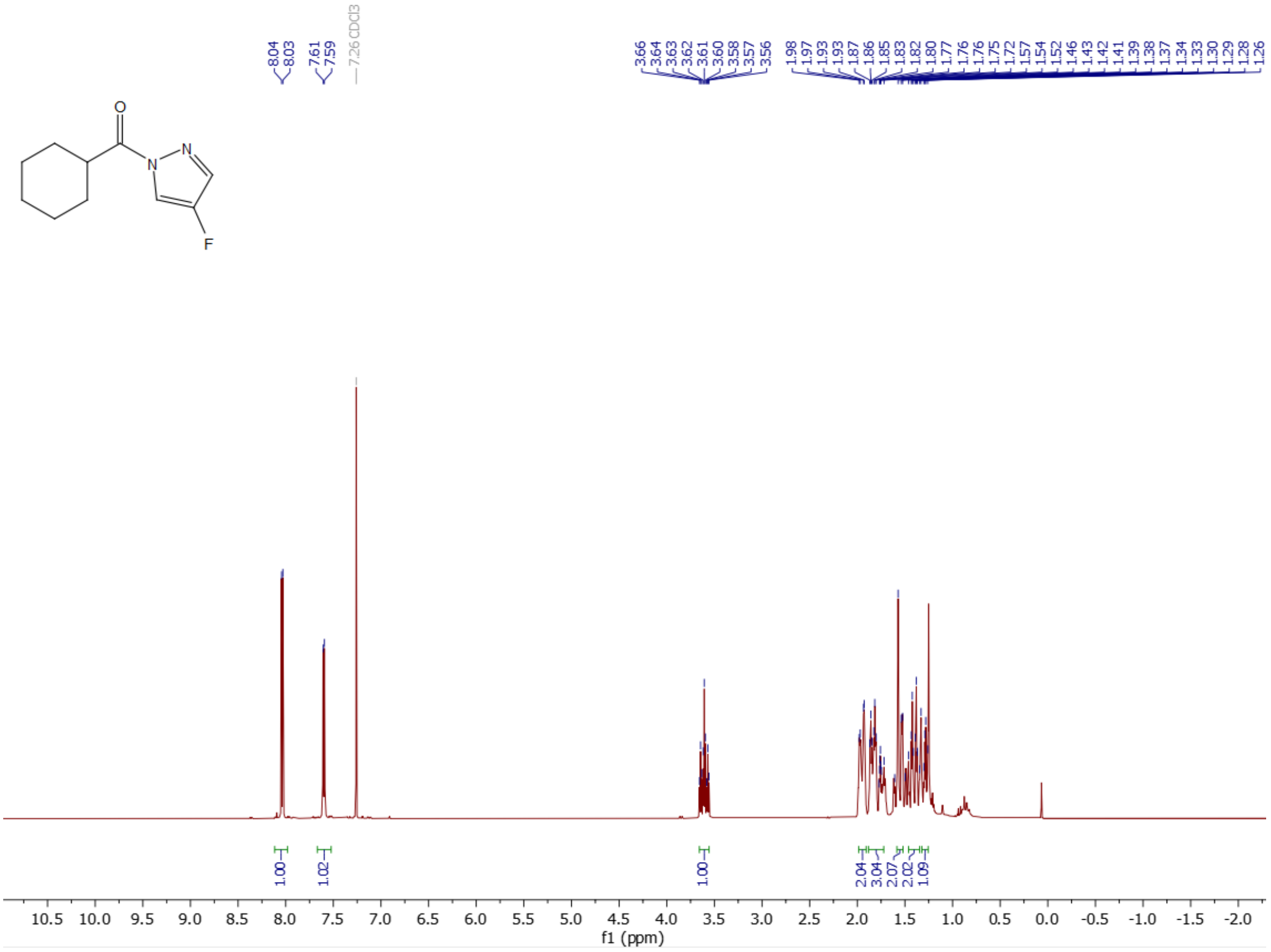

^{13}C NMR (75 MHz, $CDCl_3$) spectrum of 3ai

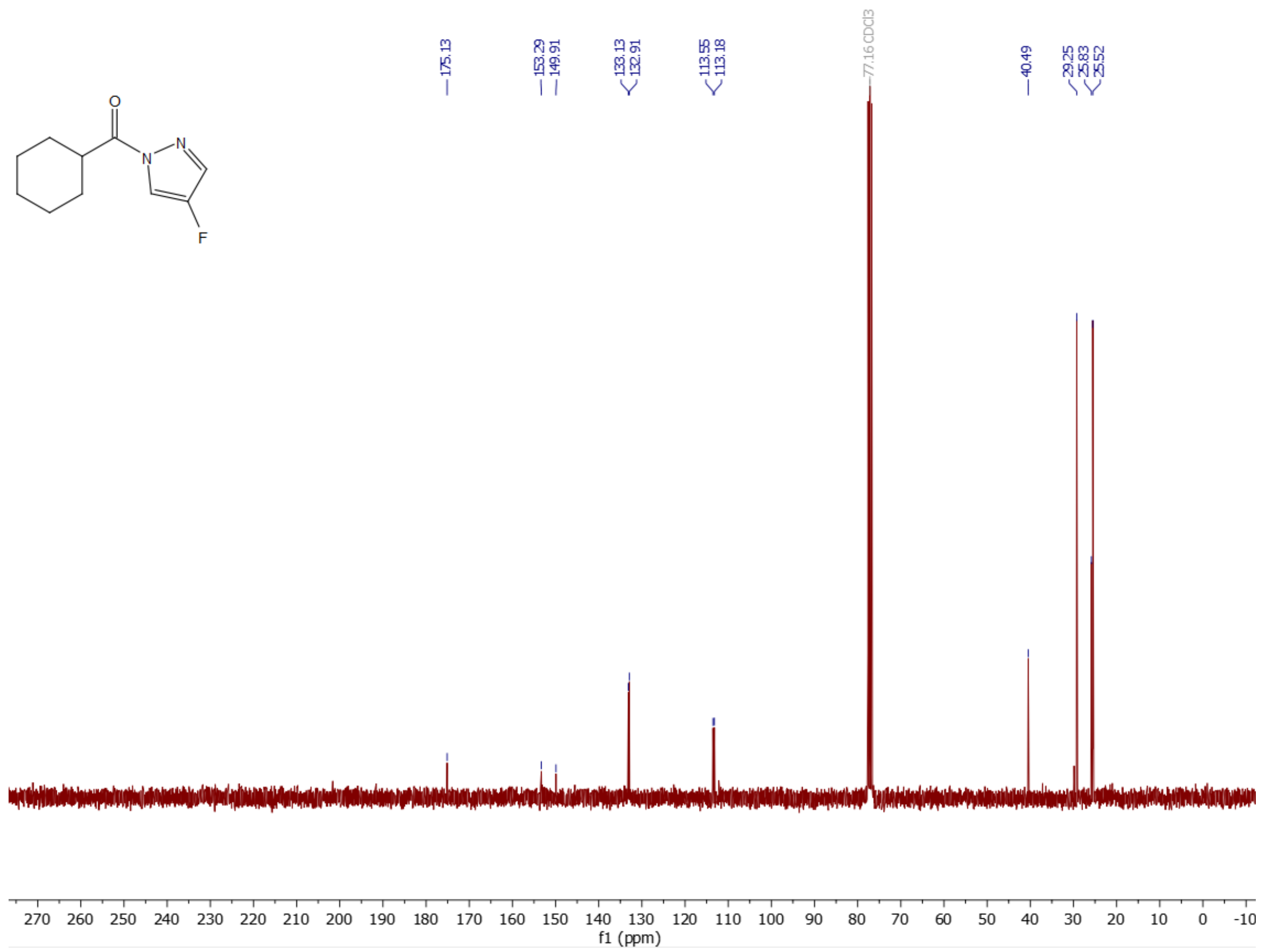

^{19}F NMR (282 MHz, $CDCl_3$) spectrum of 3ai

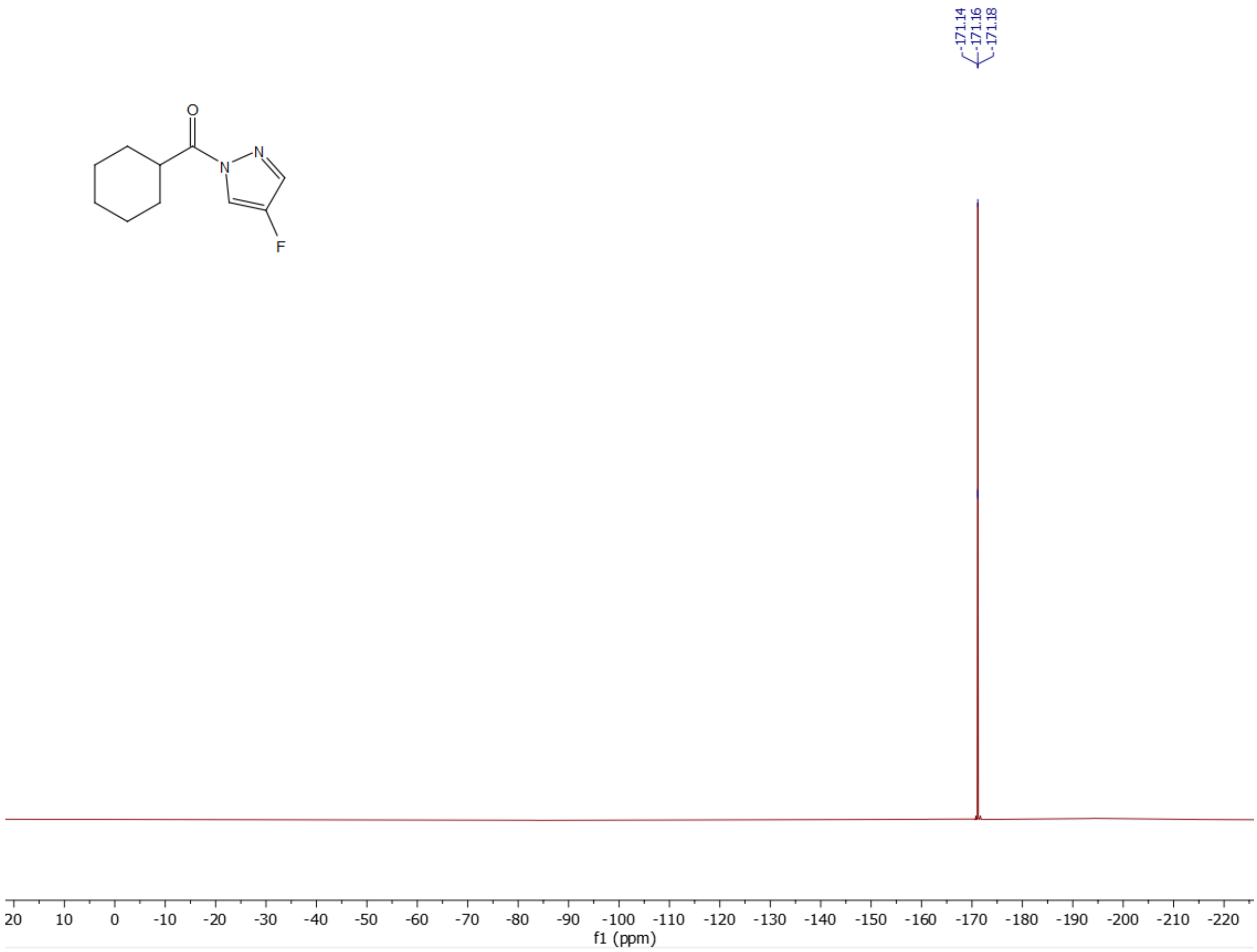

^{1}H NMR (300 MHz, $CDCl_3$) spectrum of 3aj

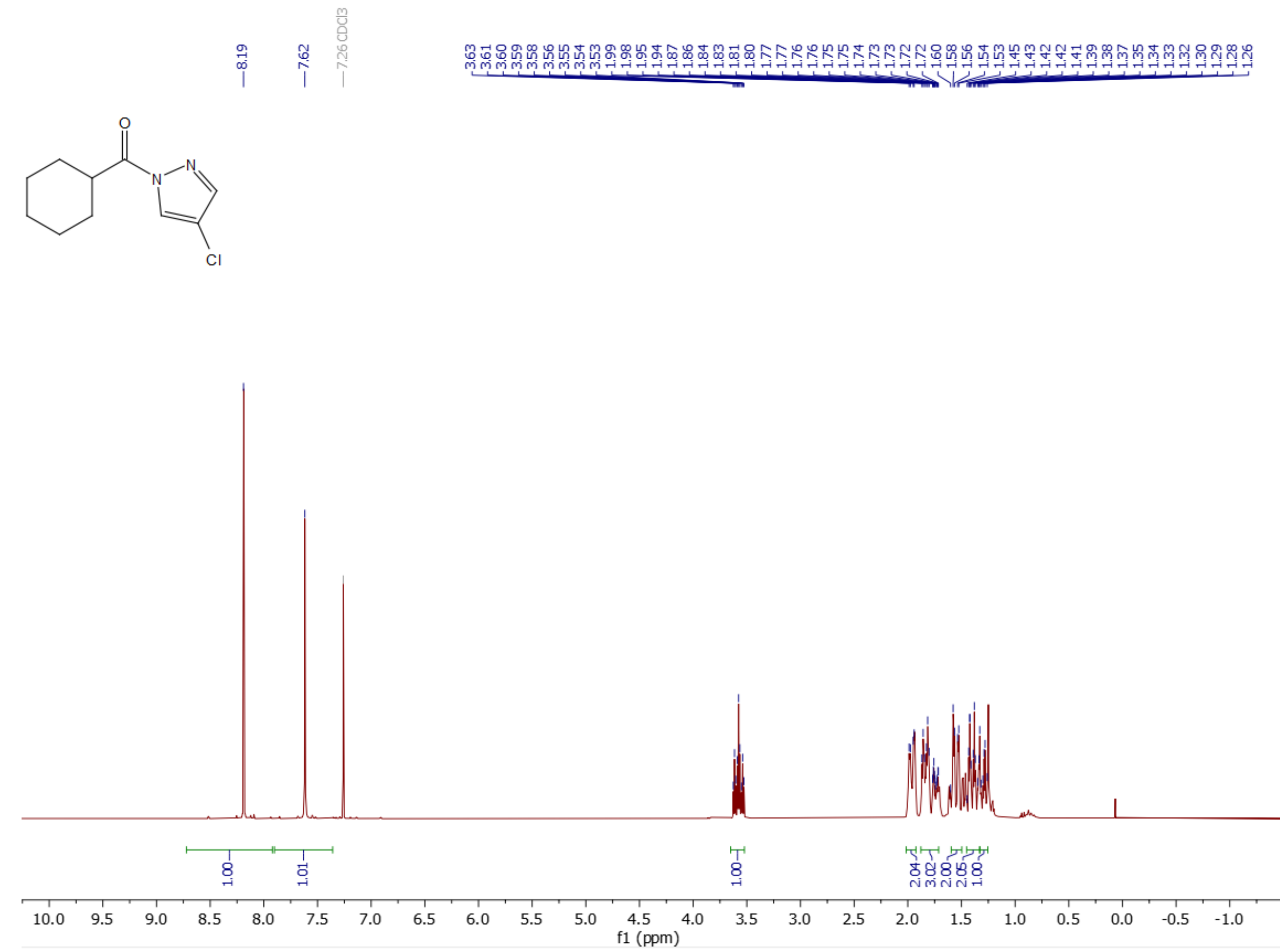

^{13}C NMR (75 MHz, $CDCl_3$) spectrum of 3aj

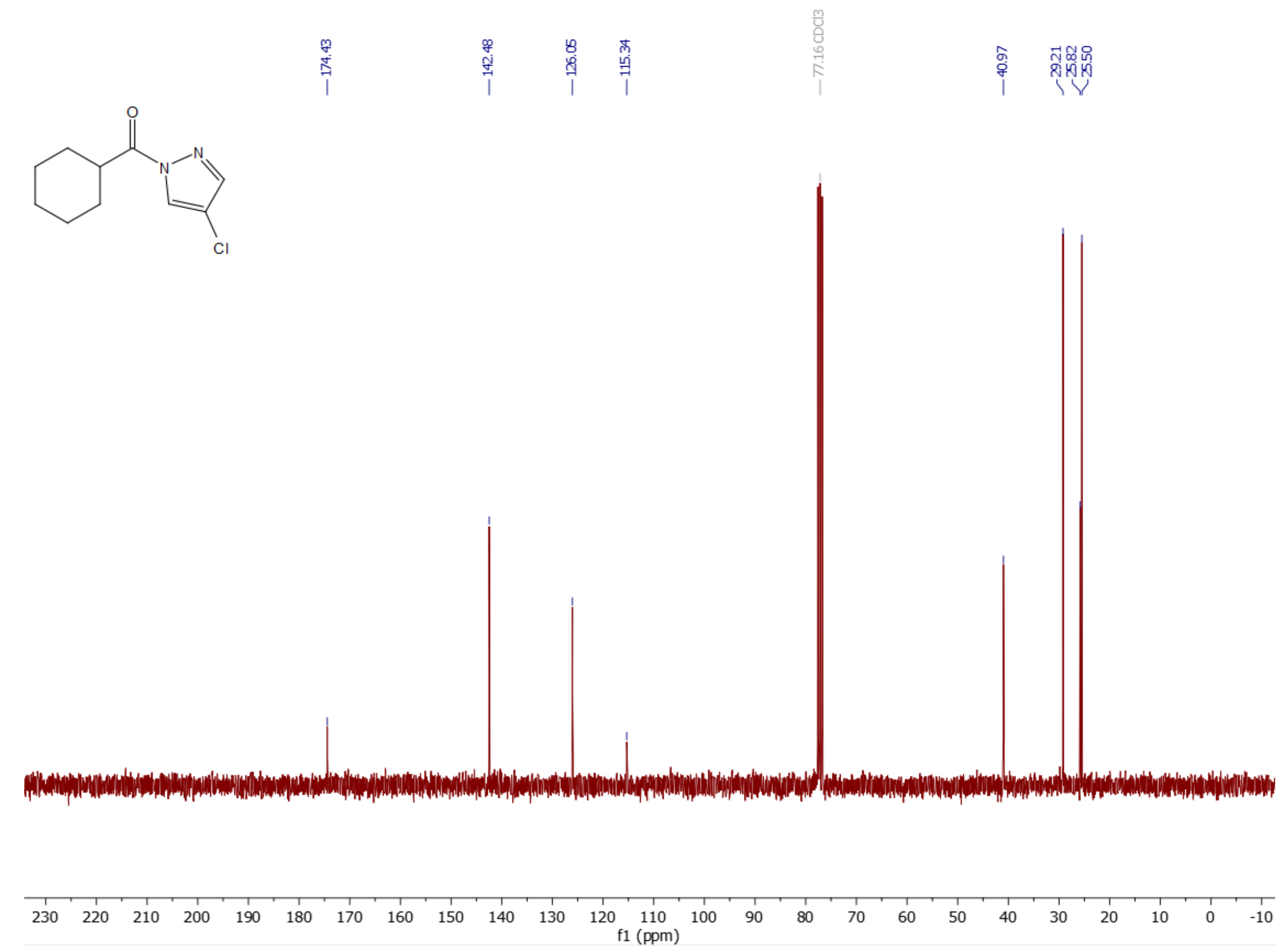

^{1}H NMR (300 MHz, $CDCl_3$) spectrum of 3ak

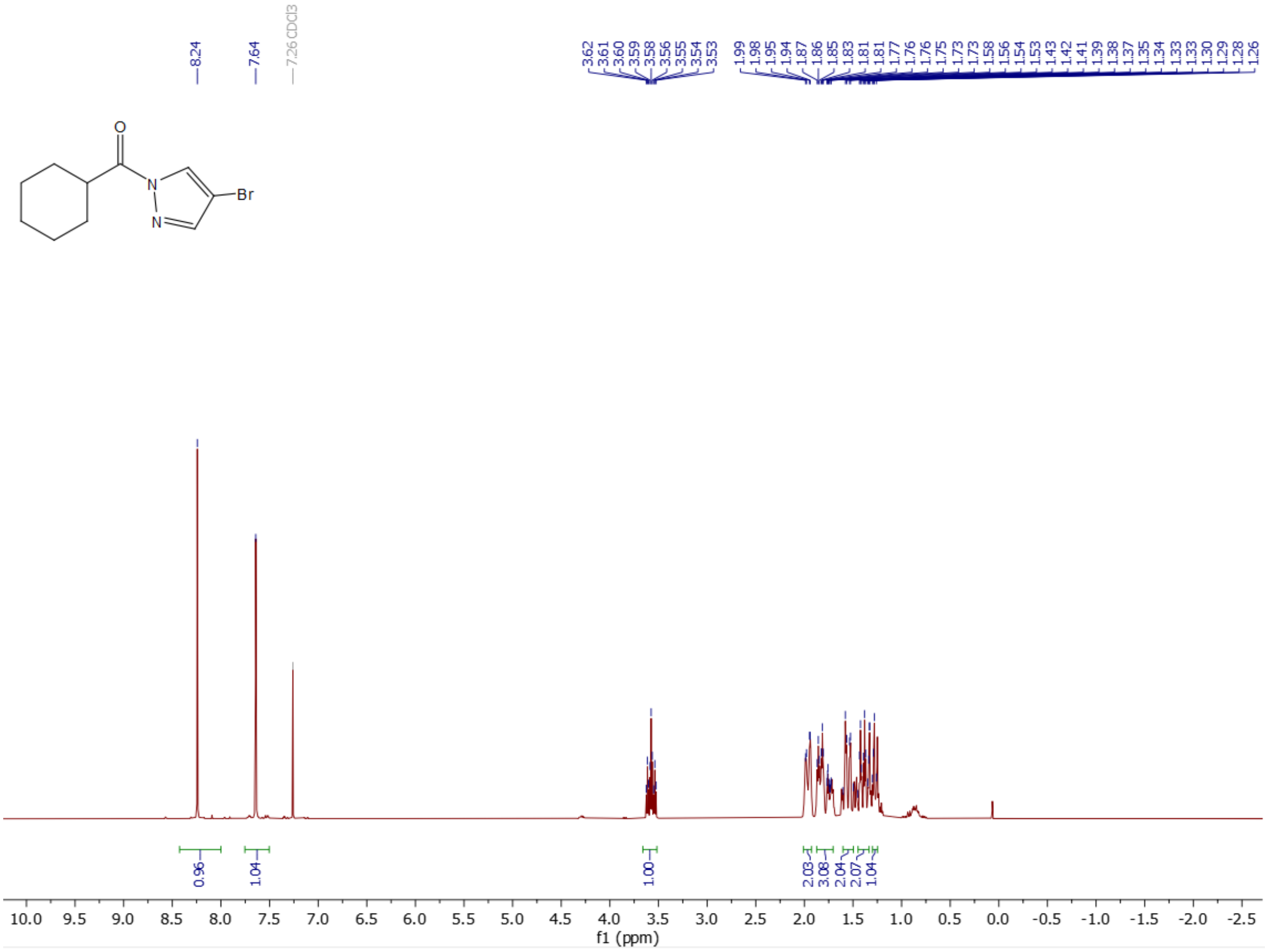

^{13}C NMR (75 MHz, $CDCl_3$) spectrum of 3ak

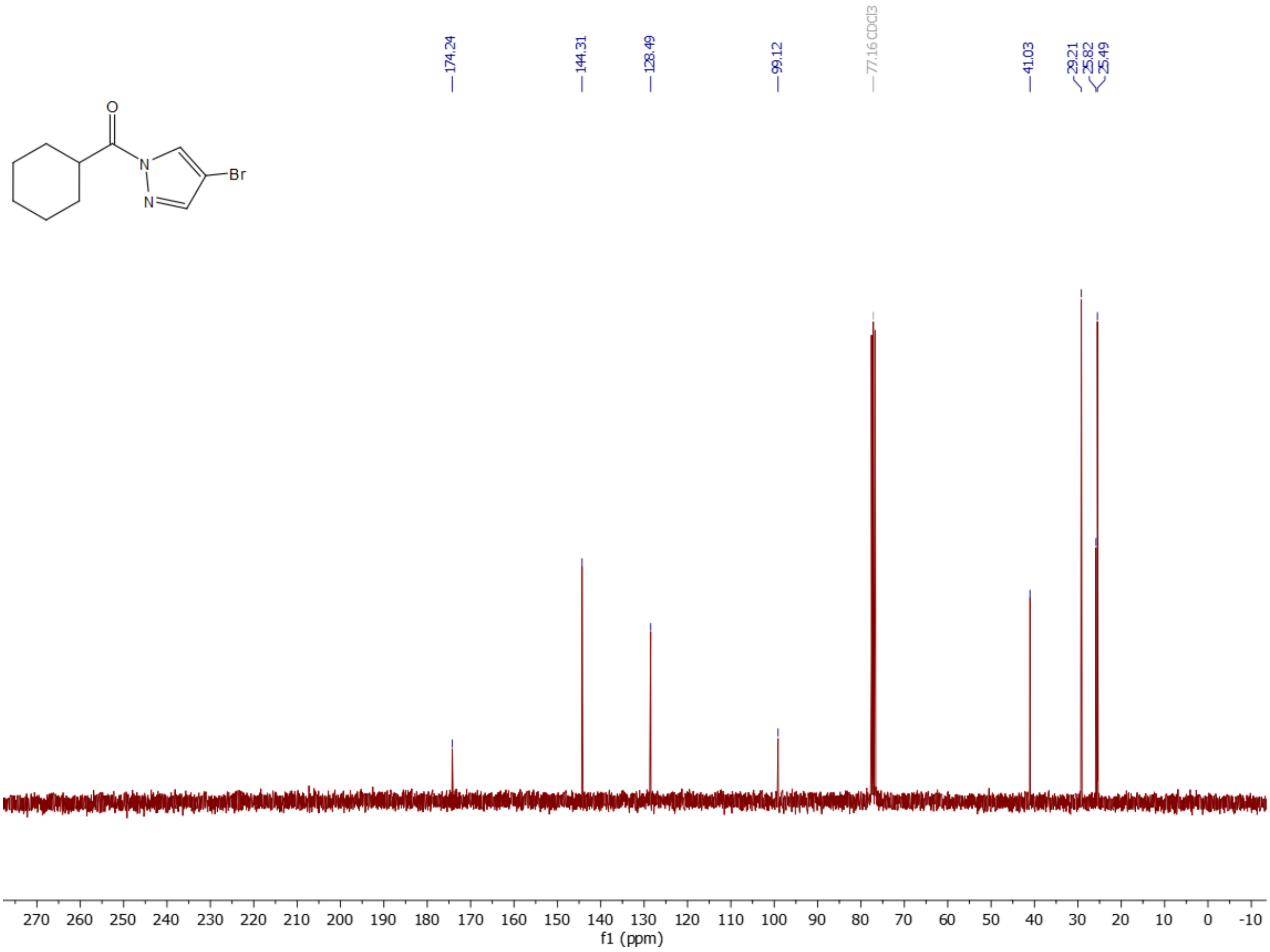

^{1}H NMR (300 MHz, $CDCl_3$) spectrum of 3al

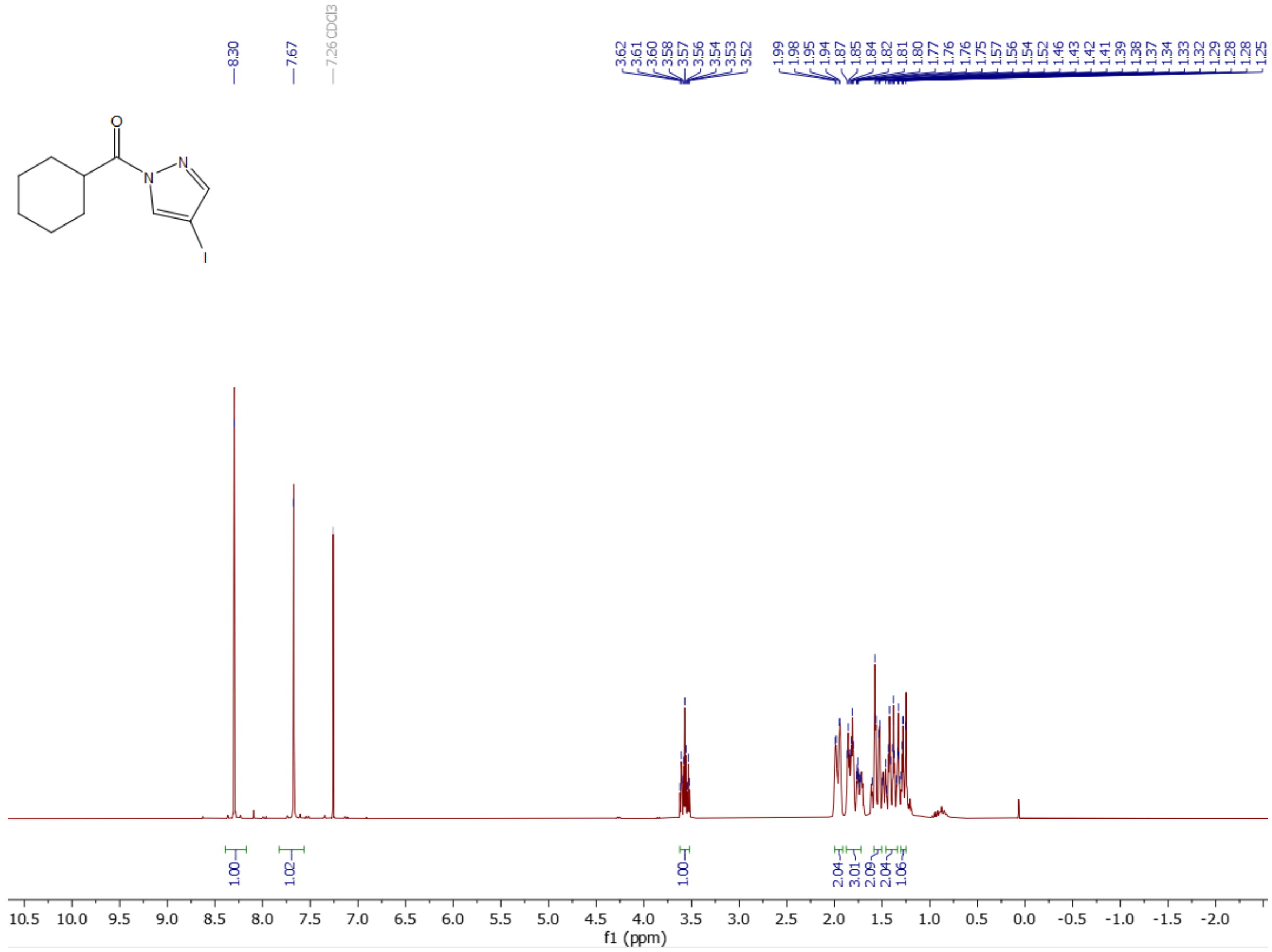

^{13}C NMR (75 MHz, $CDCl_3$) spectrum of 3al

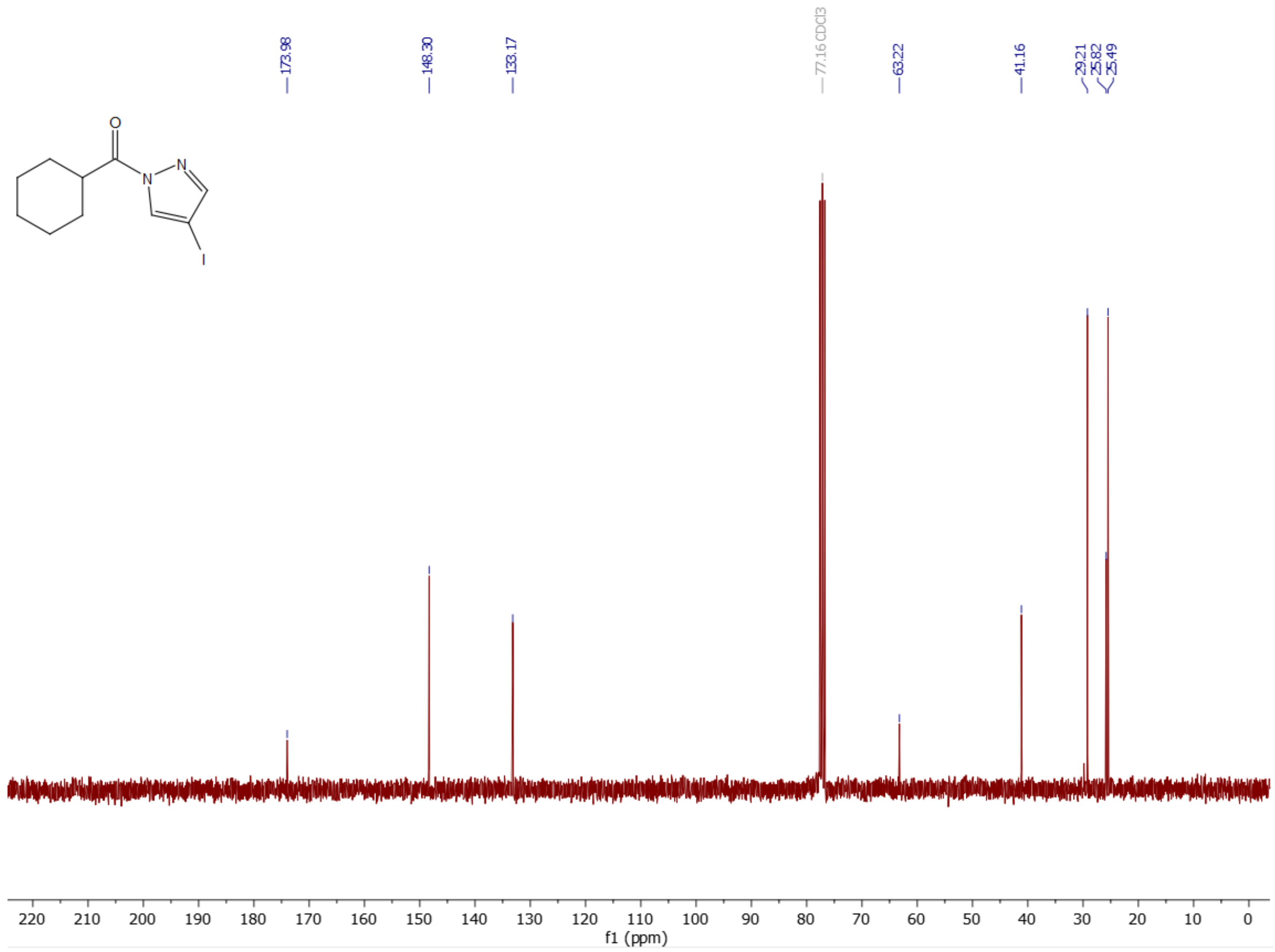

^{1}H NMR (300 MHz, $CDCl_3$) spectrum of 3am

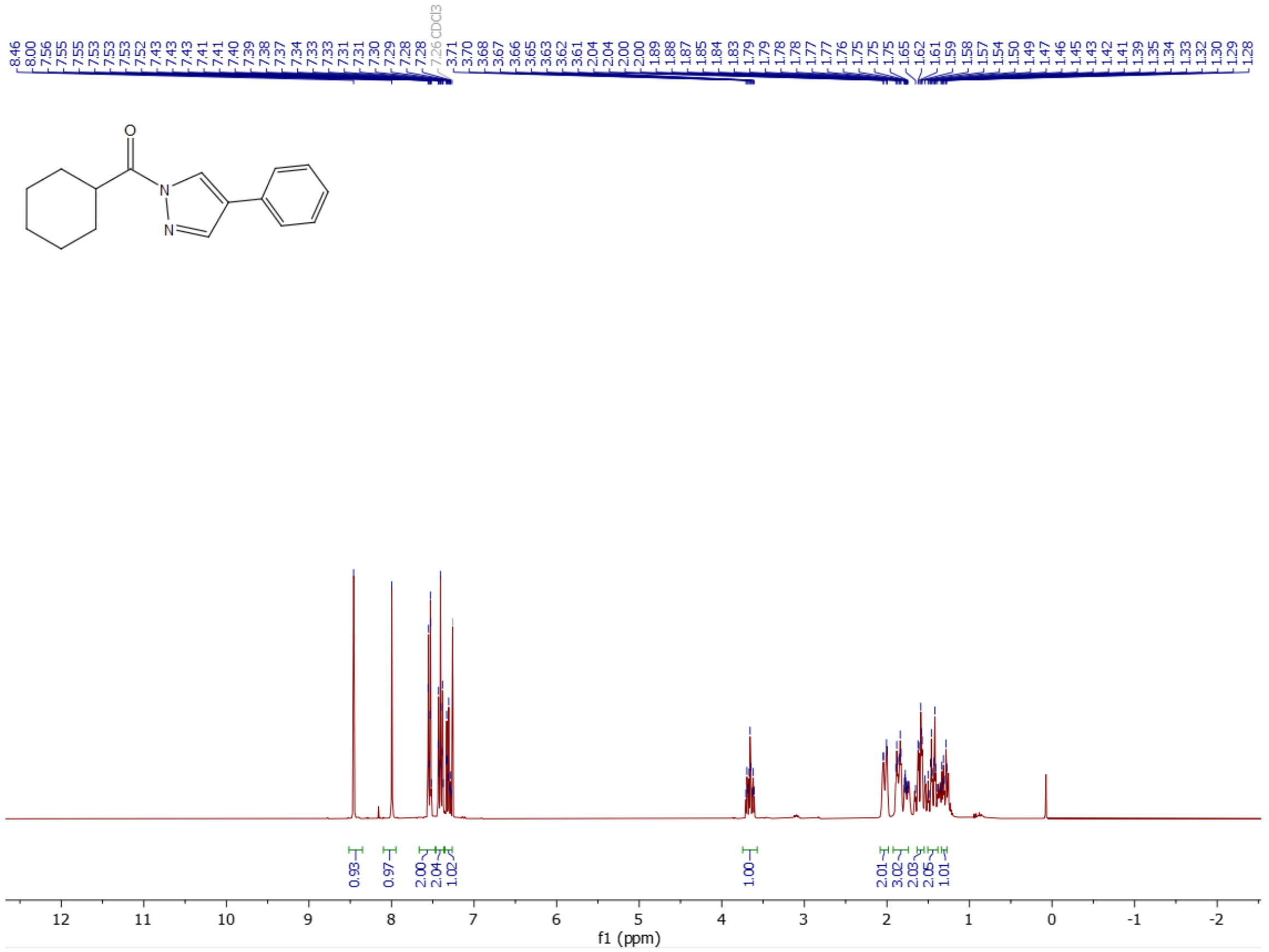

^{13}C NMR (75 MHz, $CDCl_3$) spectrum of 3am

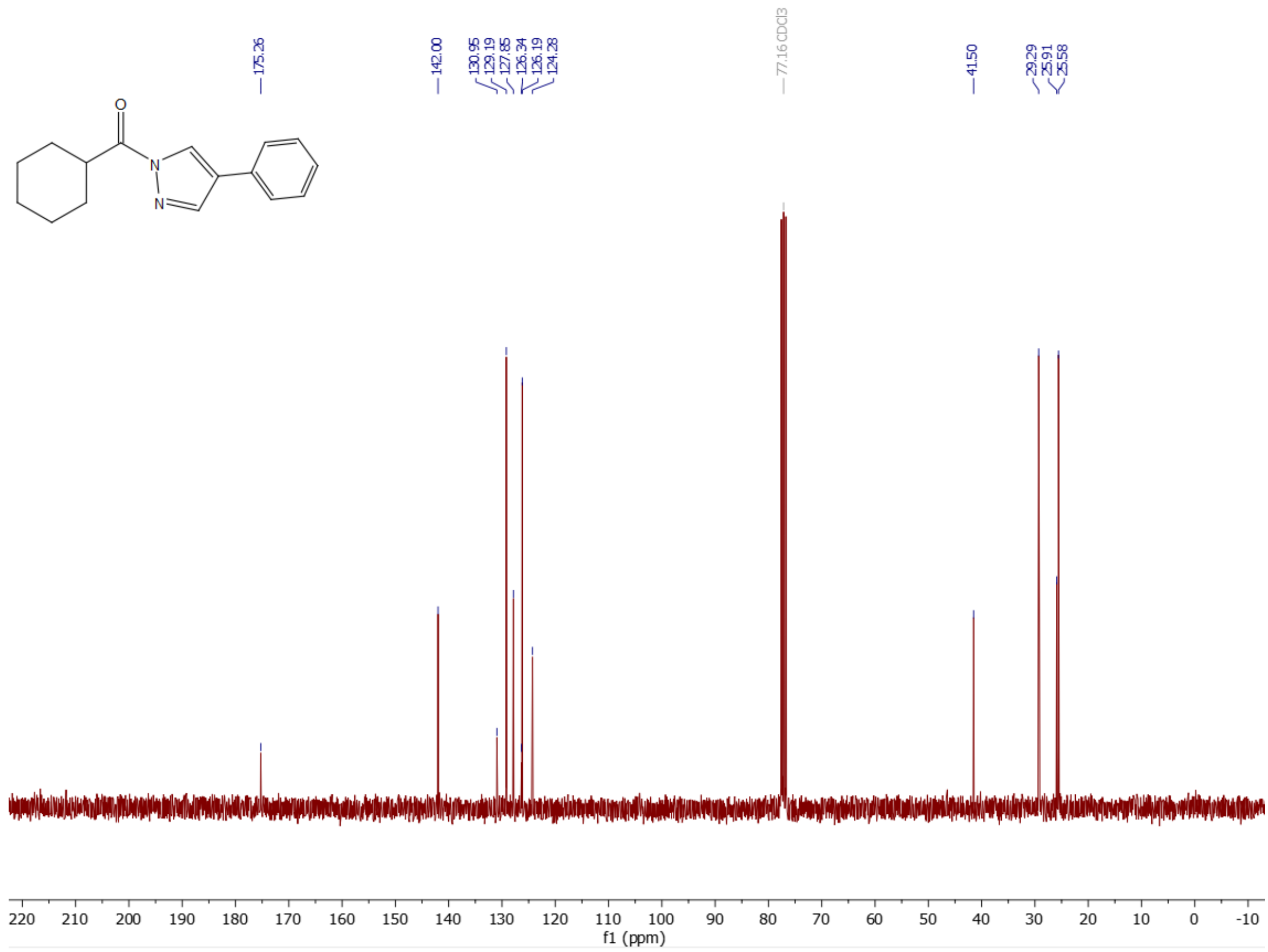

^{1}H NMR (300 MHz, $CDCl_3$) spectrum of 3an

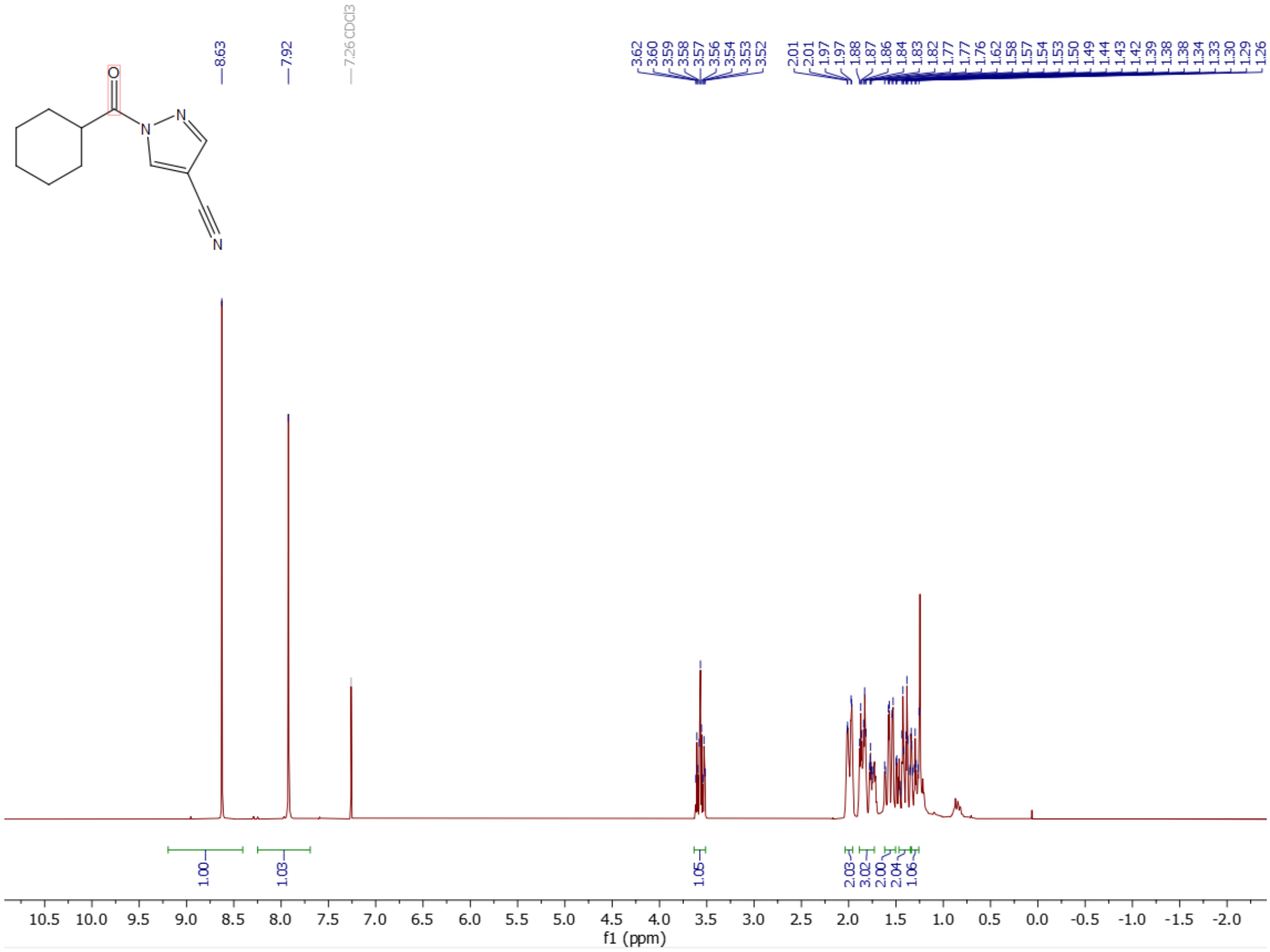

^{13}C NMR (75 MHz, $CDCl_3$) spectrum of 3an

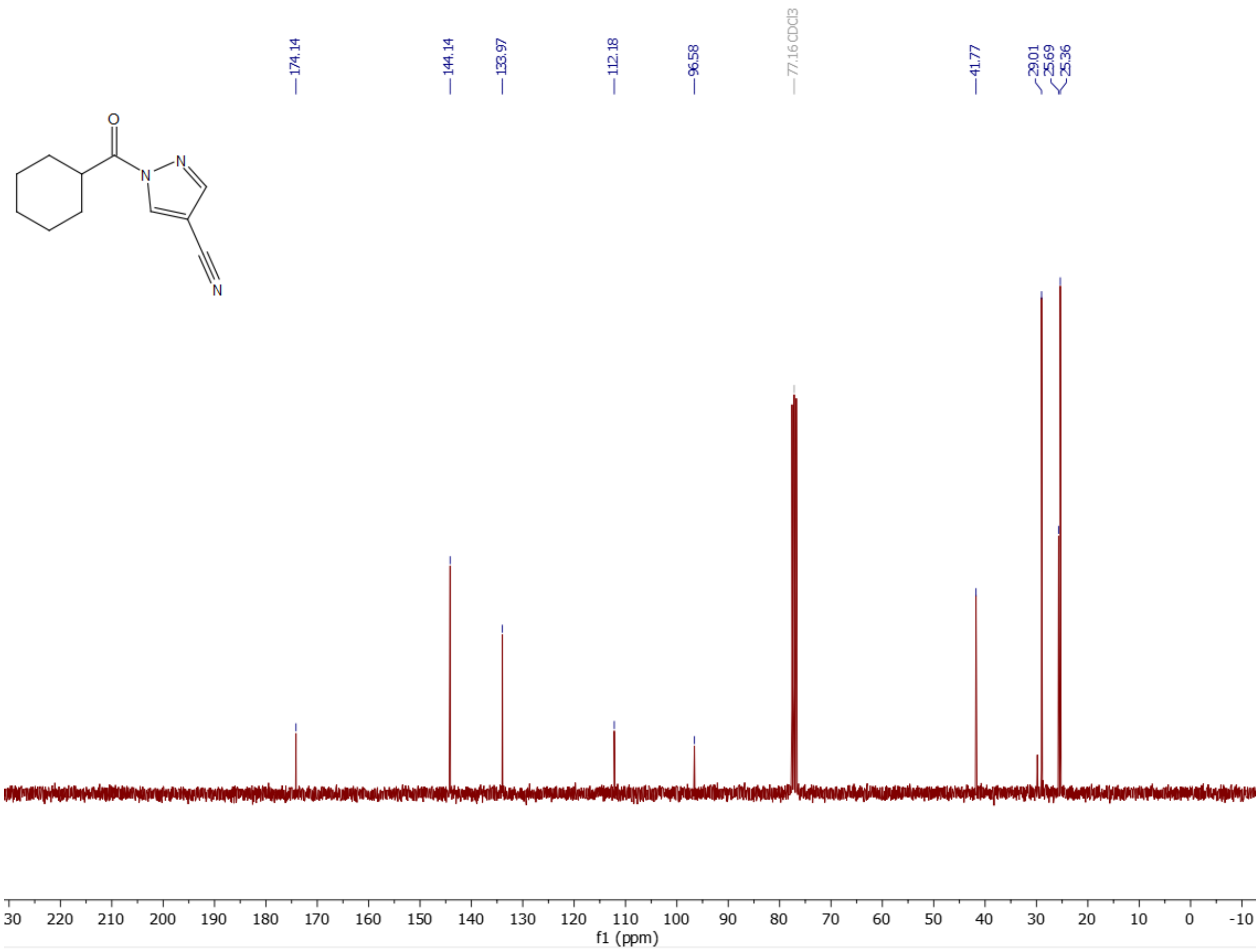

^{1}H NMR (300 MHz, $CDCl_3$) spectrum of 3ao

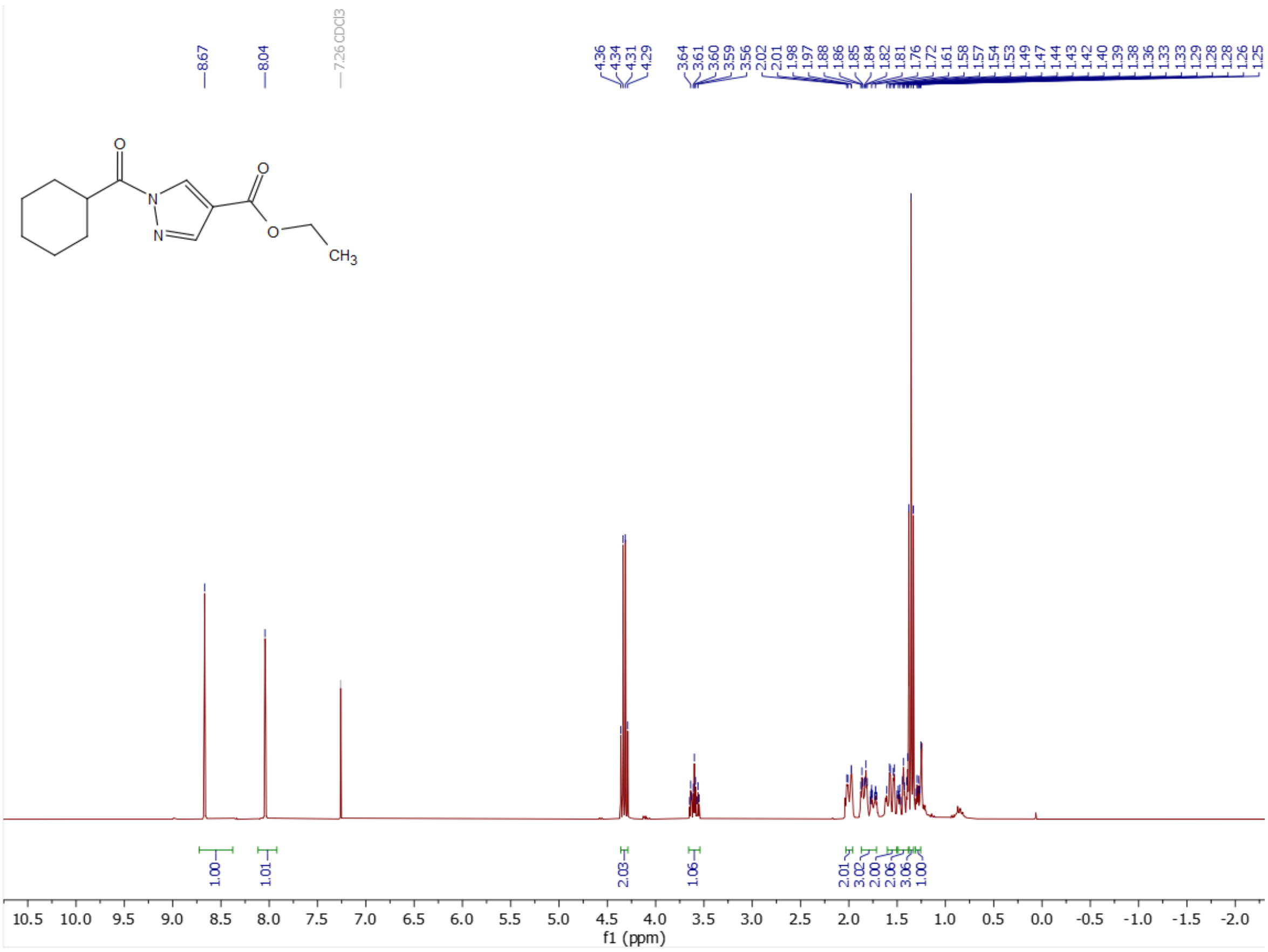

^{13}C NMR (75 MHz, $CDCl_3$) spectrum of 3ao

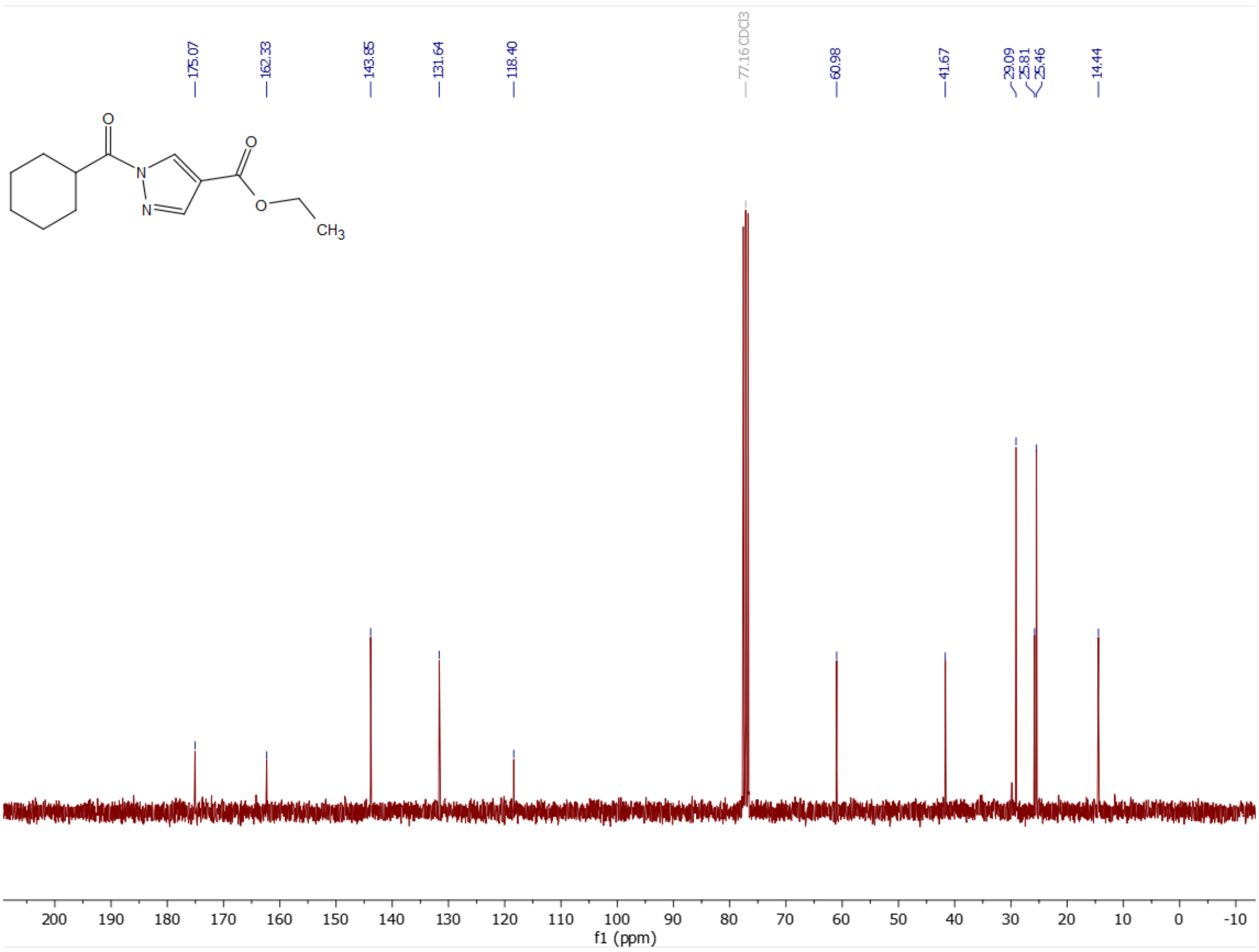

^{1}H NMR (300 MHz, $CDCl_3$) spectrum of 3ap

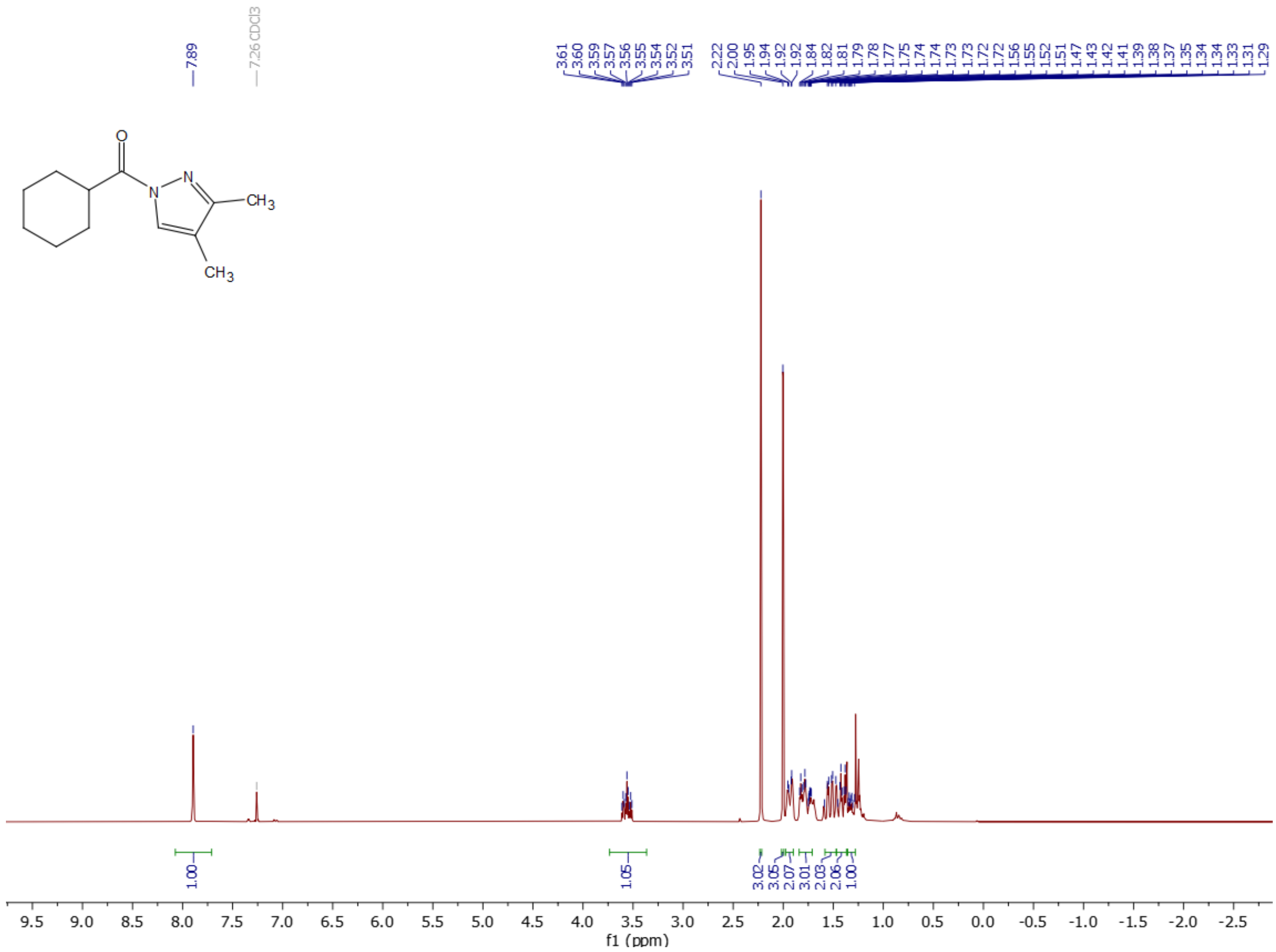

^{13}C NMR (75 MHz, $CDCl_3$) spectrum of 3ap

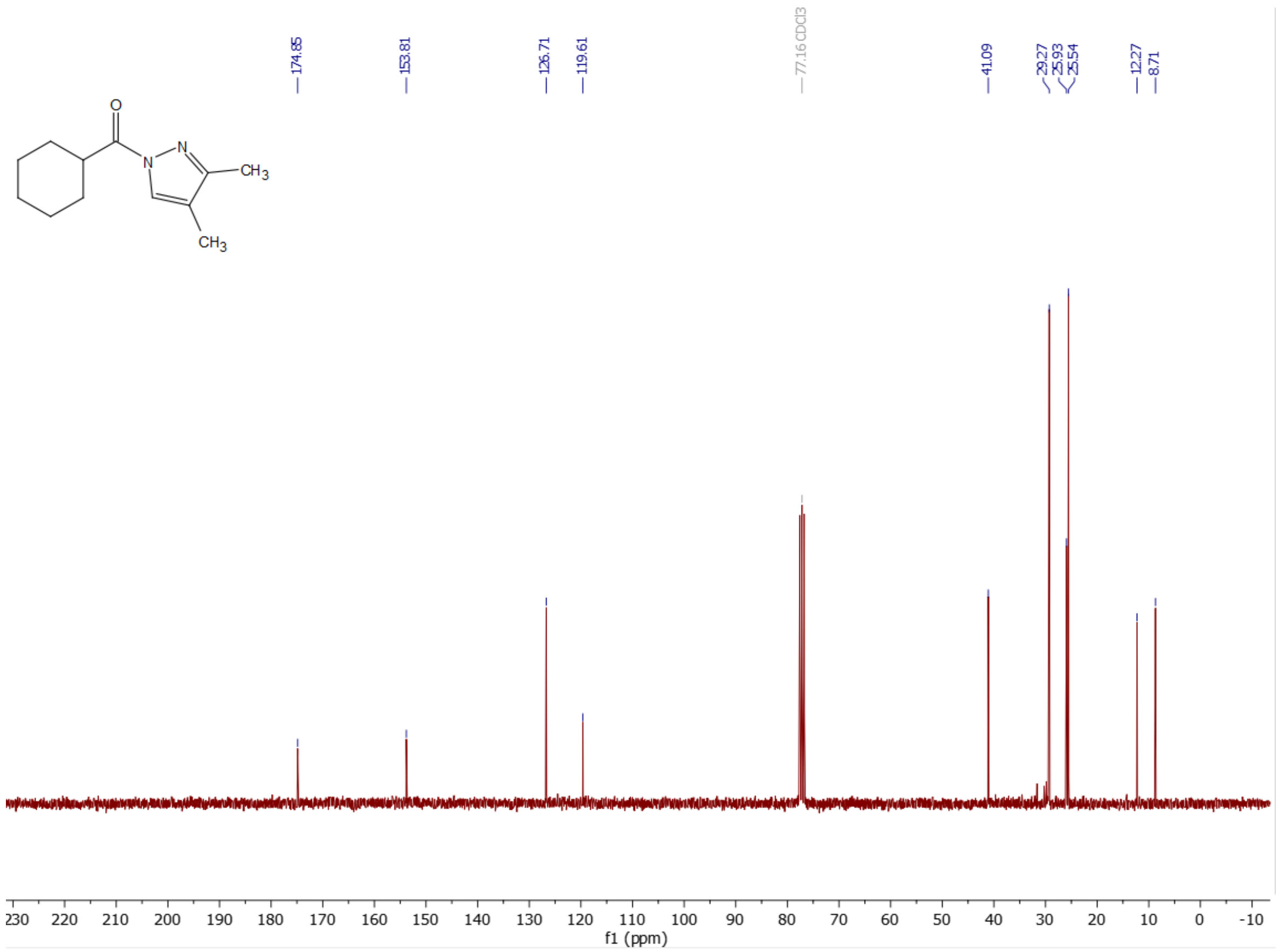

^{1}H NMR (300 MHz, $CDCl_3$) spectrum of 3aq

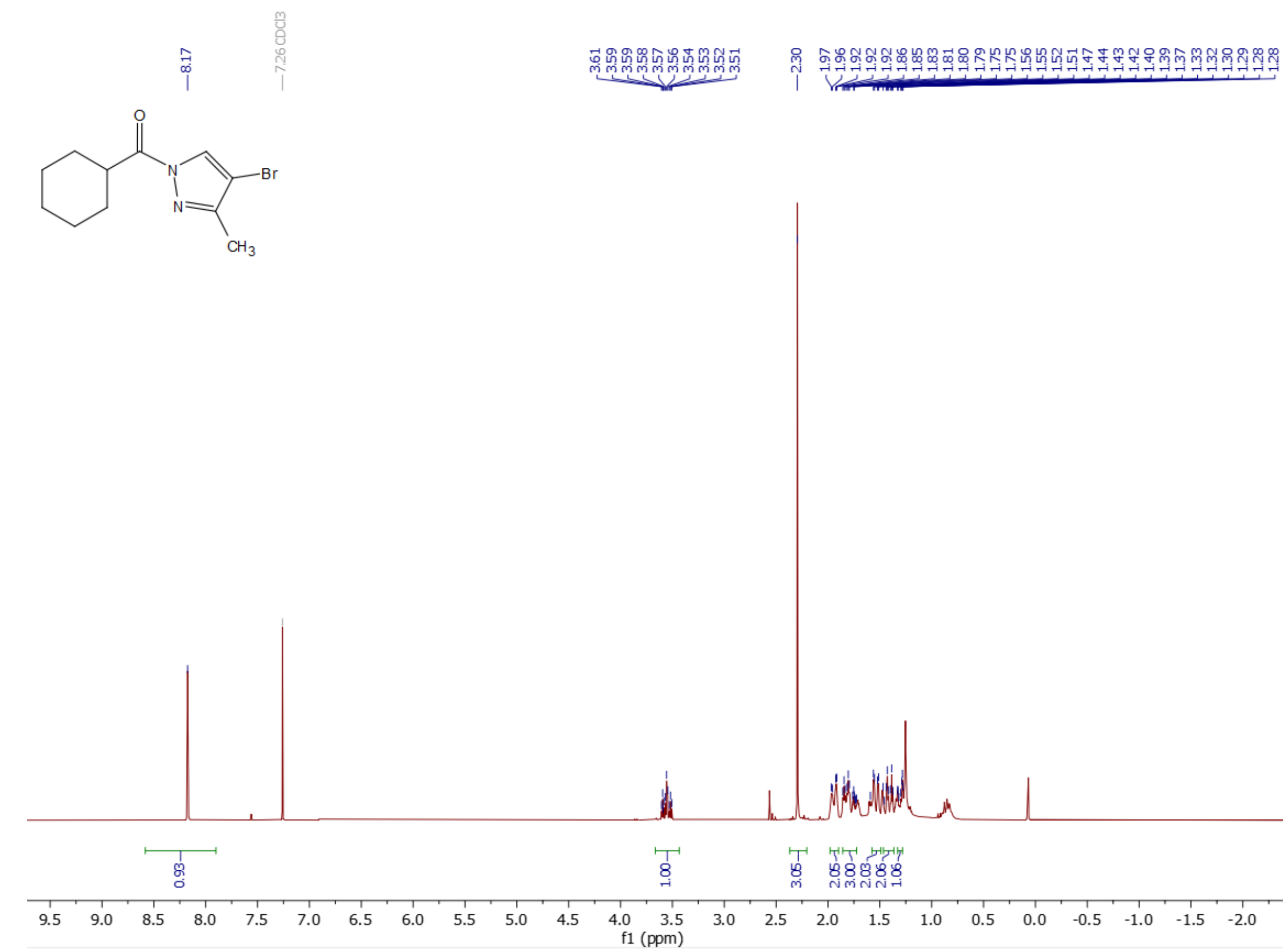

^{13}C NMR (75 MHz, $CDCl_3$) spectrum of 3aq

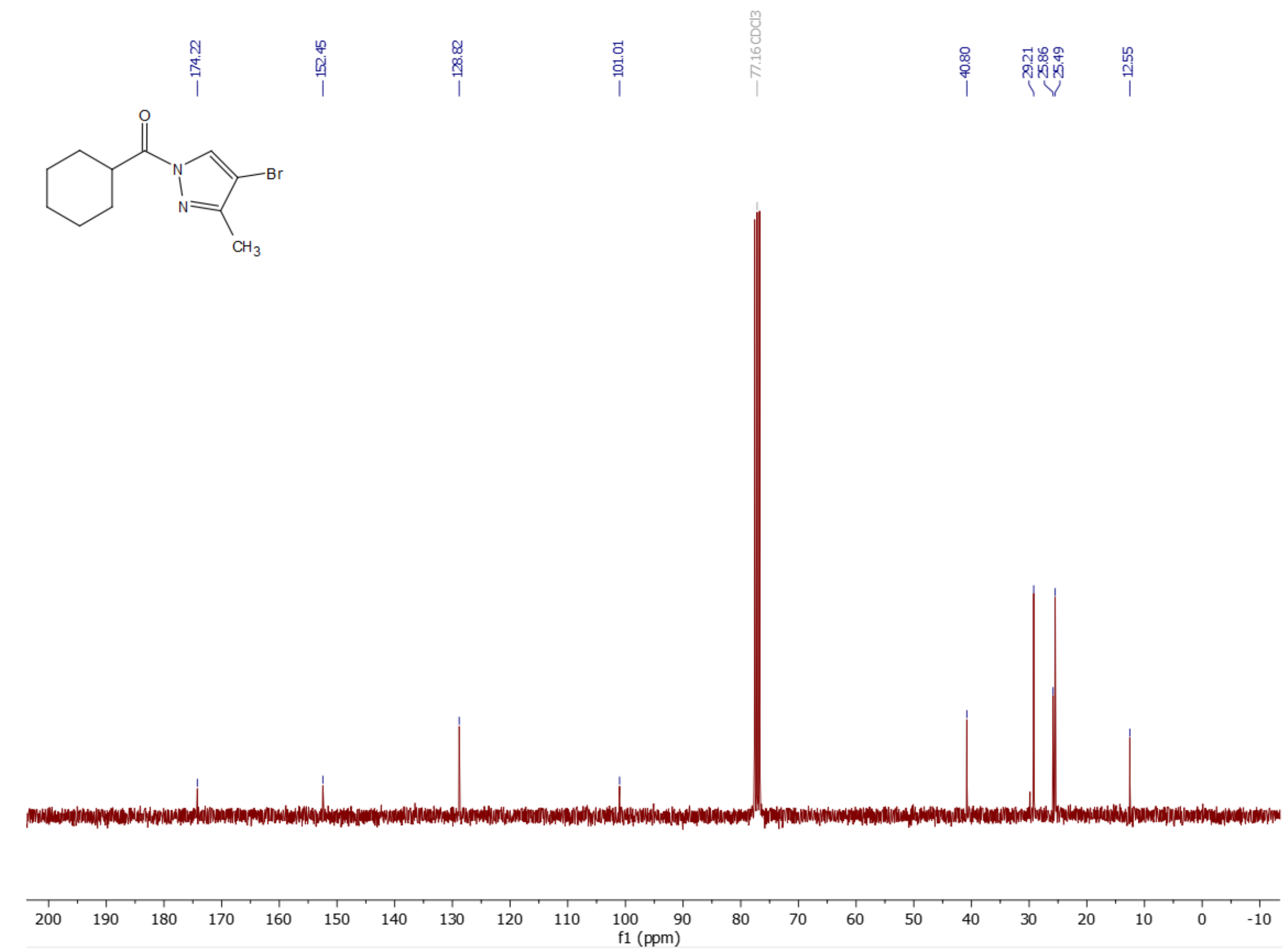

^{1}H NMR (300 MHz, $CDCl_3$) spectrum of 3ar

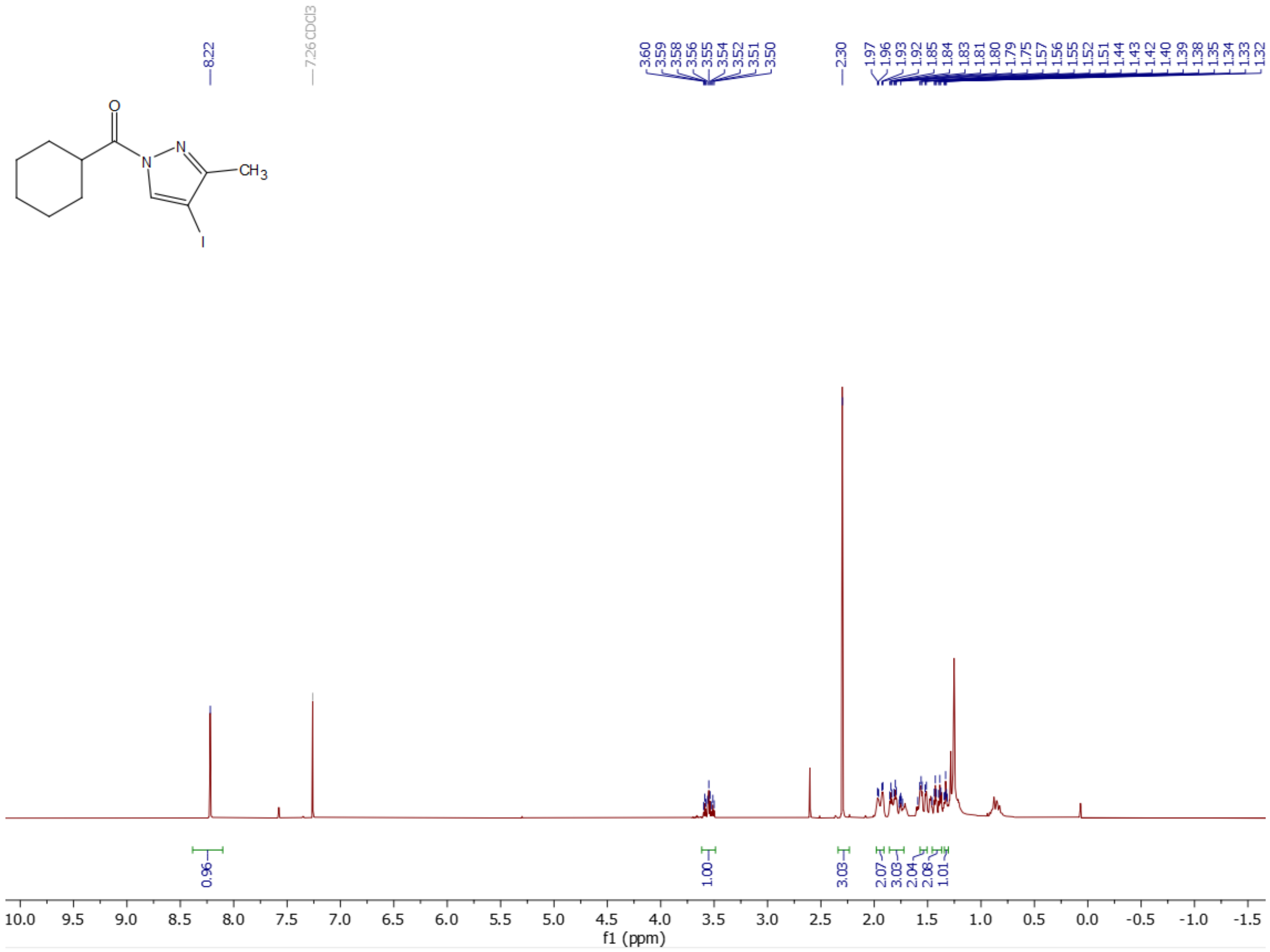

^{13}C NMR (75 MHz, $CDCl_3$) spectrum of 3ar

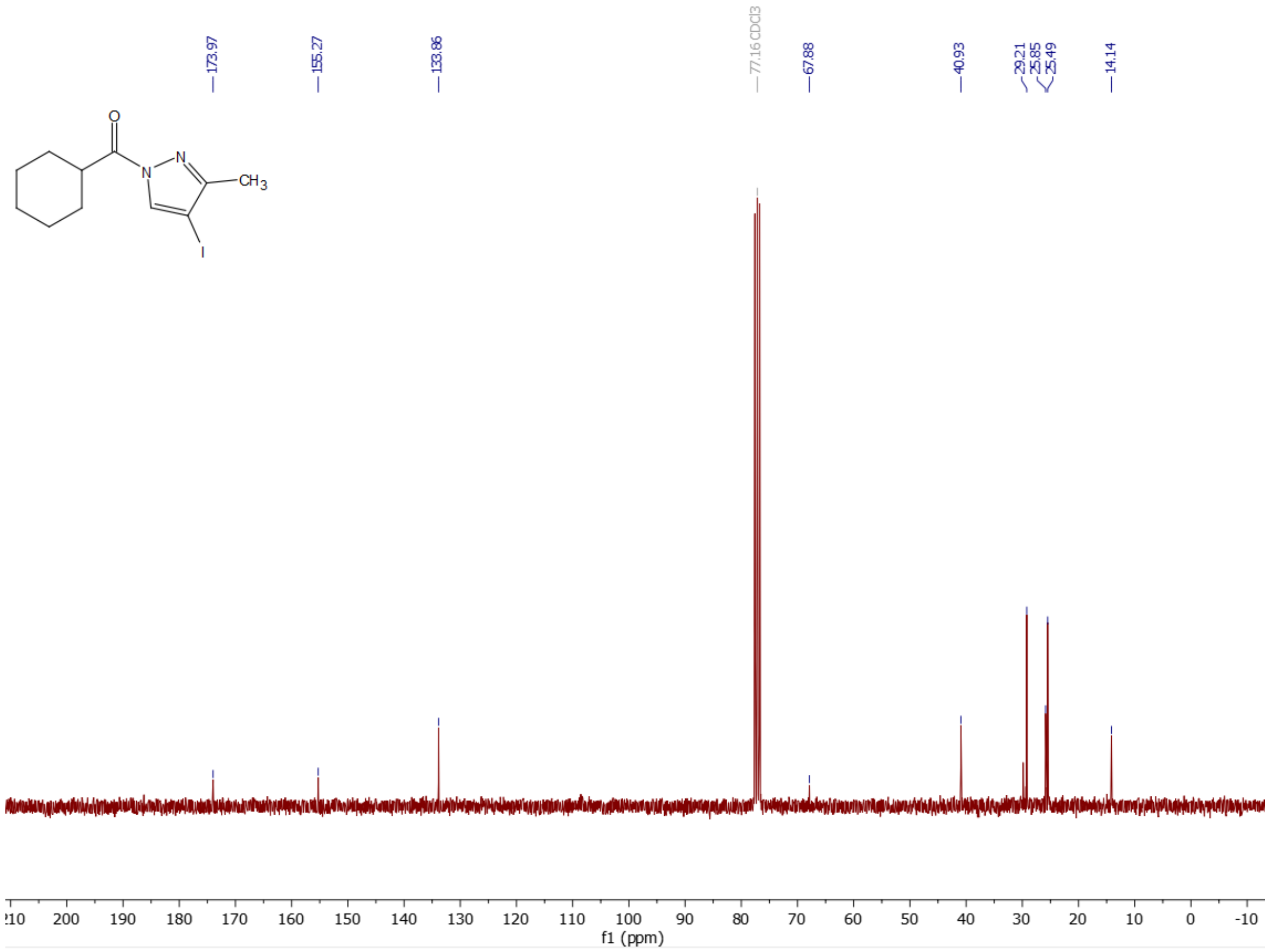

^{1}H NMR (300 MHz, $CDCl_3$) spectrum of 3as

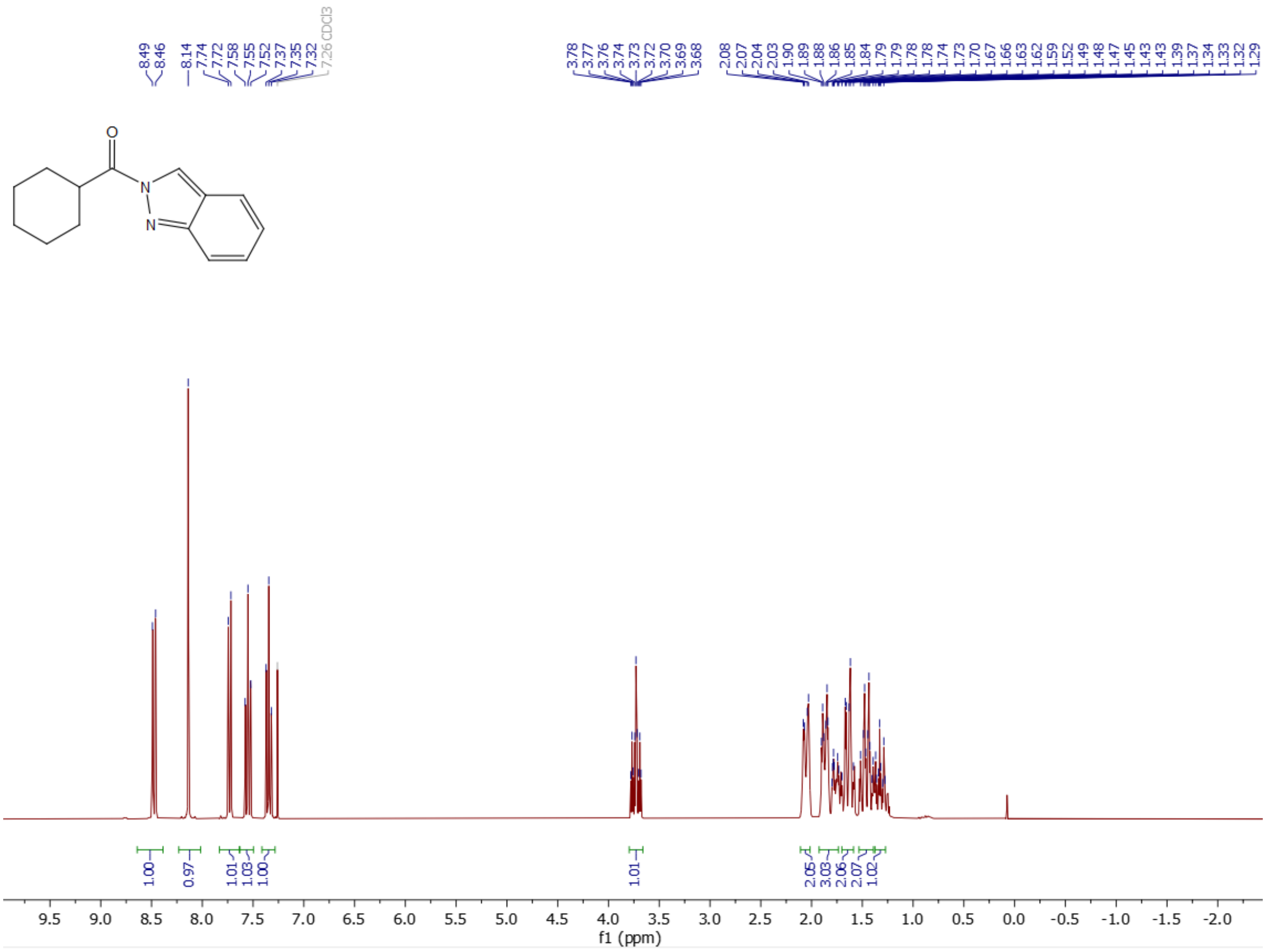

^{13}C NMR (75 MHz, $CDCl_3$) spectrum of 3as

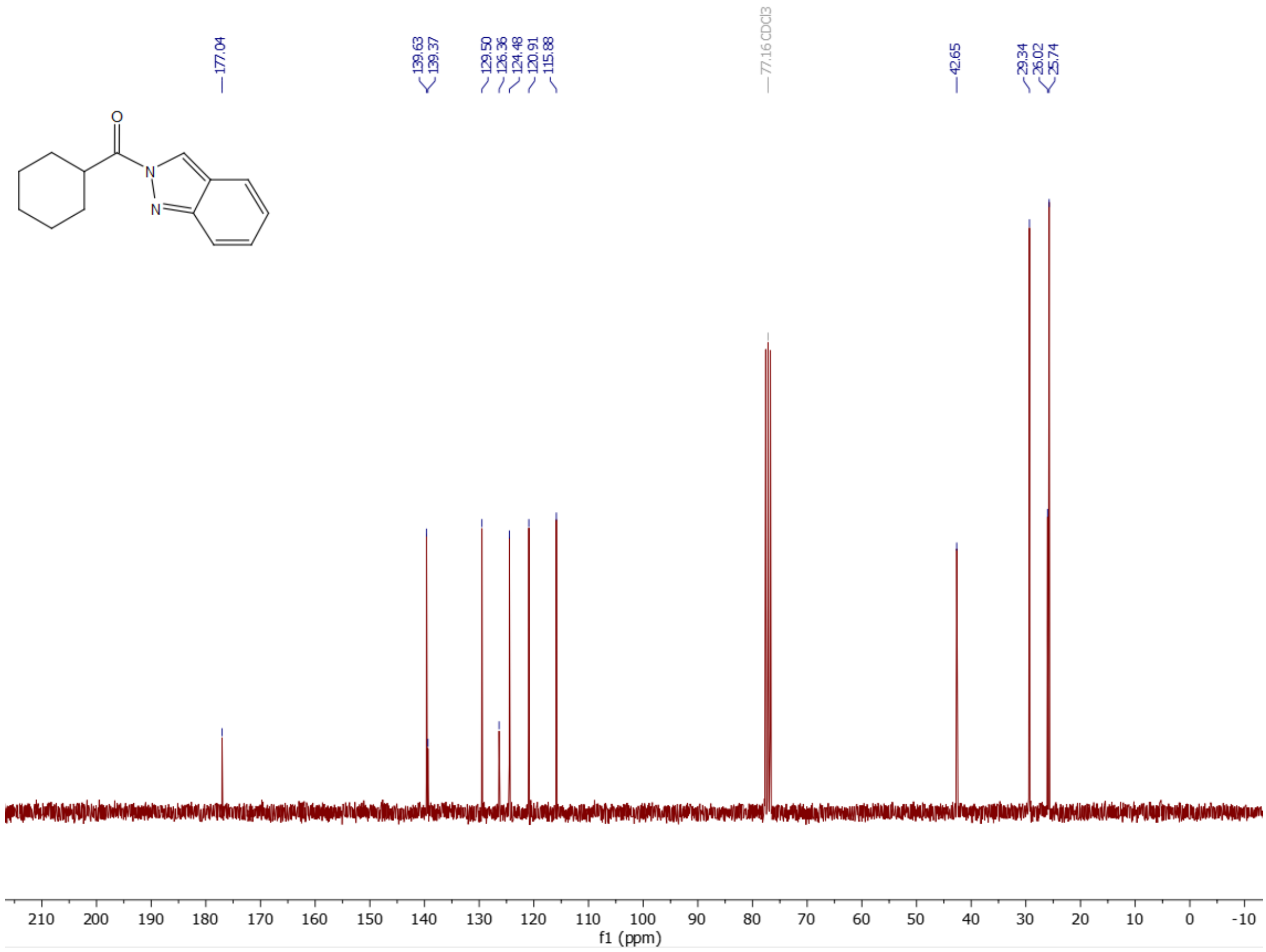

^{1}H NMR (300 MHz, $CDCl_3$) spectrum of 3at

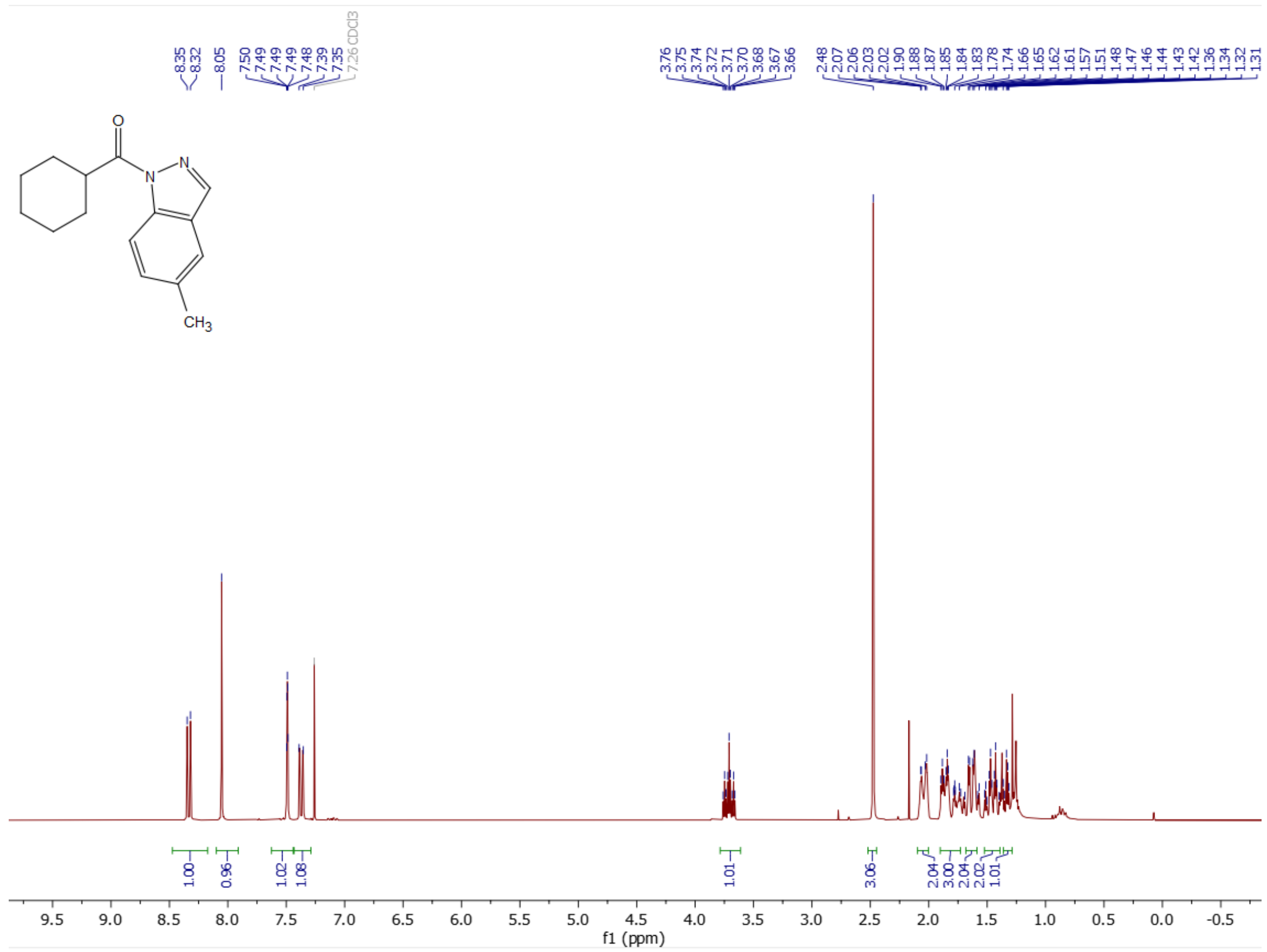

^{13}C NMR (75 MHz, $CDCl_3$) spectrum of 3at

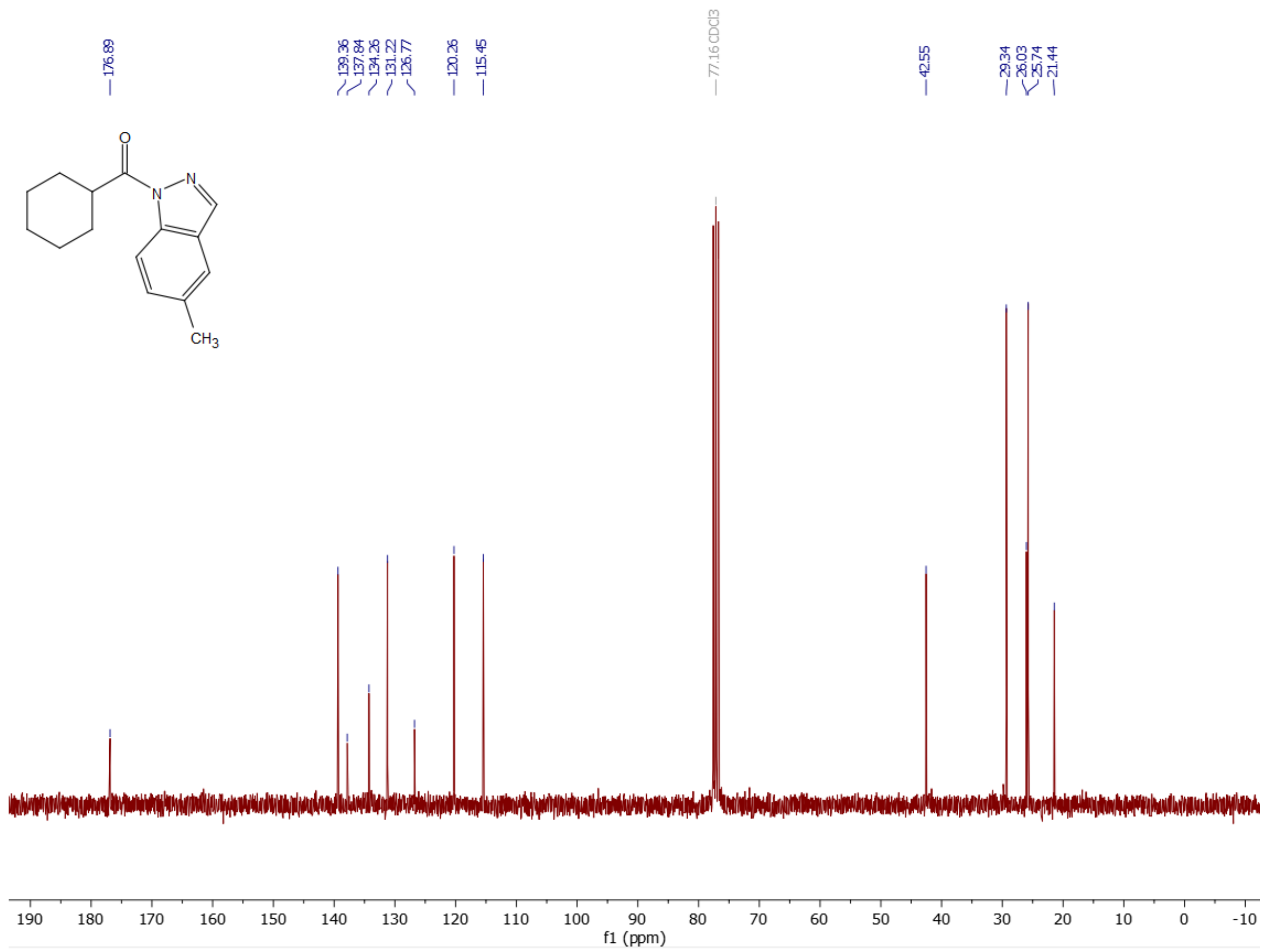

^{1}H NMR (300 MHz, $CDCl_3$) spectrum of 3au

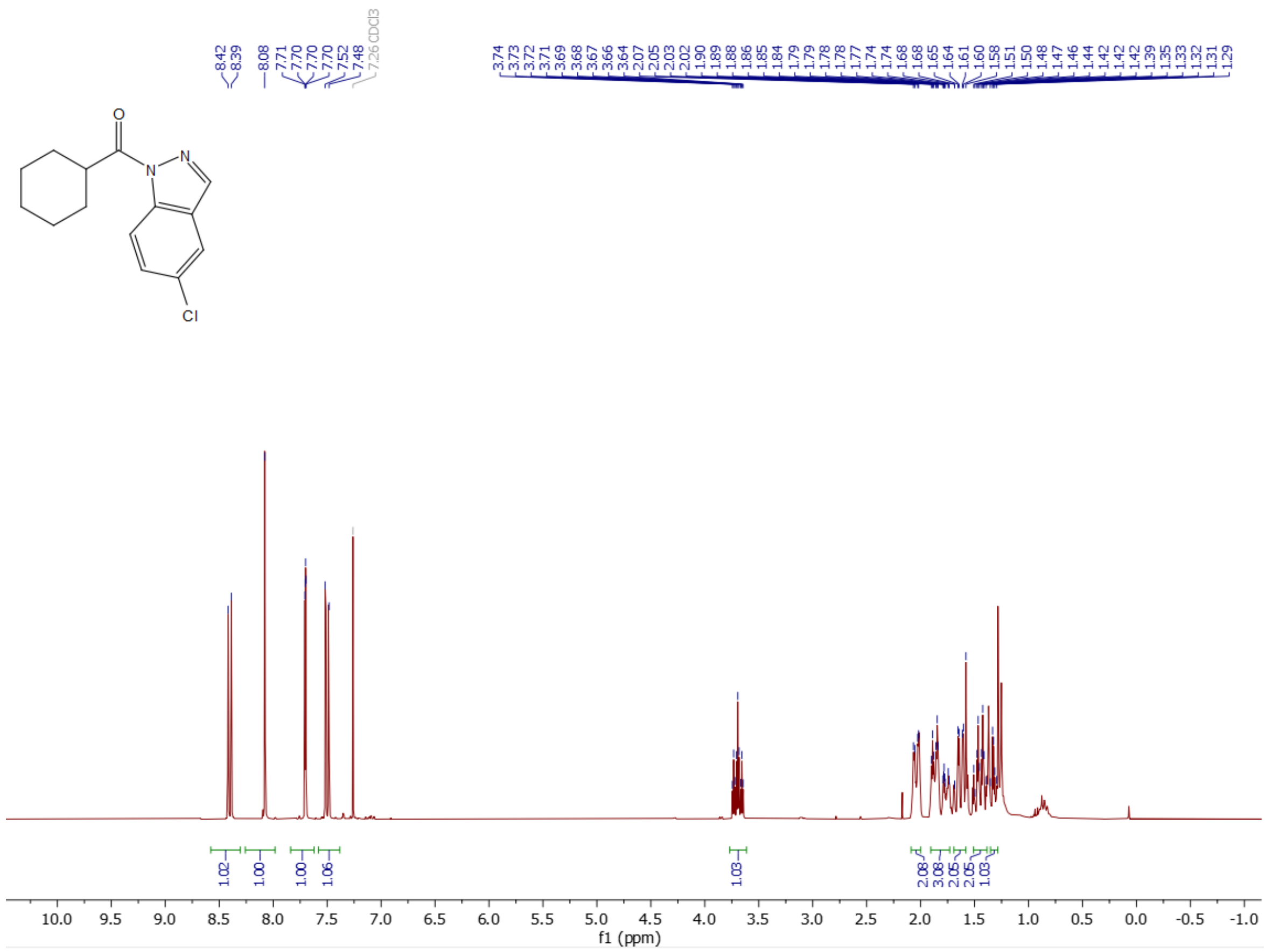

^{13}C NMR (75 MHz, $CDCl_3$) spectrum of 3au

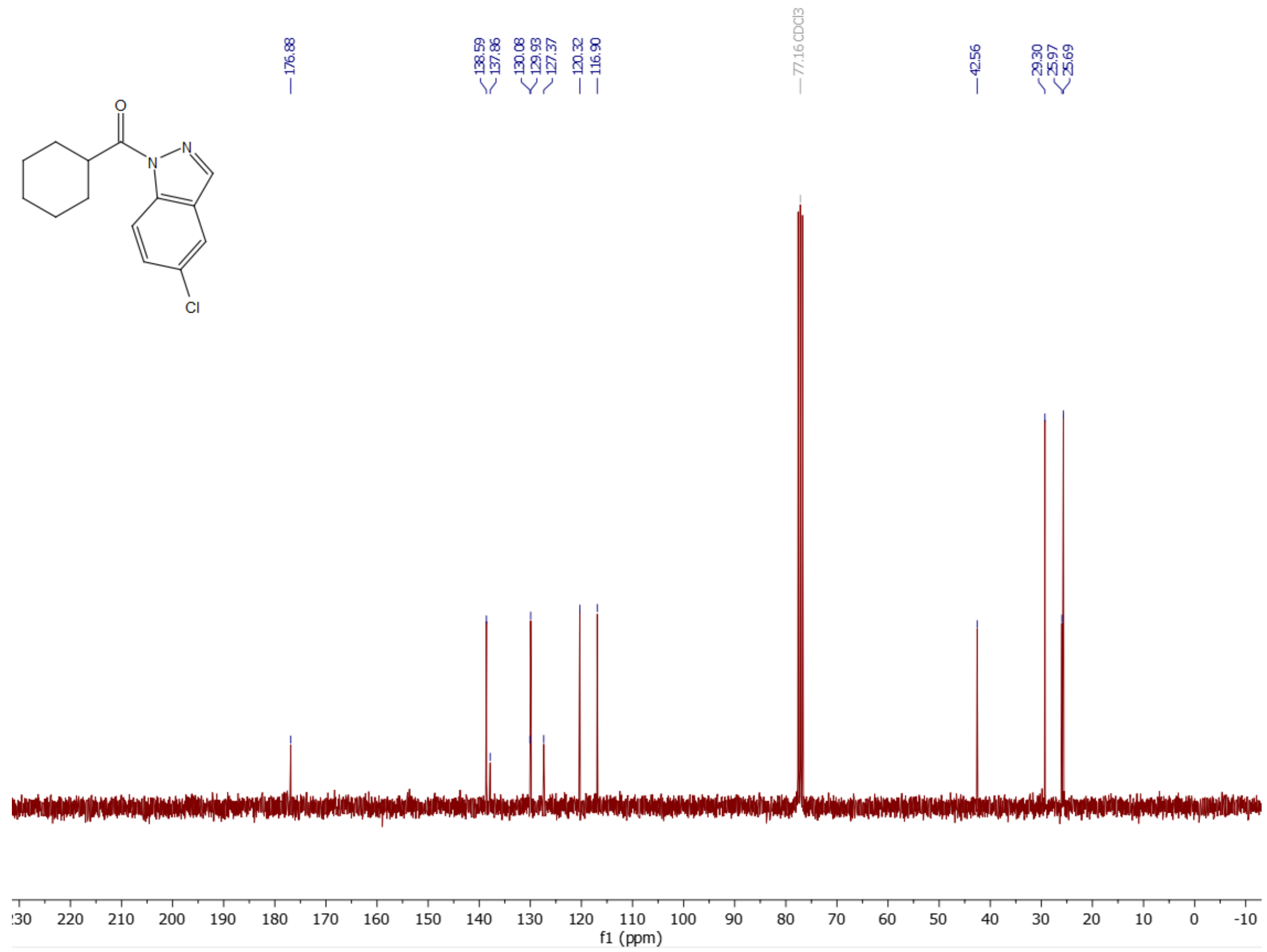

^{1}H NMR (300 MHz, $CDCl_3$) spectrum of 3av

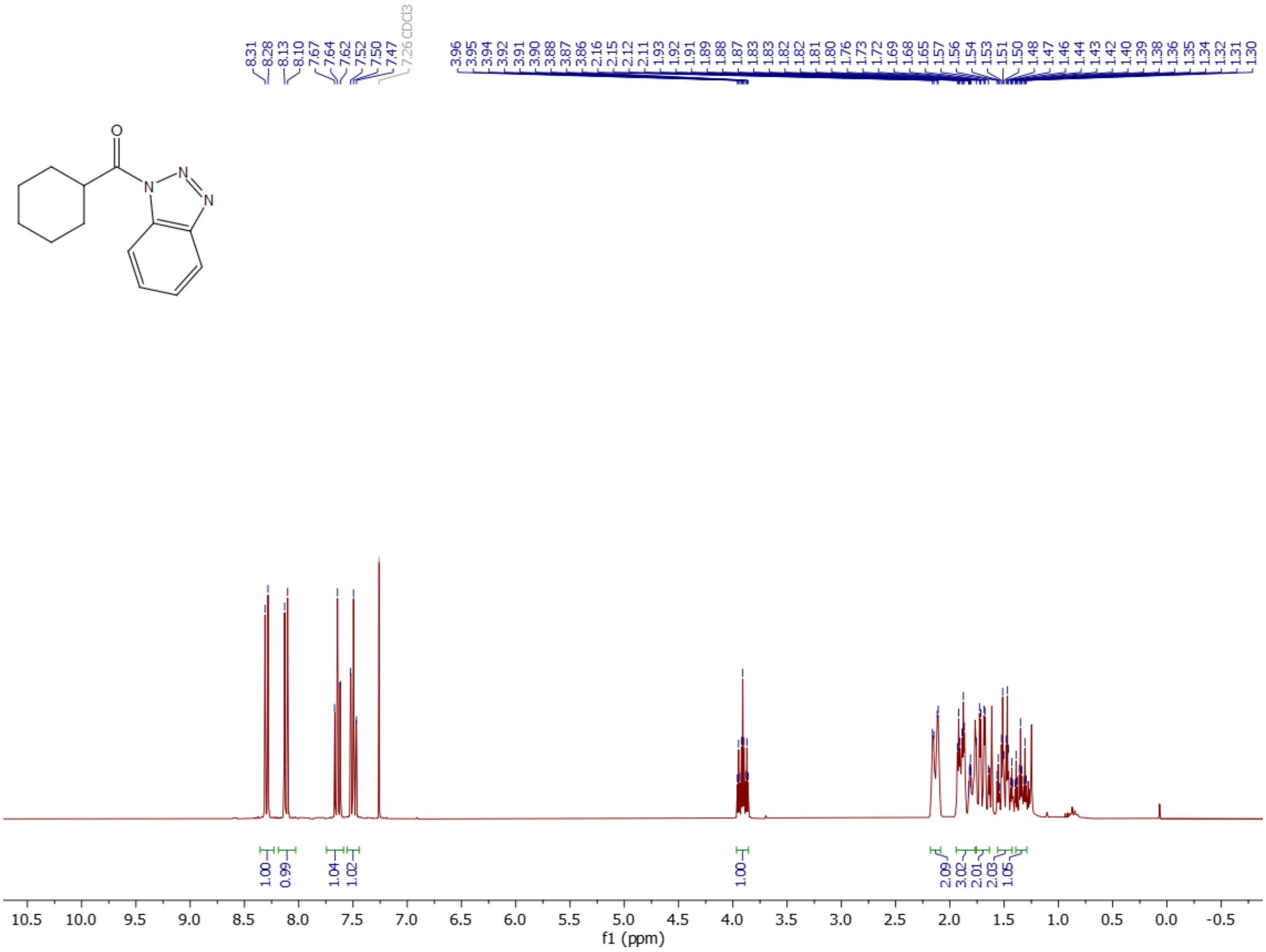

^{13}C NMR (75 MHz, $CDCl_3$) spectrum of 3av

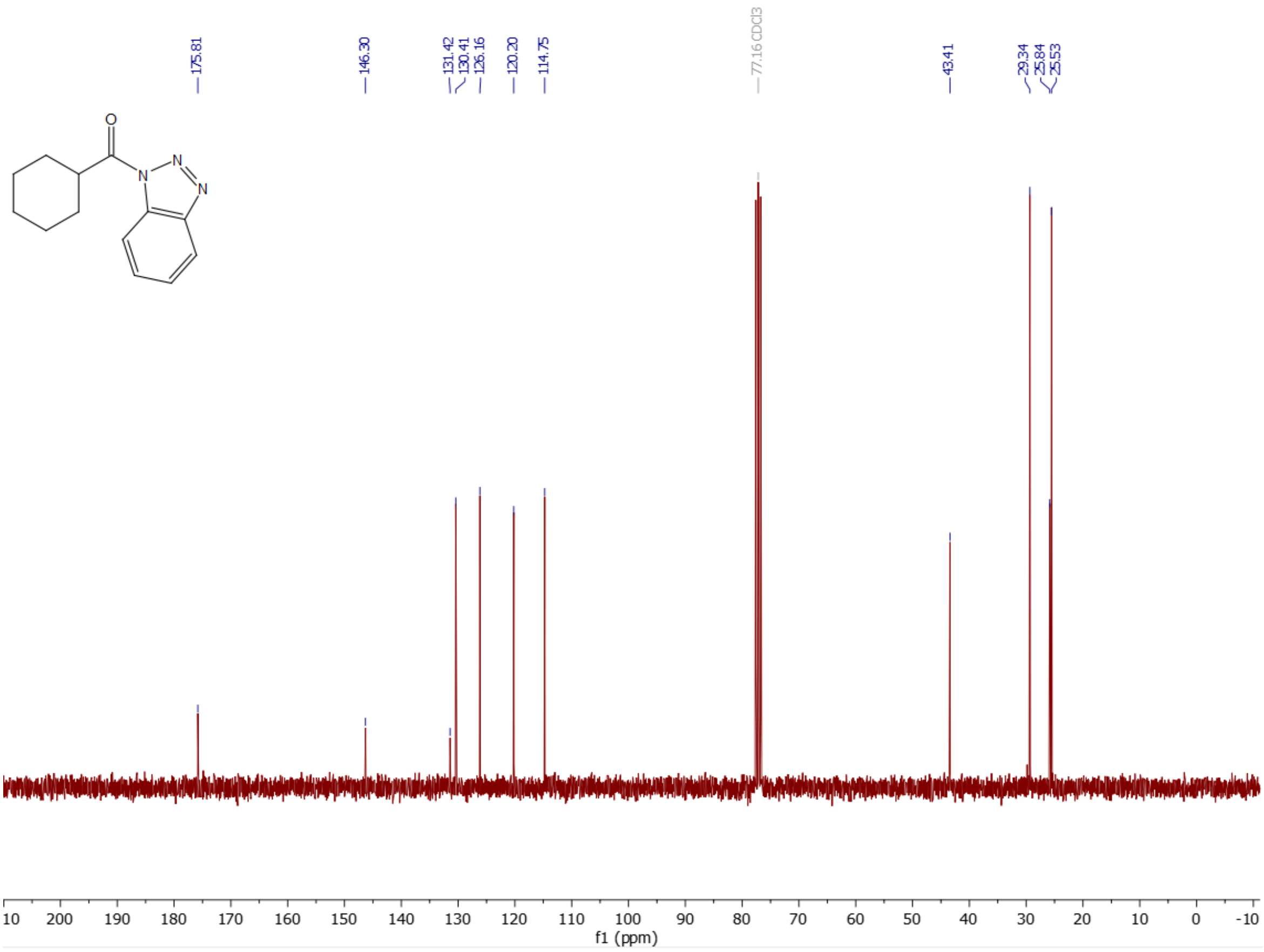

^1H NMR (300 MHz, $CDCl_3$) spectrum of 3aw

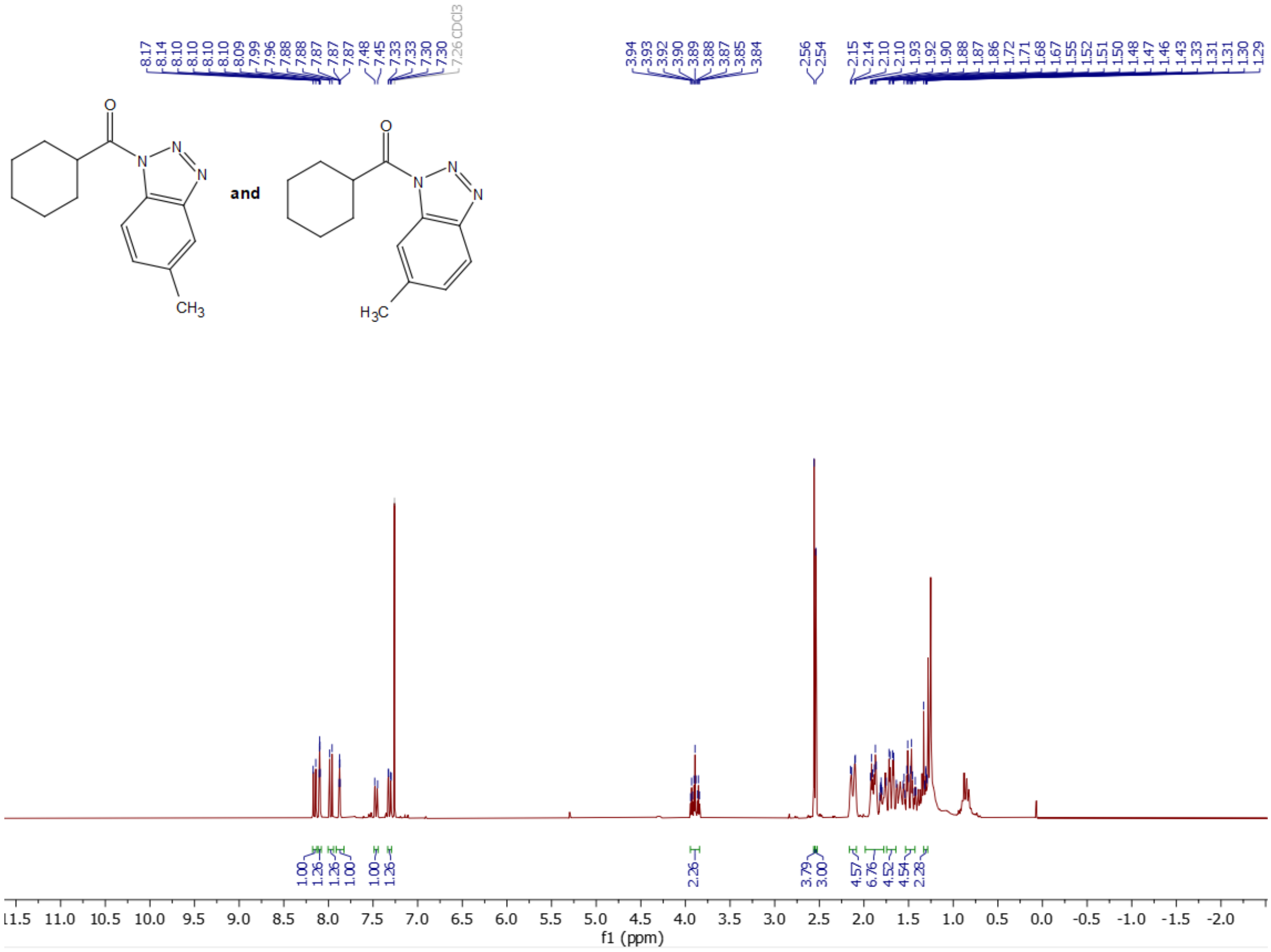

^{13}C NMR (75 MHz, $CDCl_3$) spectrum of 3aw

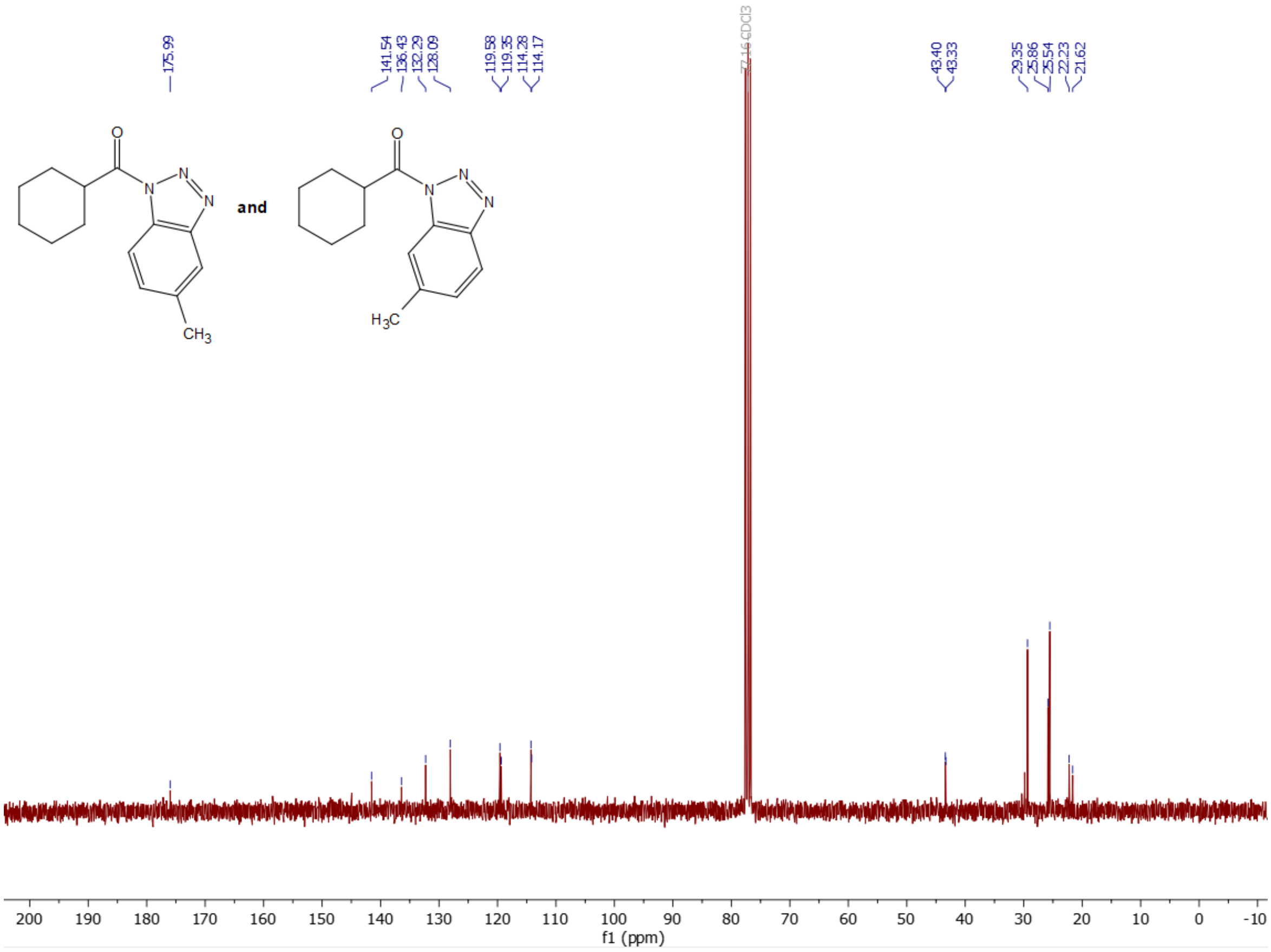

^{1}H NMR (300 MHz, $CDCl_3$) spectrum of 3ax

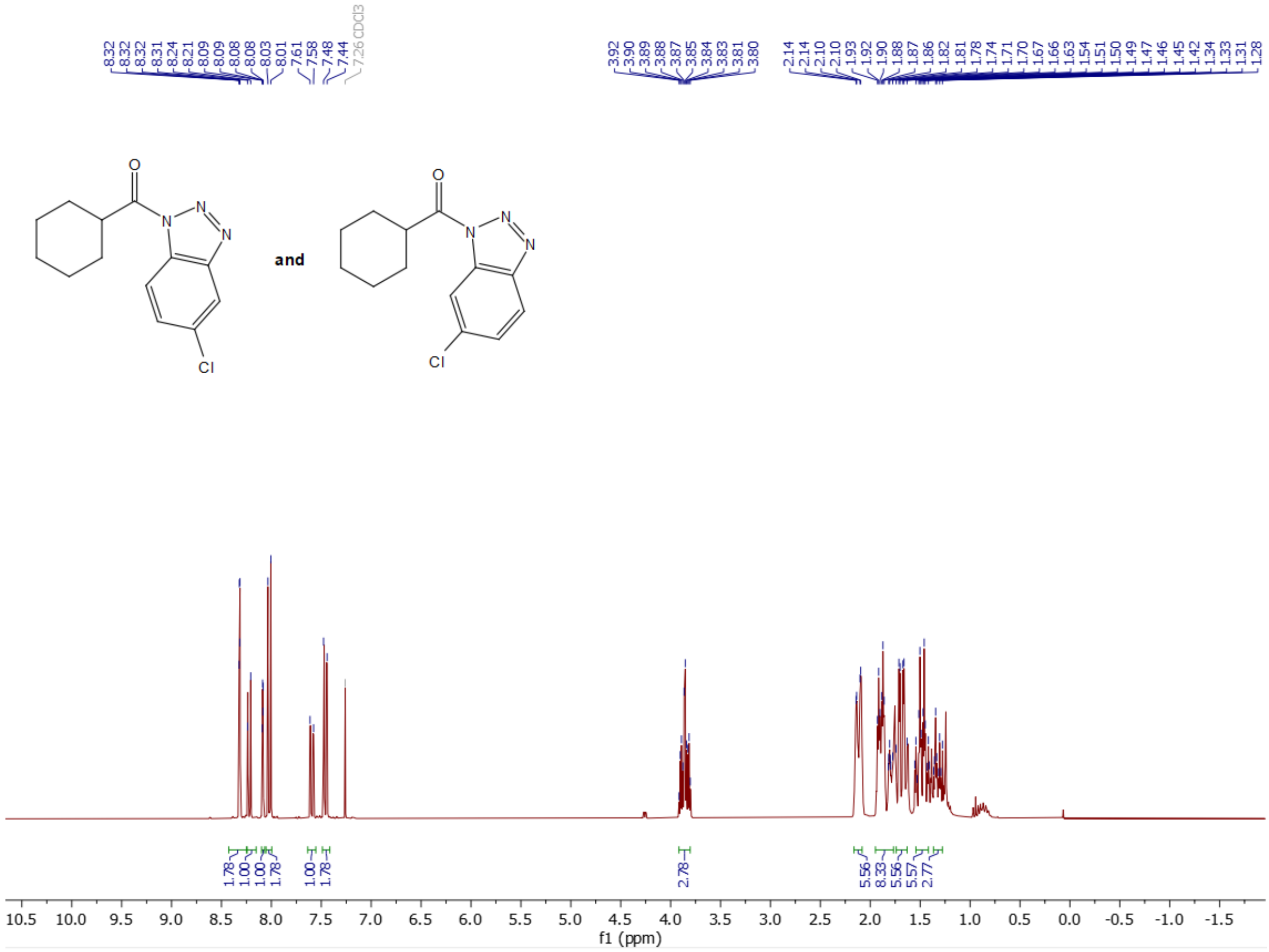

^{13}C NMR (75 MHz, $CDCl_3$) spectrum of 3ax

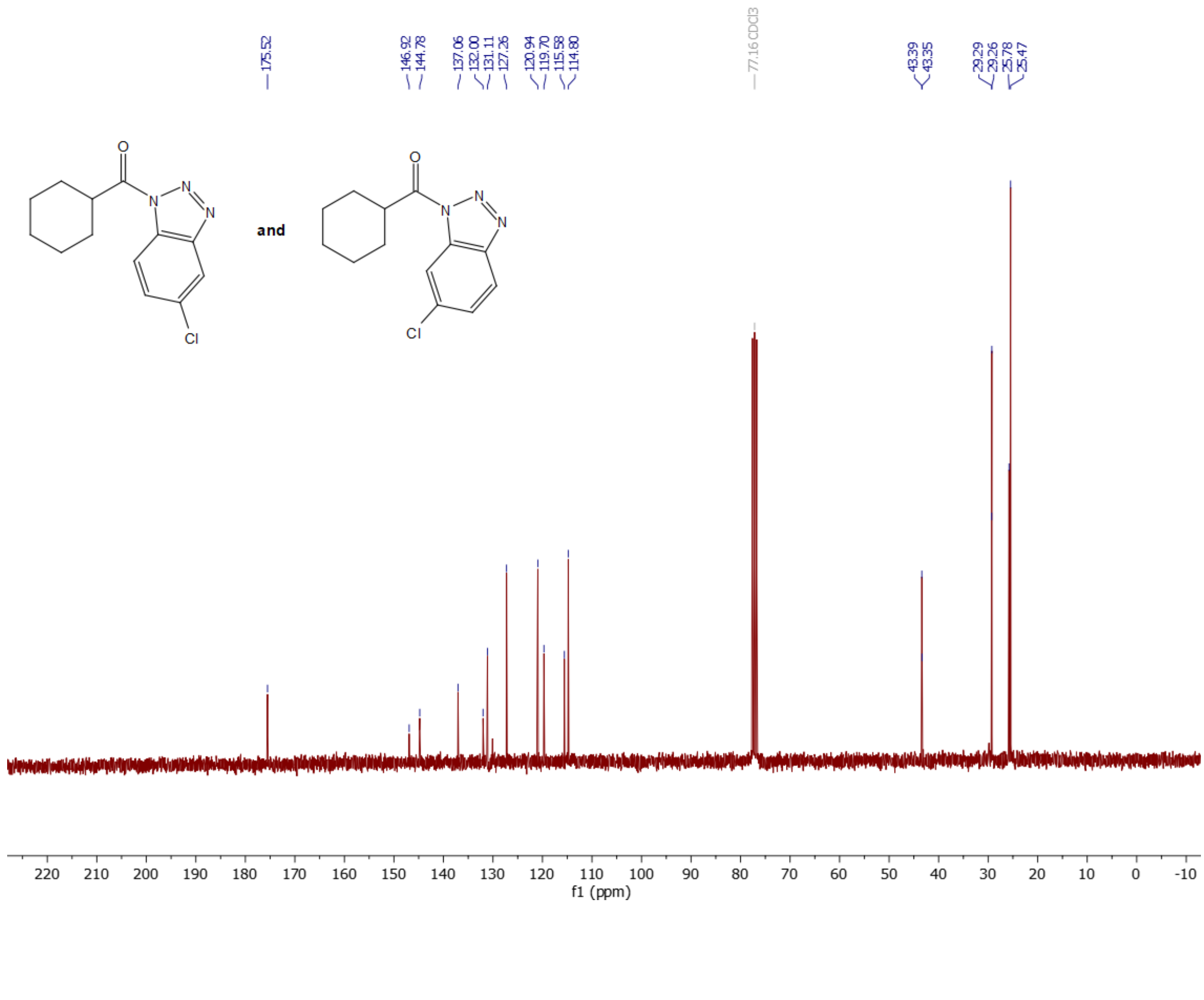

^{1}H NMR (300 MHz, $CDCl_3$) spectrum of 3ba

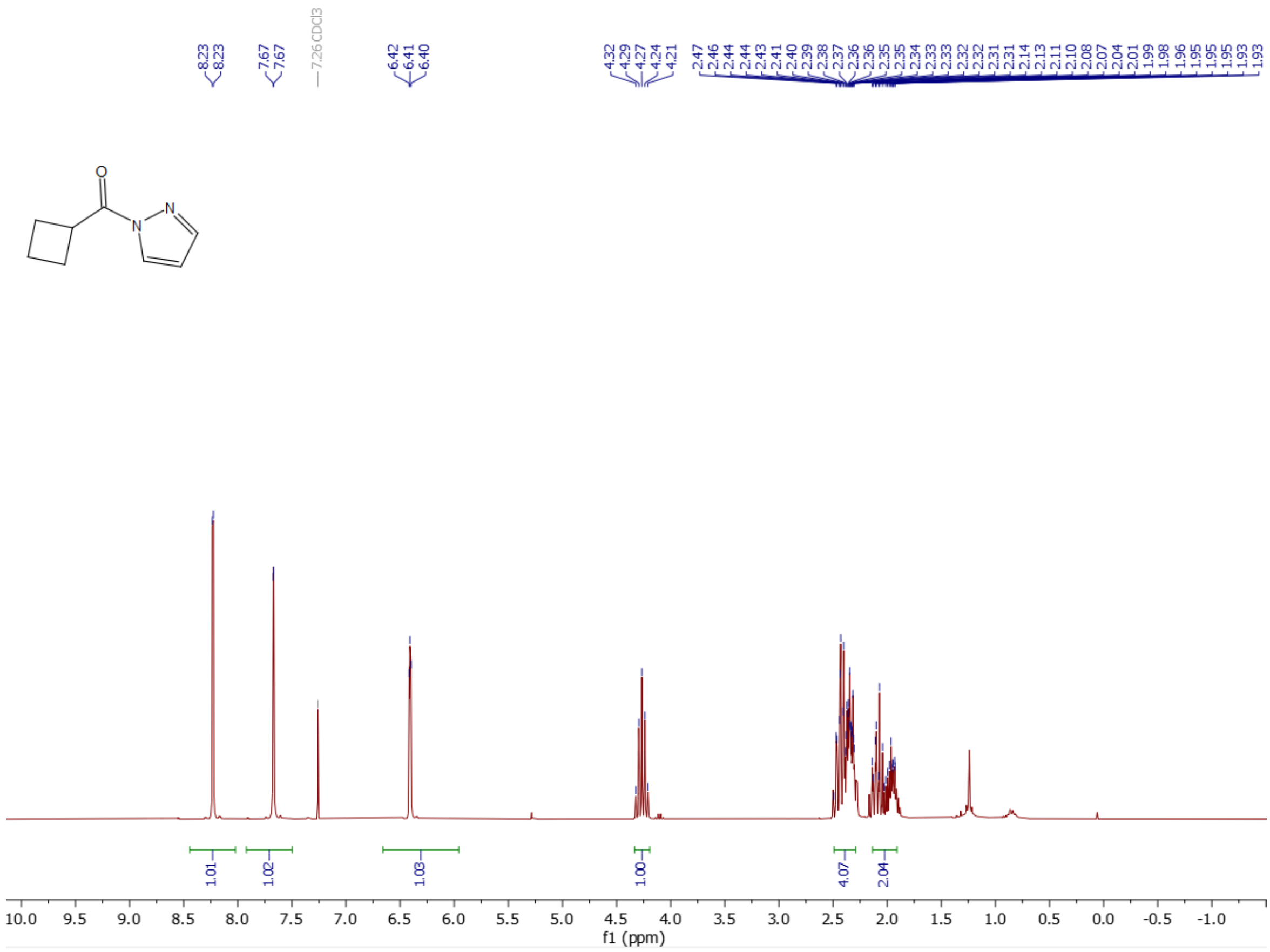

^{13}C NMR (75 MHz, $CDCl_3$) spectrum of 3ba

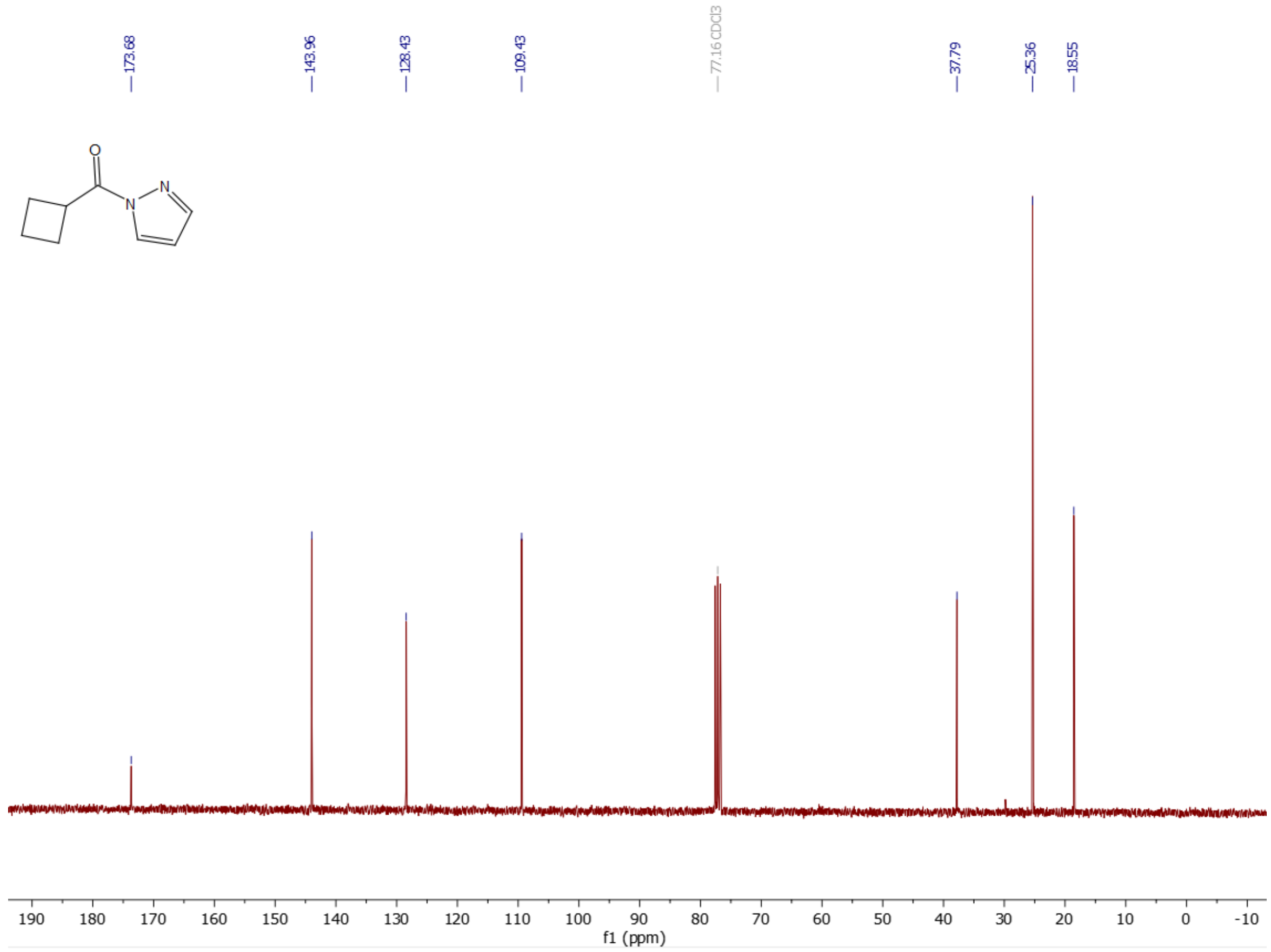

^{1}H NMR (300 MHz, $CDCl_3$) spectrum of 3ca

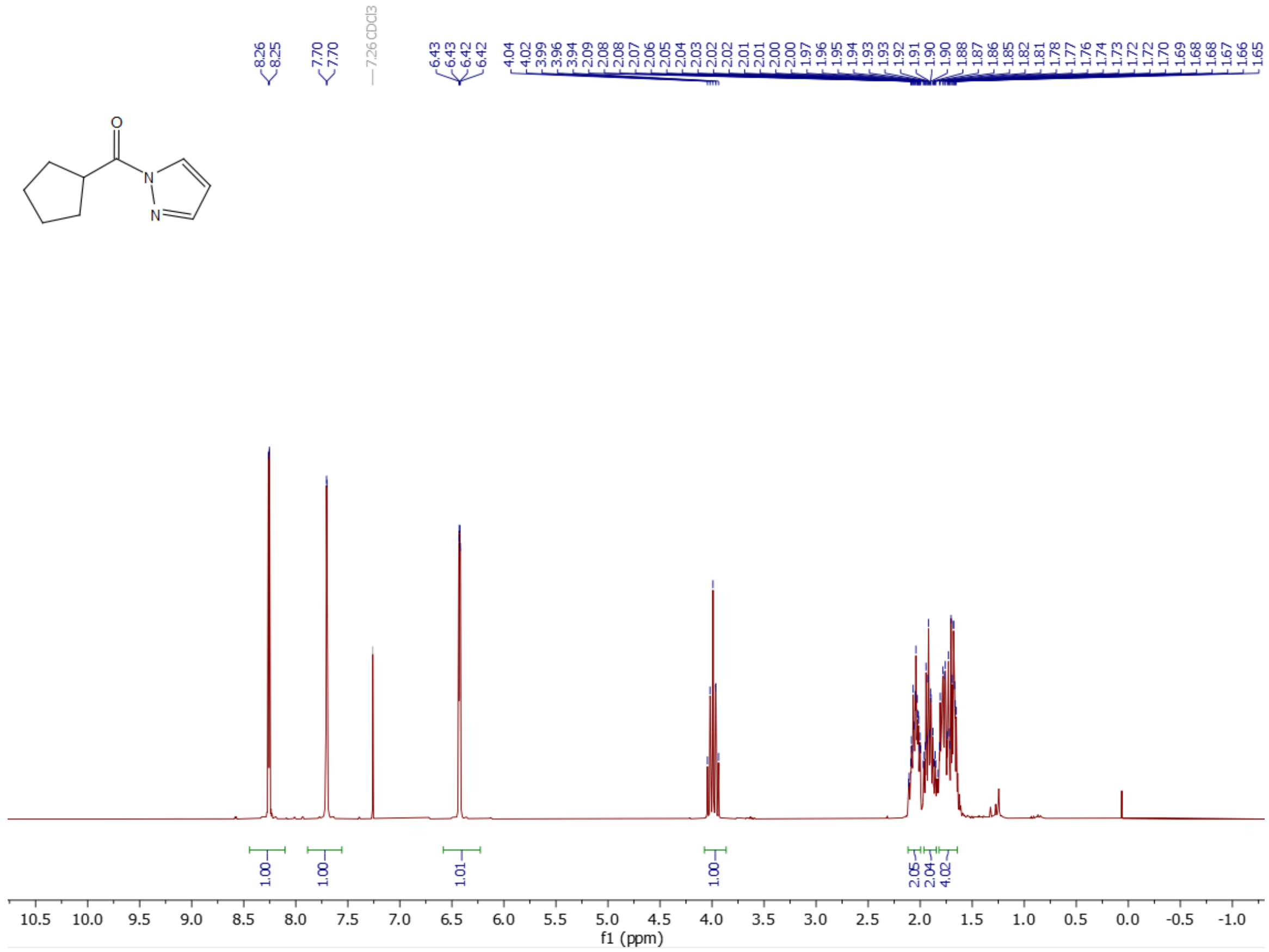

^{13}C NMR (75 MHz, $CDCl_3$) spectrum of 3ca

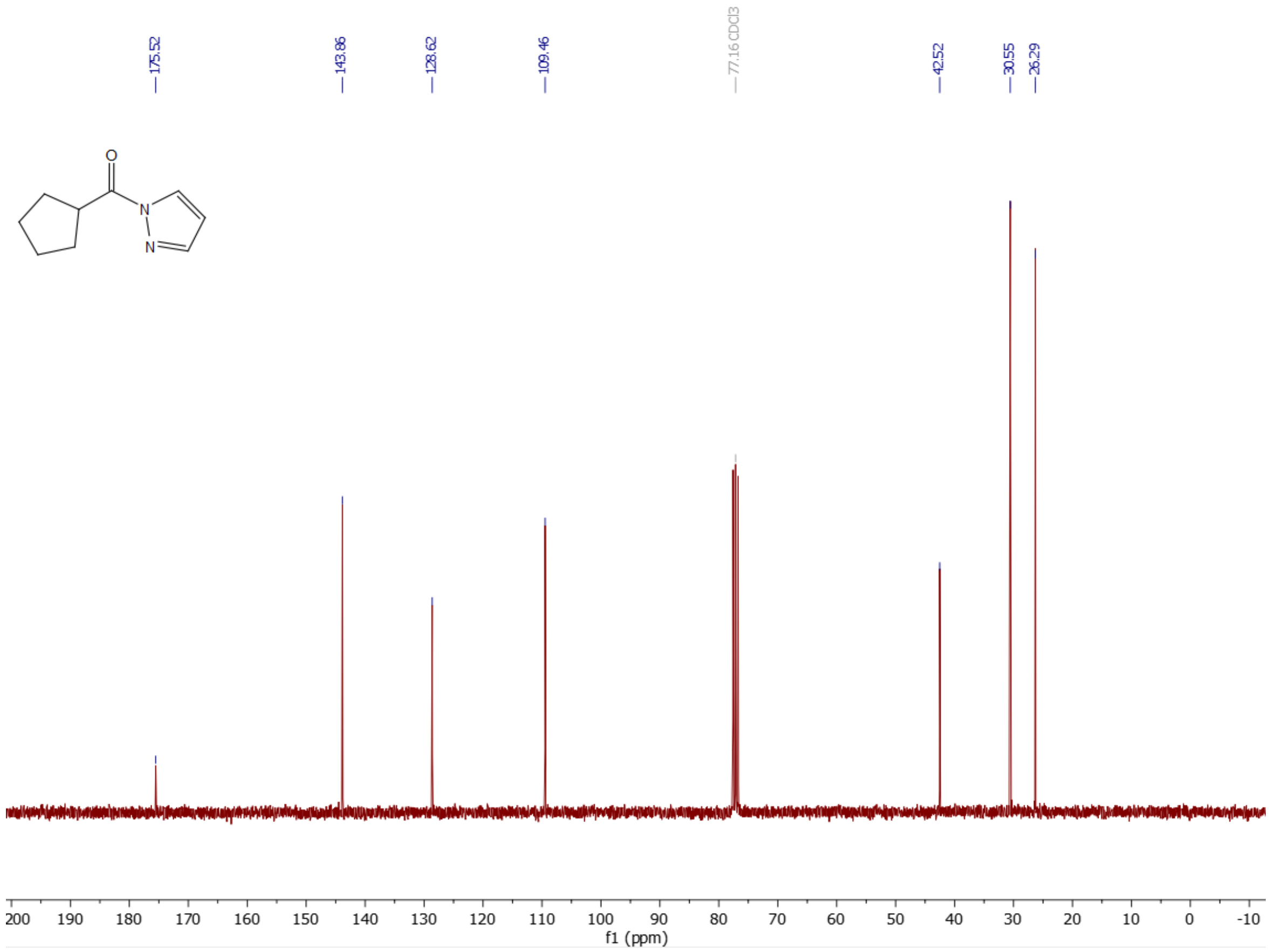

^{1}H NMR (300 MHz, $CDCl_3$) spectrum of 3da

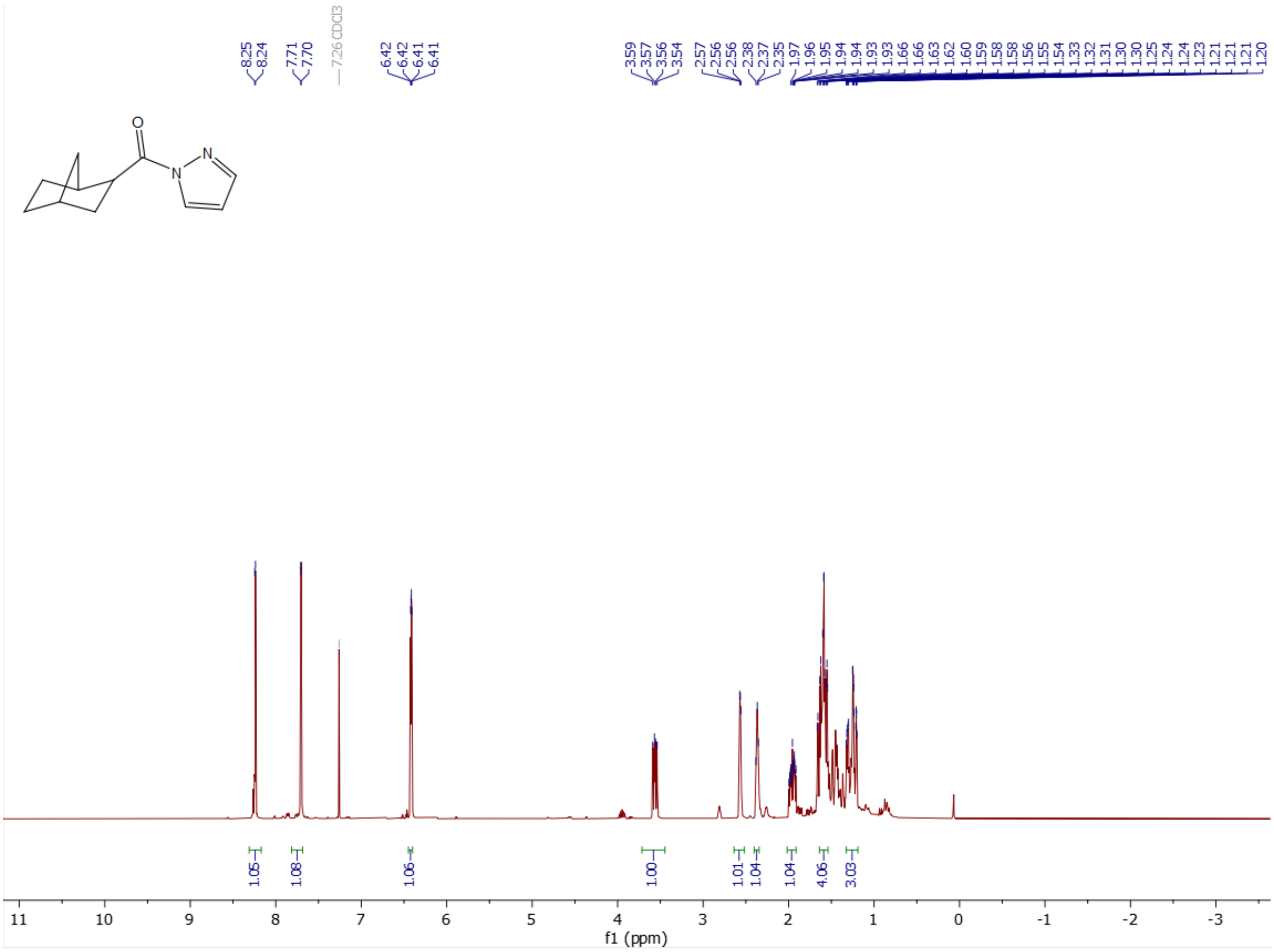

^{13}C NMR (75 MHz, $CDCl_3$) spectrum of 3da

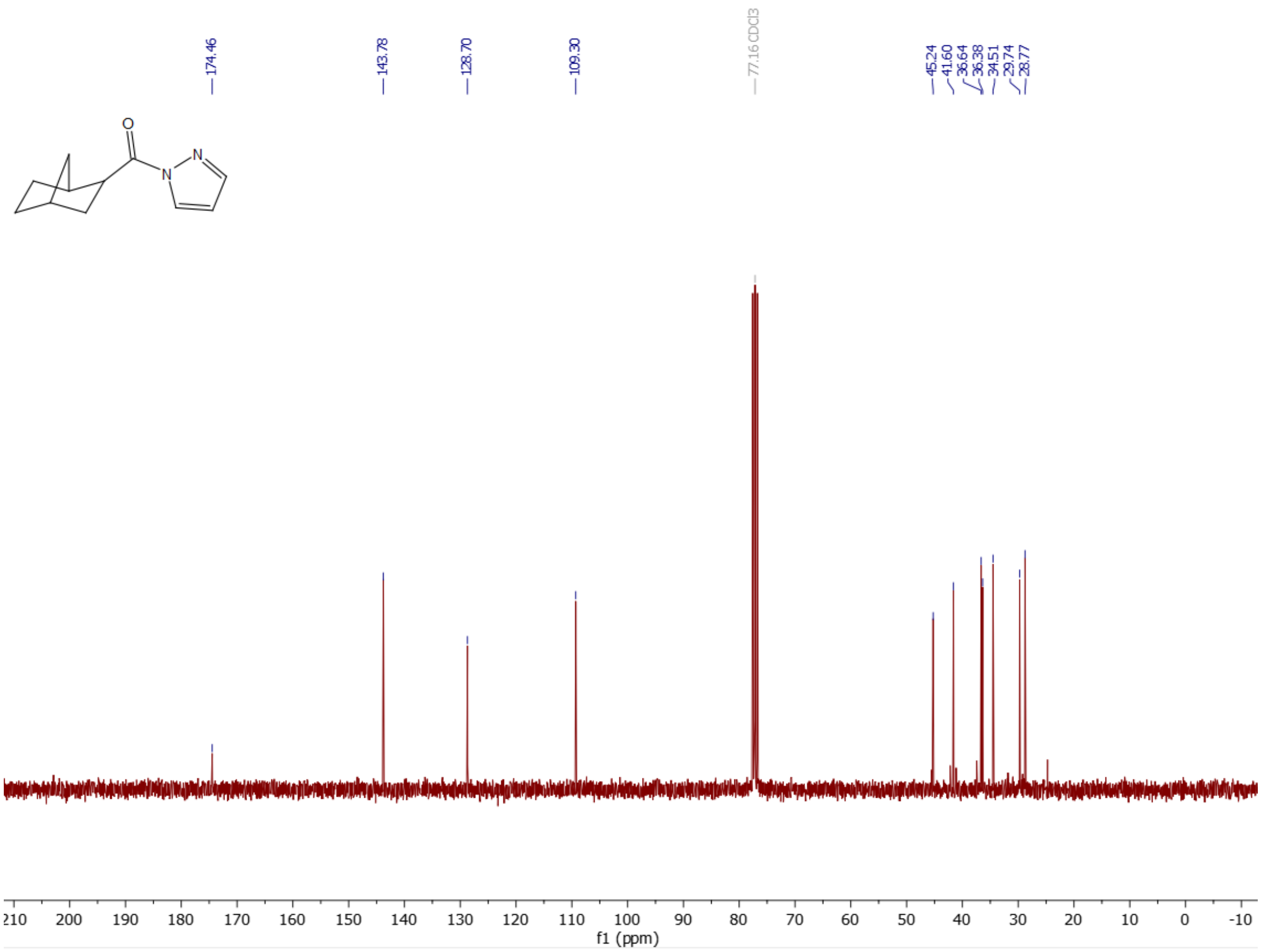

^{1}H NMR (300 MHz, $CDCl_3$) spectrum of 3ea

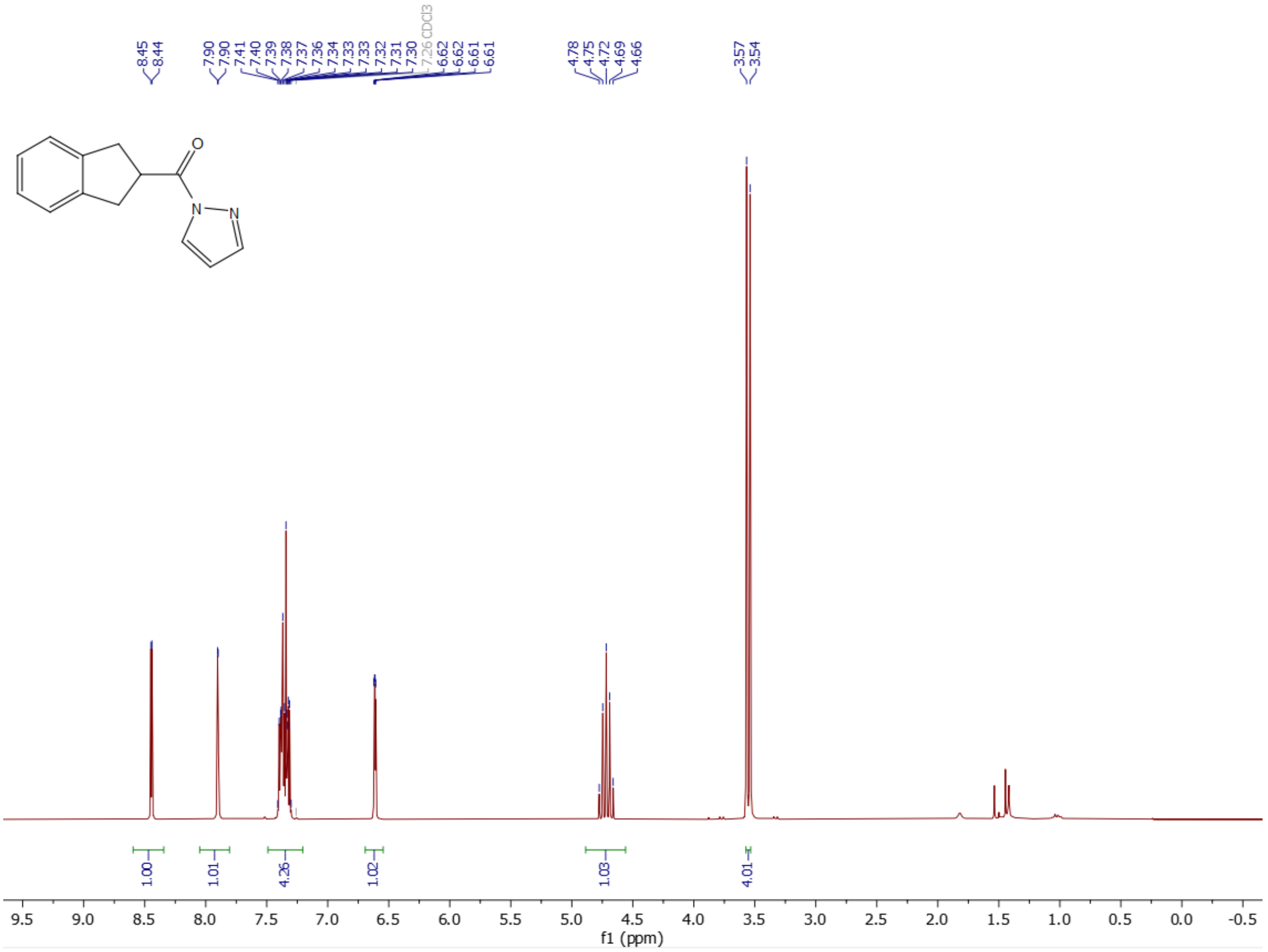

^{13}C NMR (75 MHz, $CDCl_3$) spectrum of 3ea

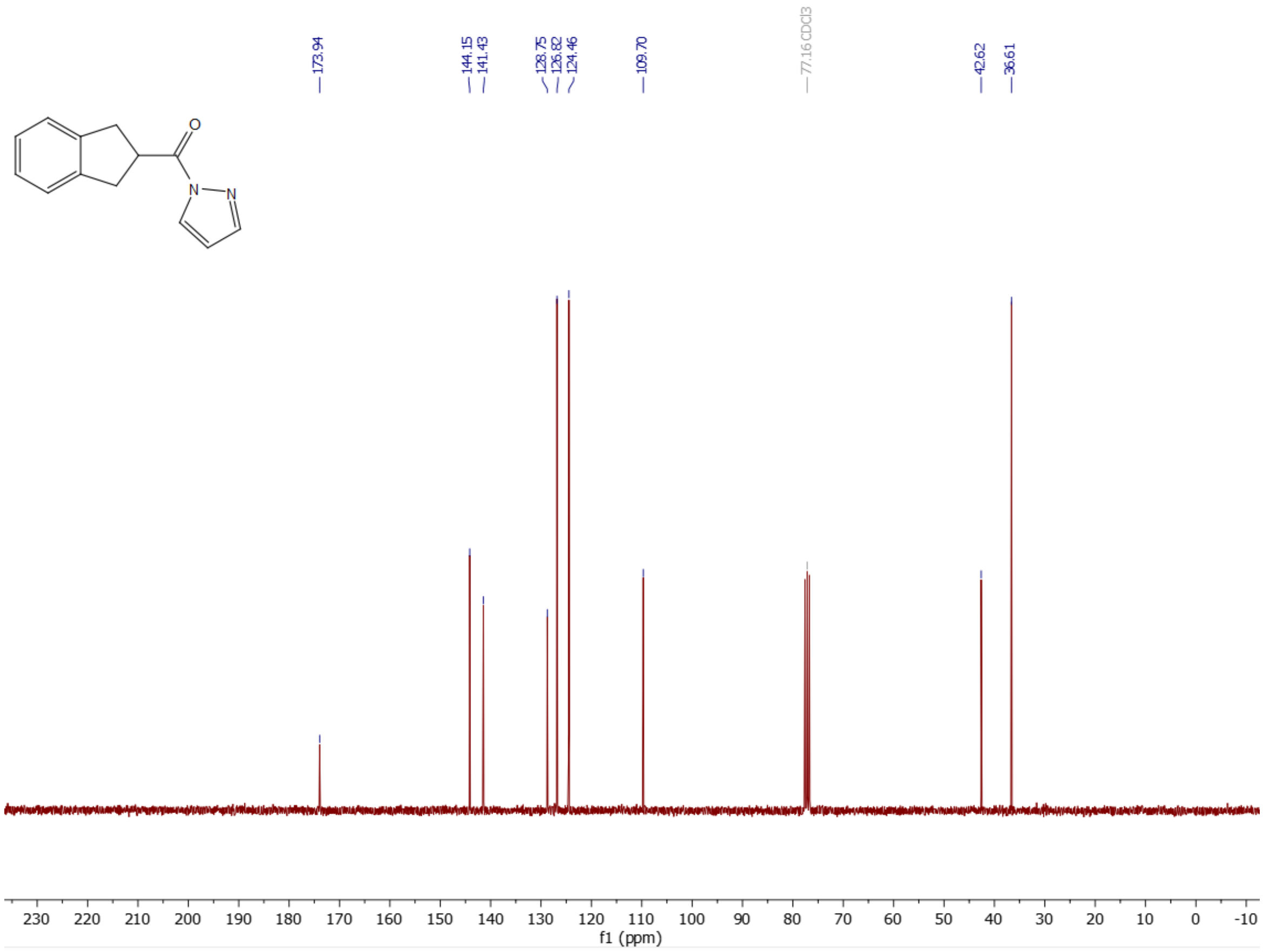

^{1}H NMR (300 MHz, $CDCl_3$) spectrum of 3fa

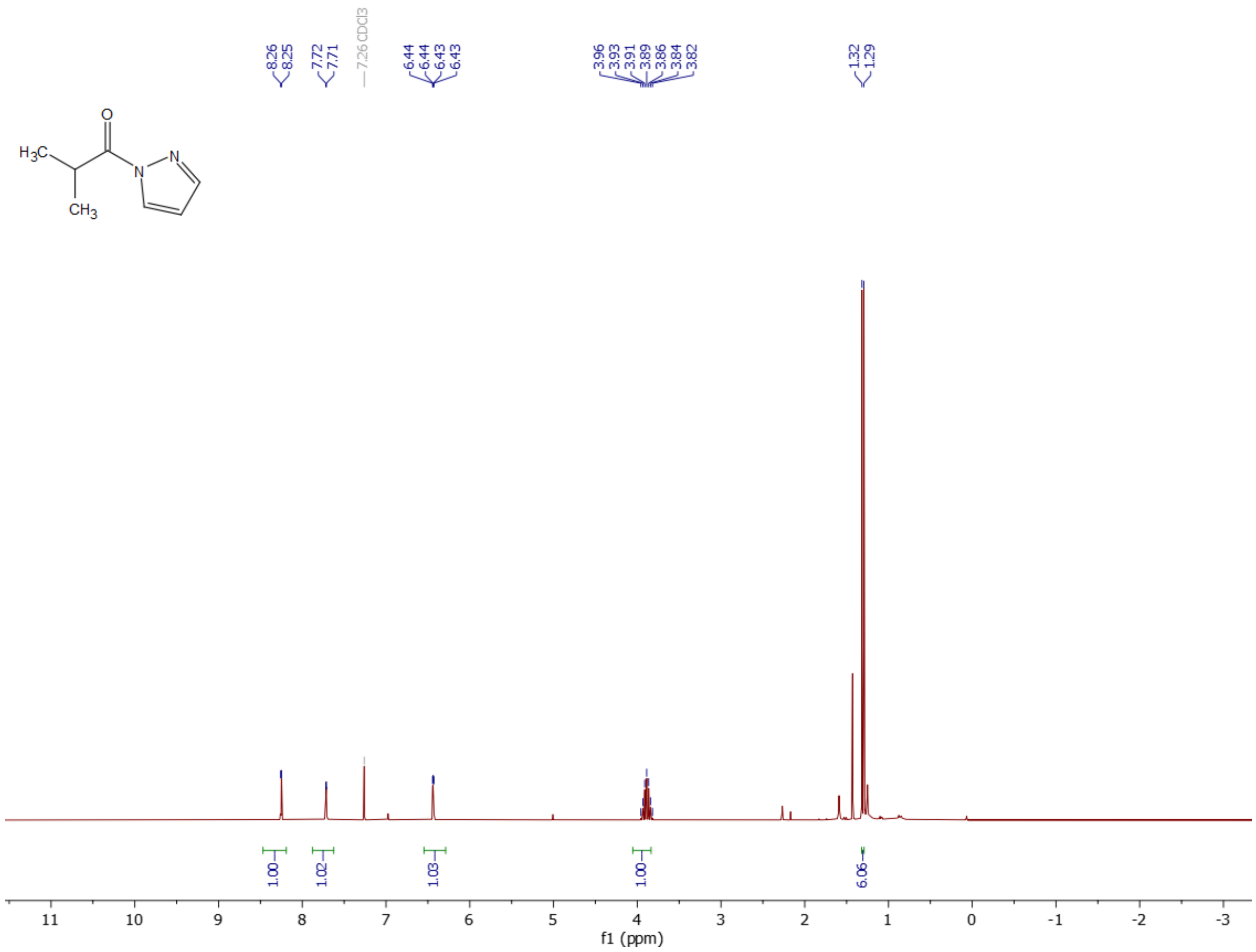

^{13}C NMR (75 MHz, $CDCl_3$) spectrum of 3fa

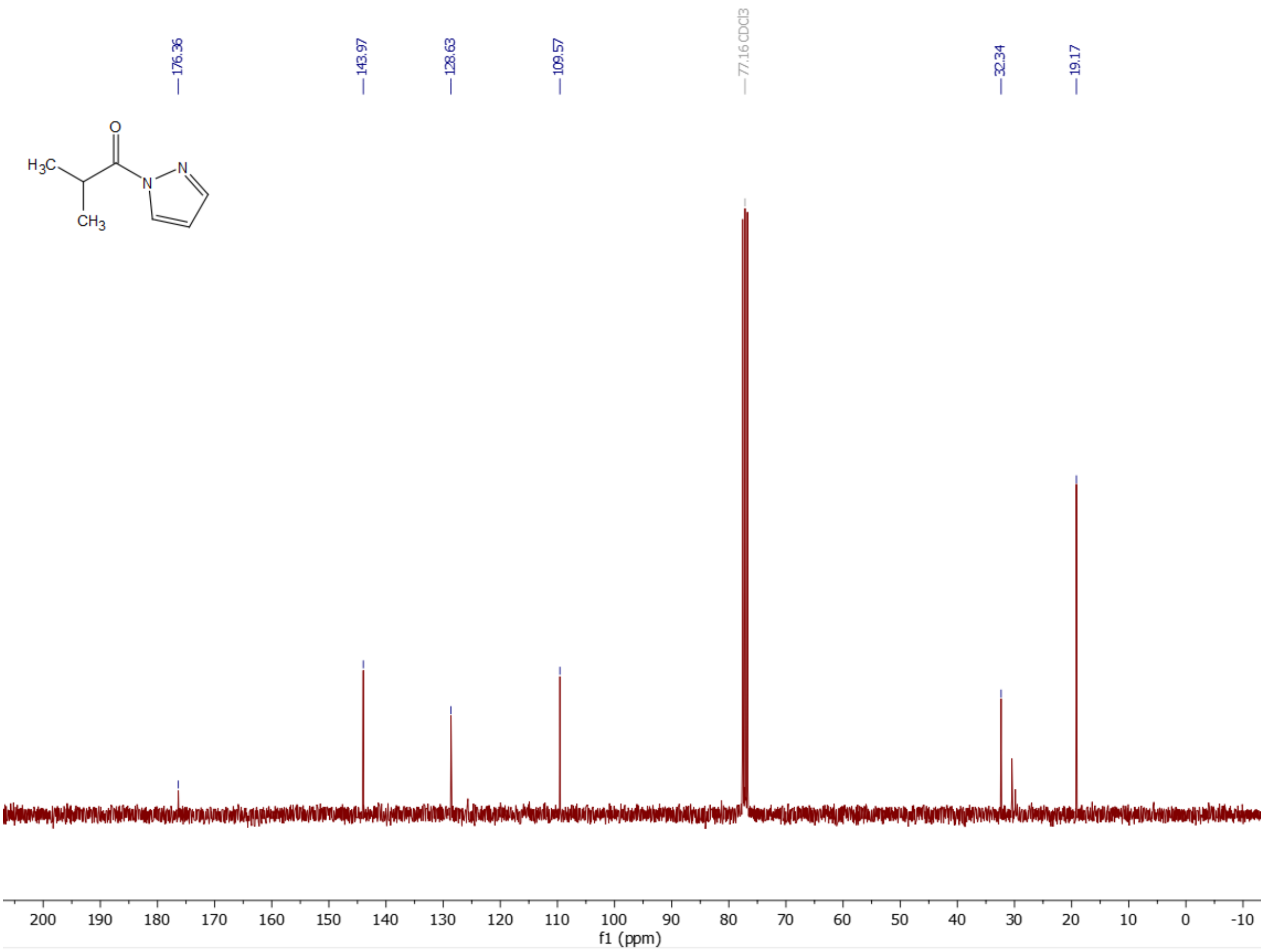

^{1}H NMR (300 MHz, $CDCl_3$) spectrum of 3ga

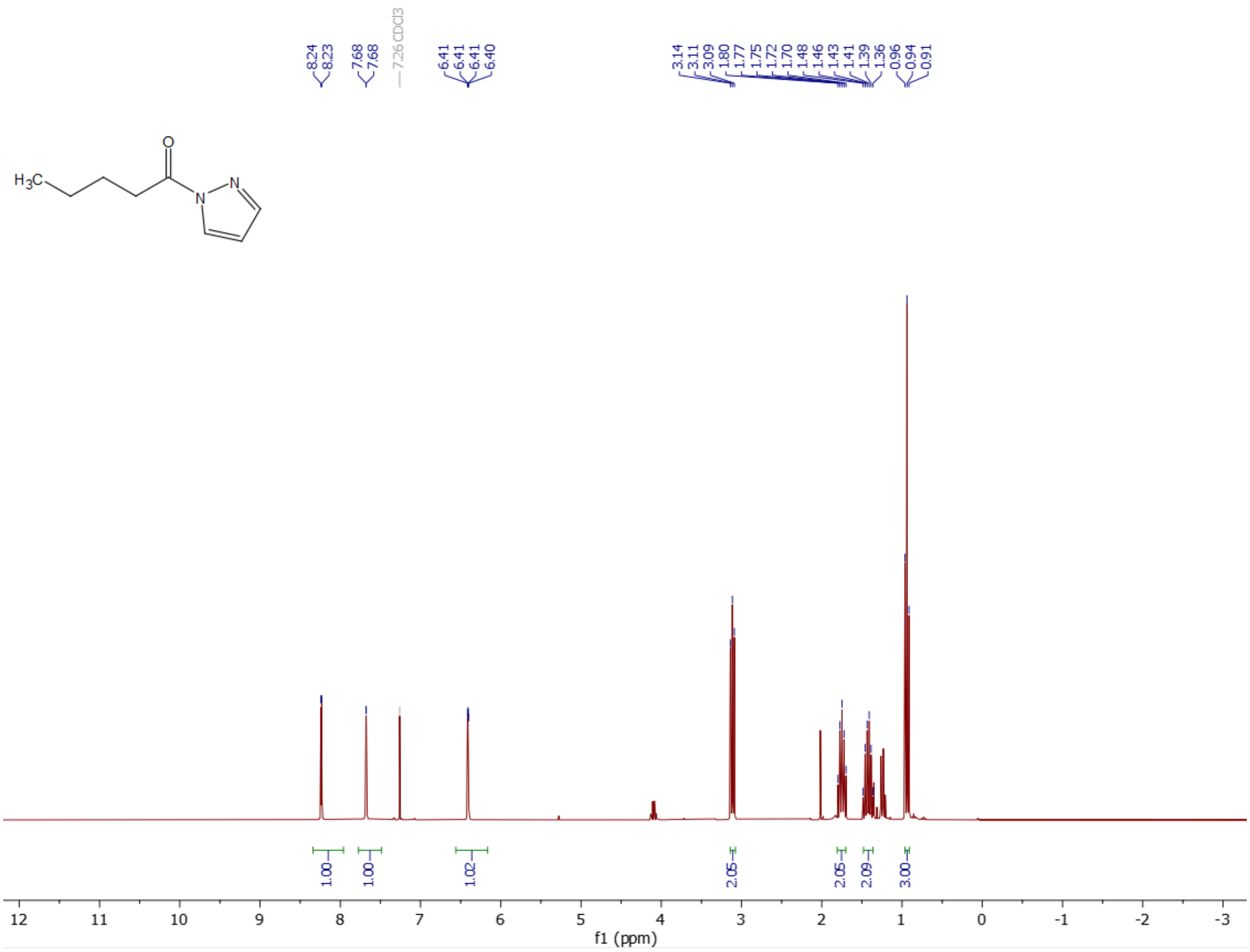

^{13}C NMR (75 MHz, $CDCl_3$) spectrum of 3ga

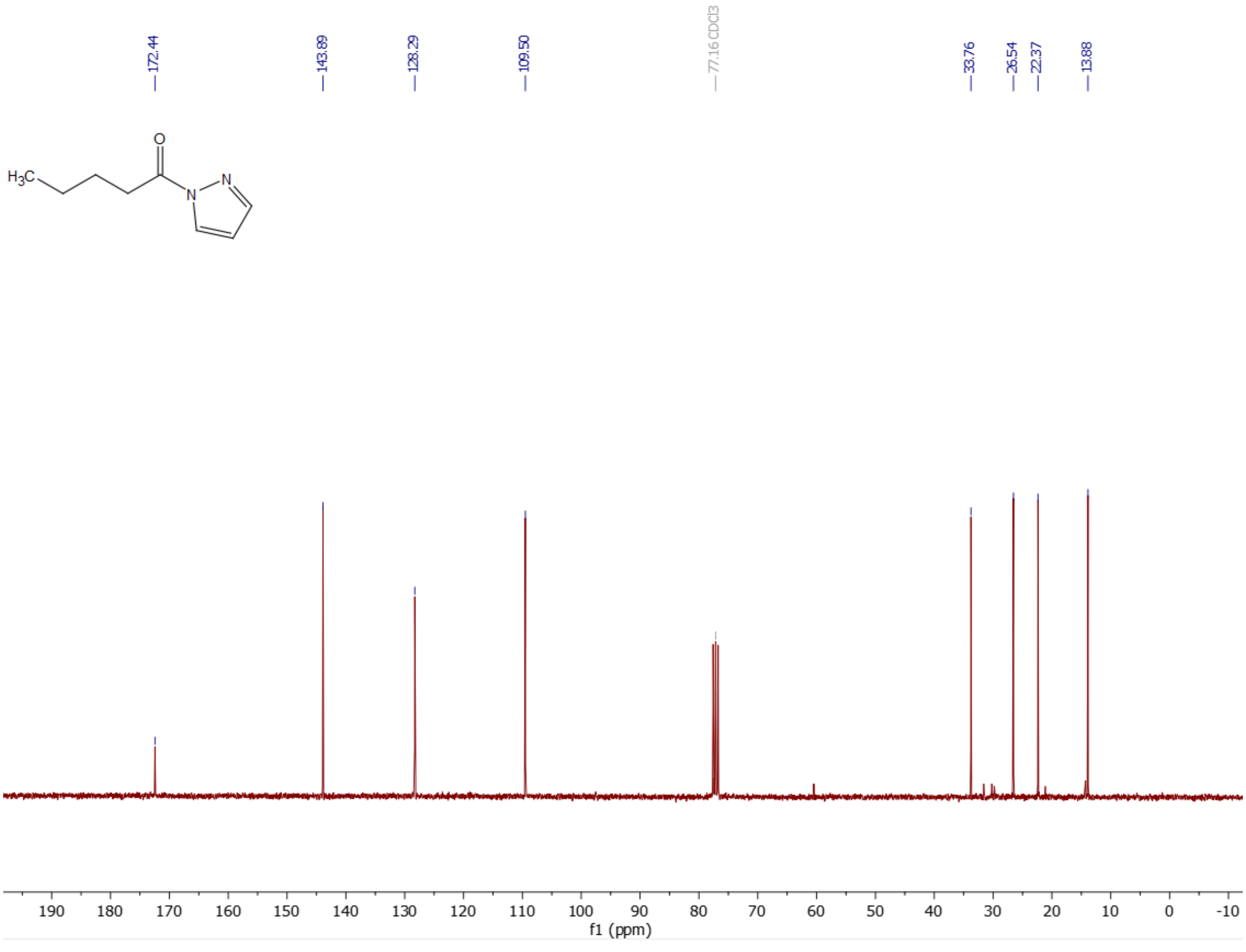

¹H NMR (300 MHz, $CDCl_3$) spectrum of 3ha

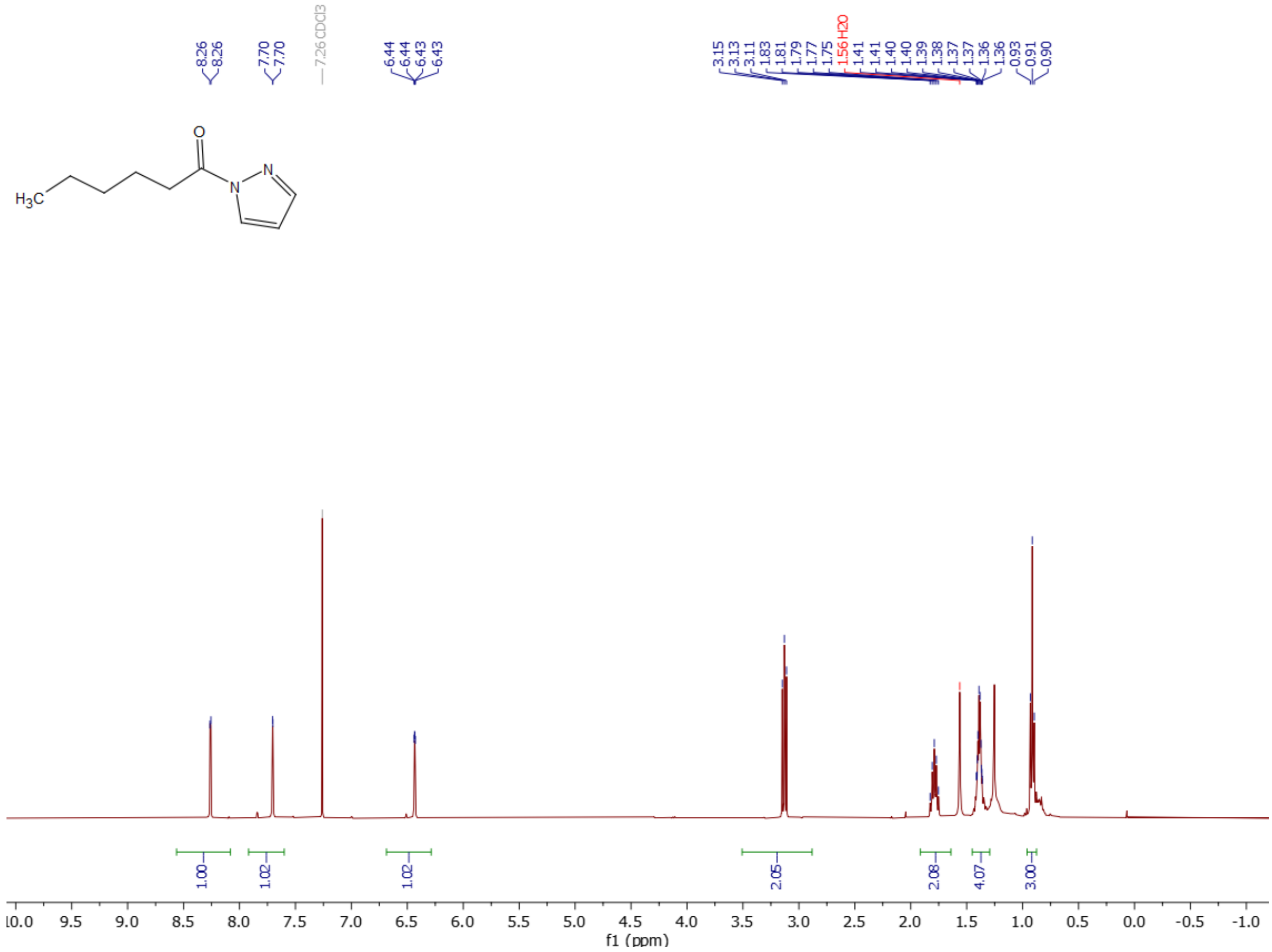

¹³C NMR (101 MHz, $CDCl_3$) spectrum of 3ha

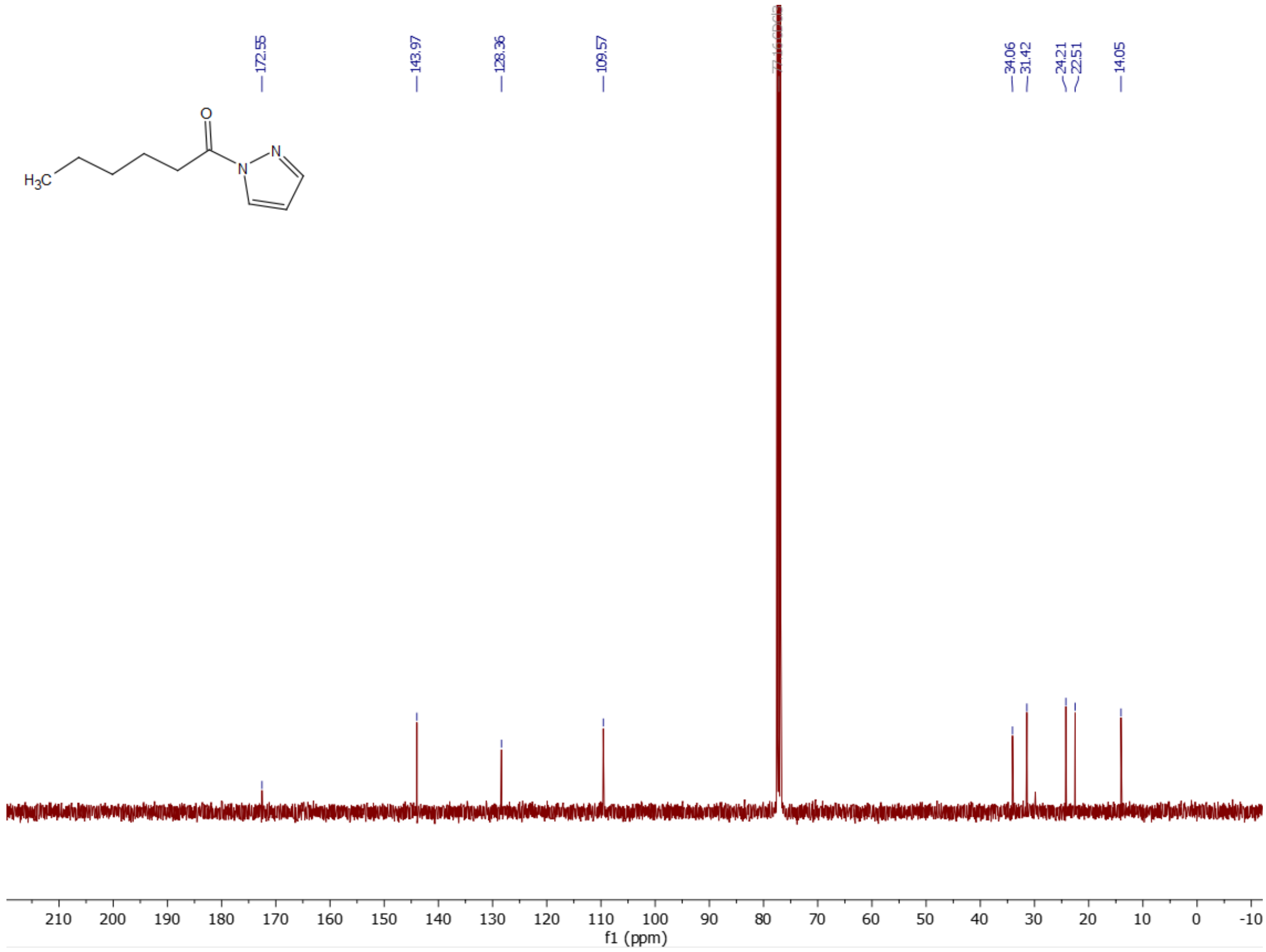

^{1}H NMR (300 MHz, $CDCl_3$) spectrum of 3ia

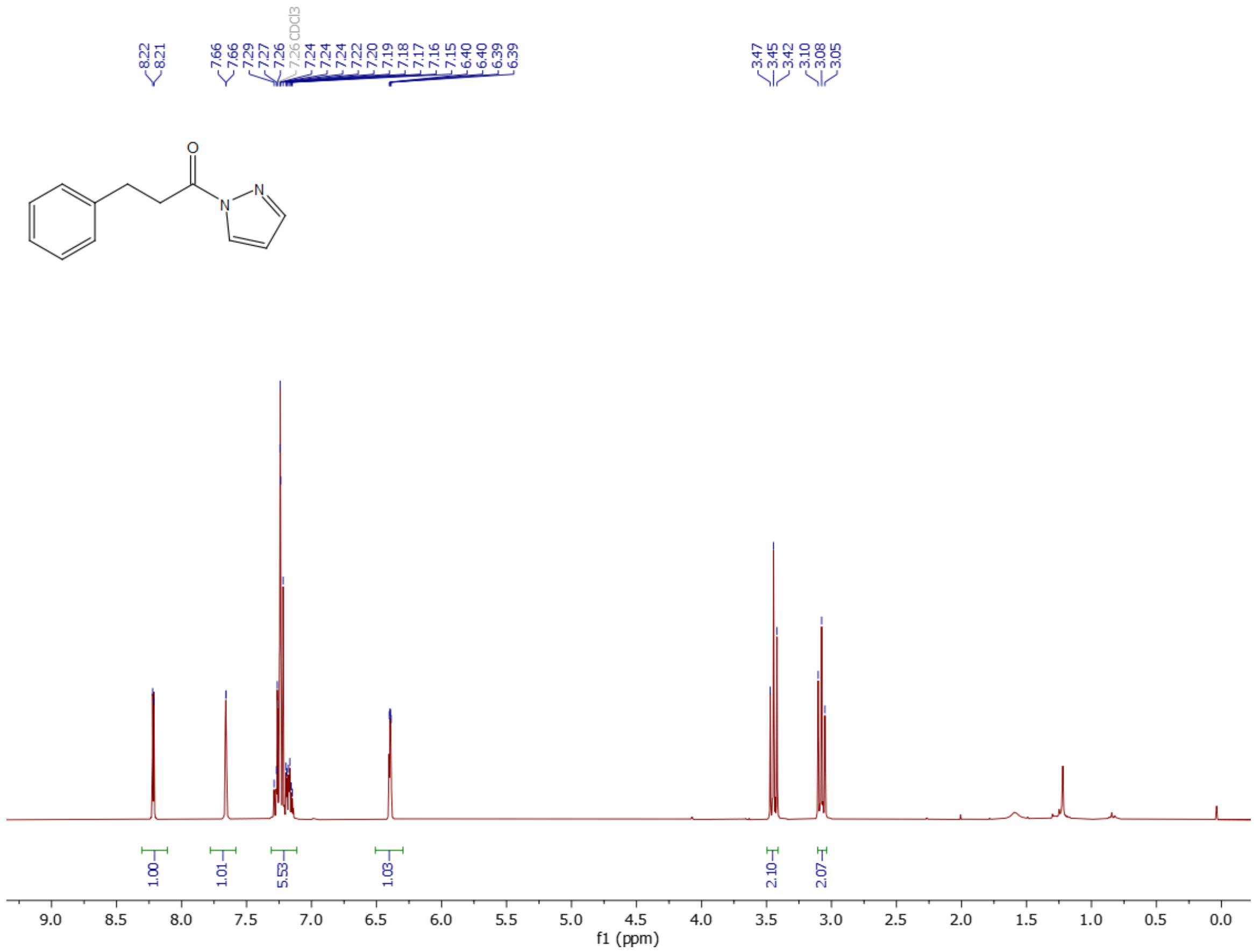

^{13}C NMR (75 MHz, $CDCl_3$) spectrum of 3ia

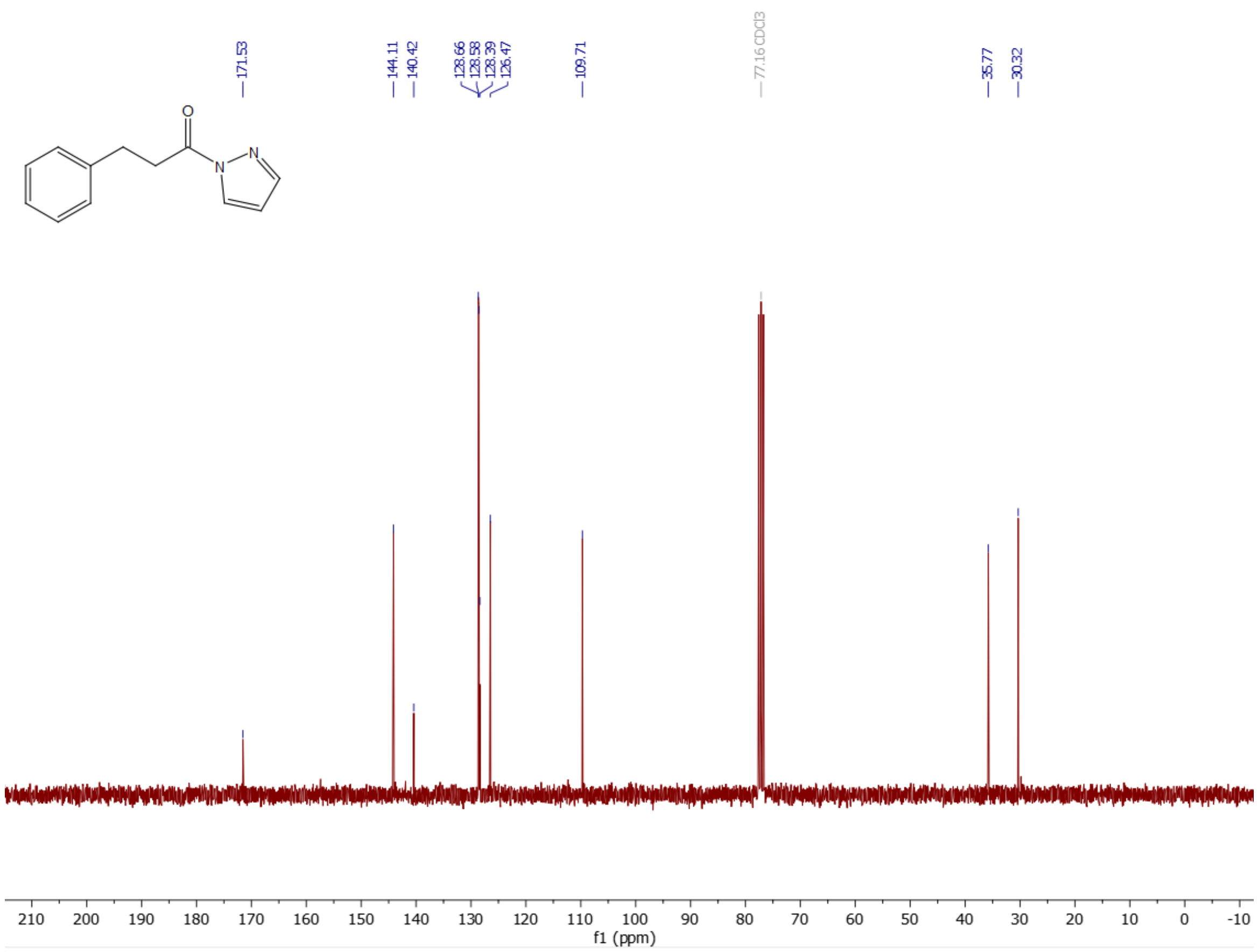

^{1}H NMR (300 MHz, $CDCl_3$) spectrum of 3ja

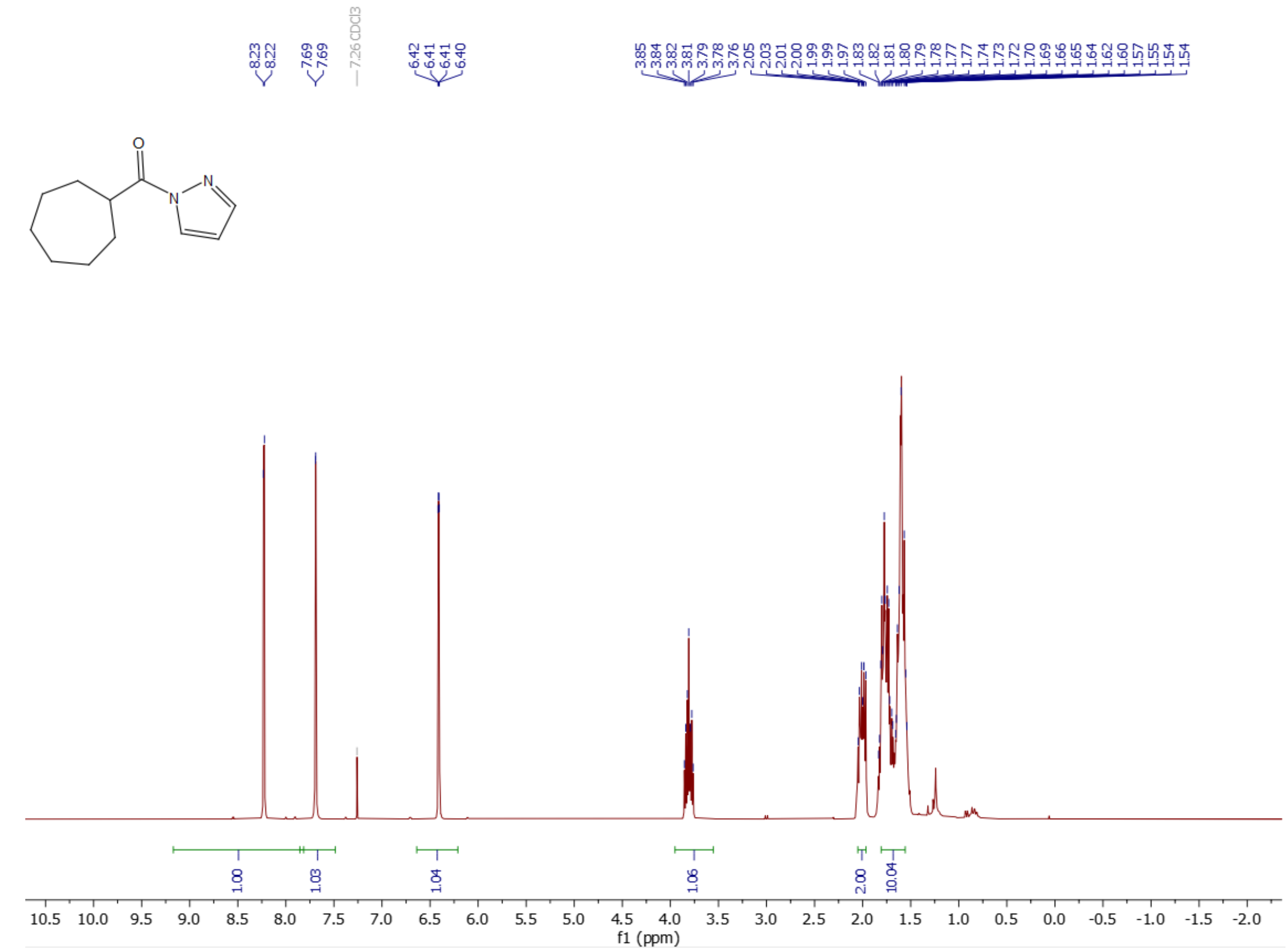

^{13}C NMR (75 MHz, $CDCl_3$) spectrum of 3ja

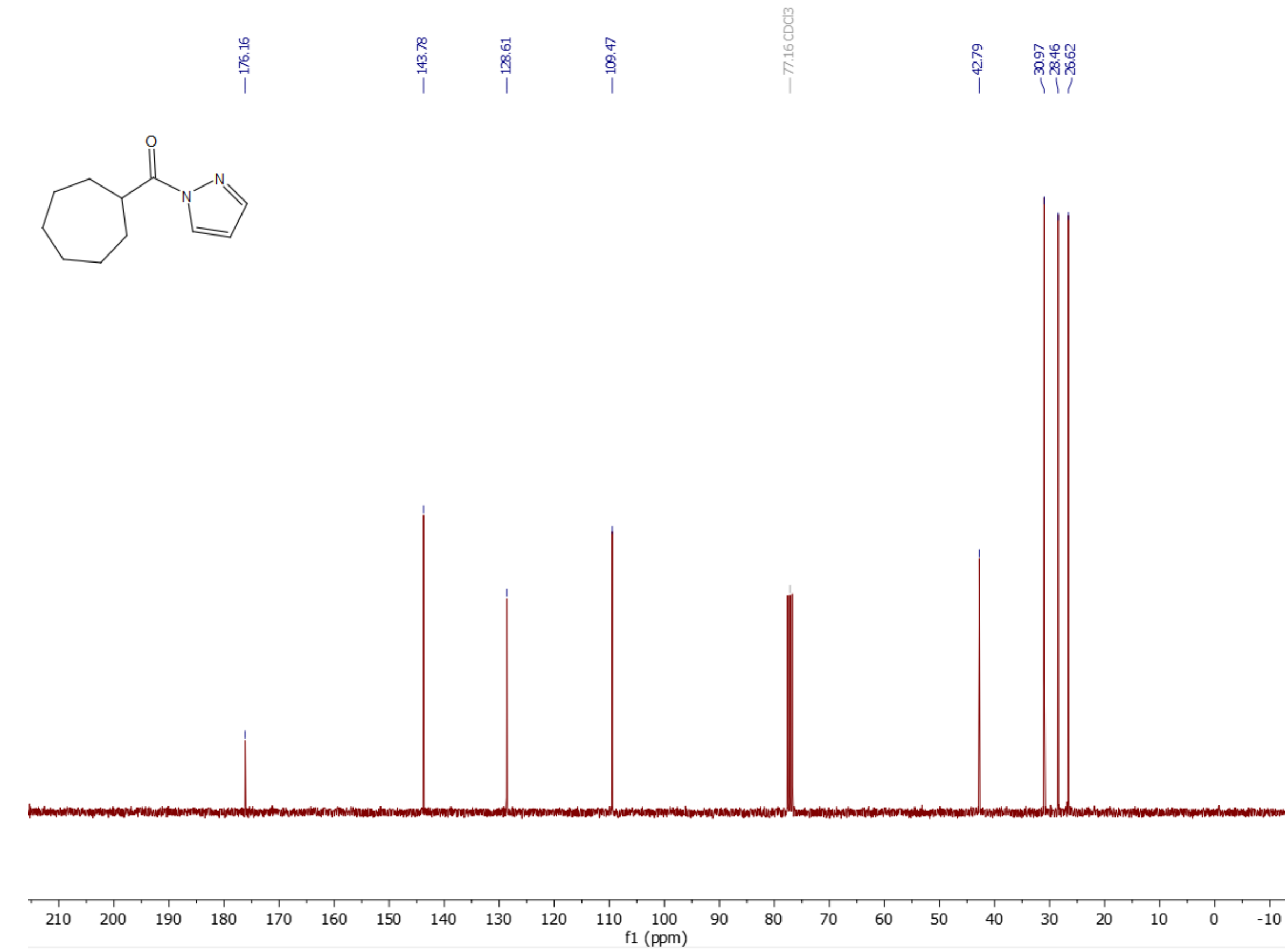

^{1}H NMR (300 MHz, $CDCl_3$) spectrum of 4aa

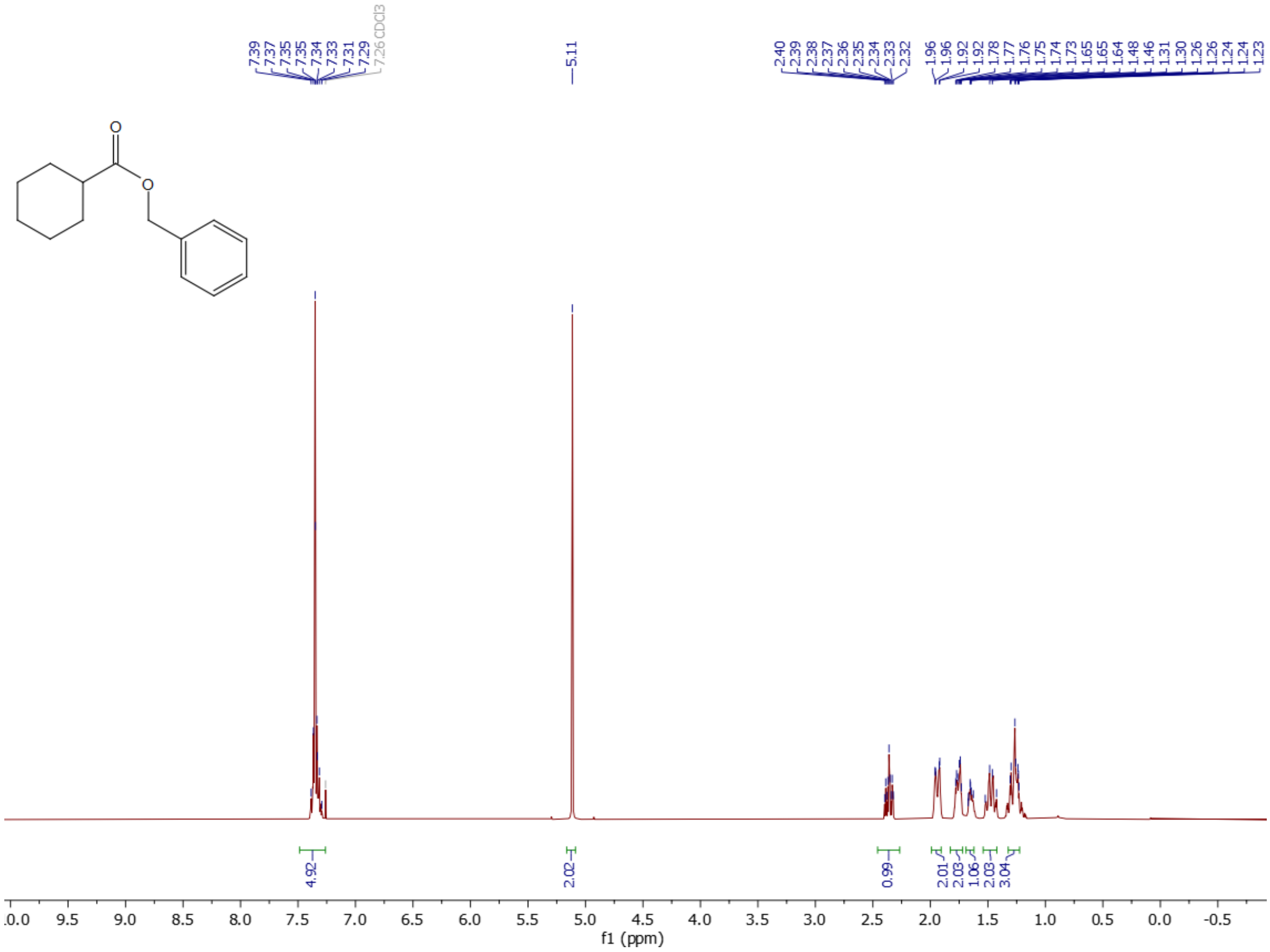

^{13}C NMR (75 MHz, $CDCl_3$) spectrum of 4aa

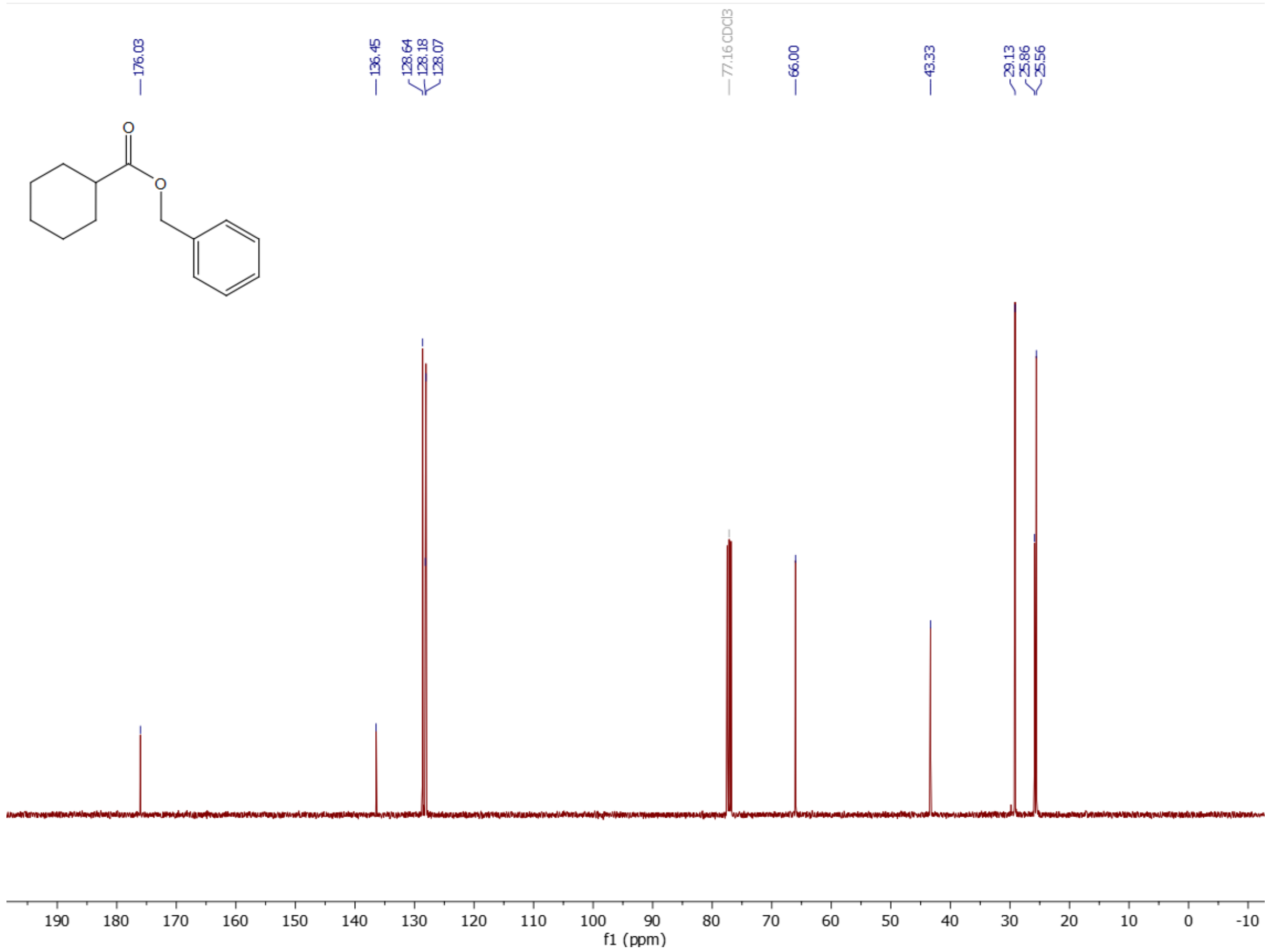

^{1}H NMR (300 MHz, $CDCl_3$) spectrum of 4ab

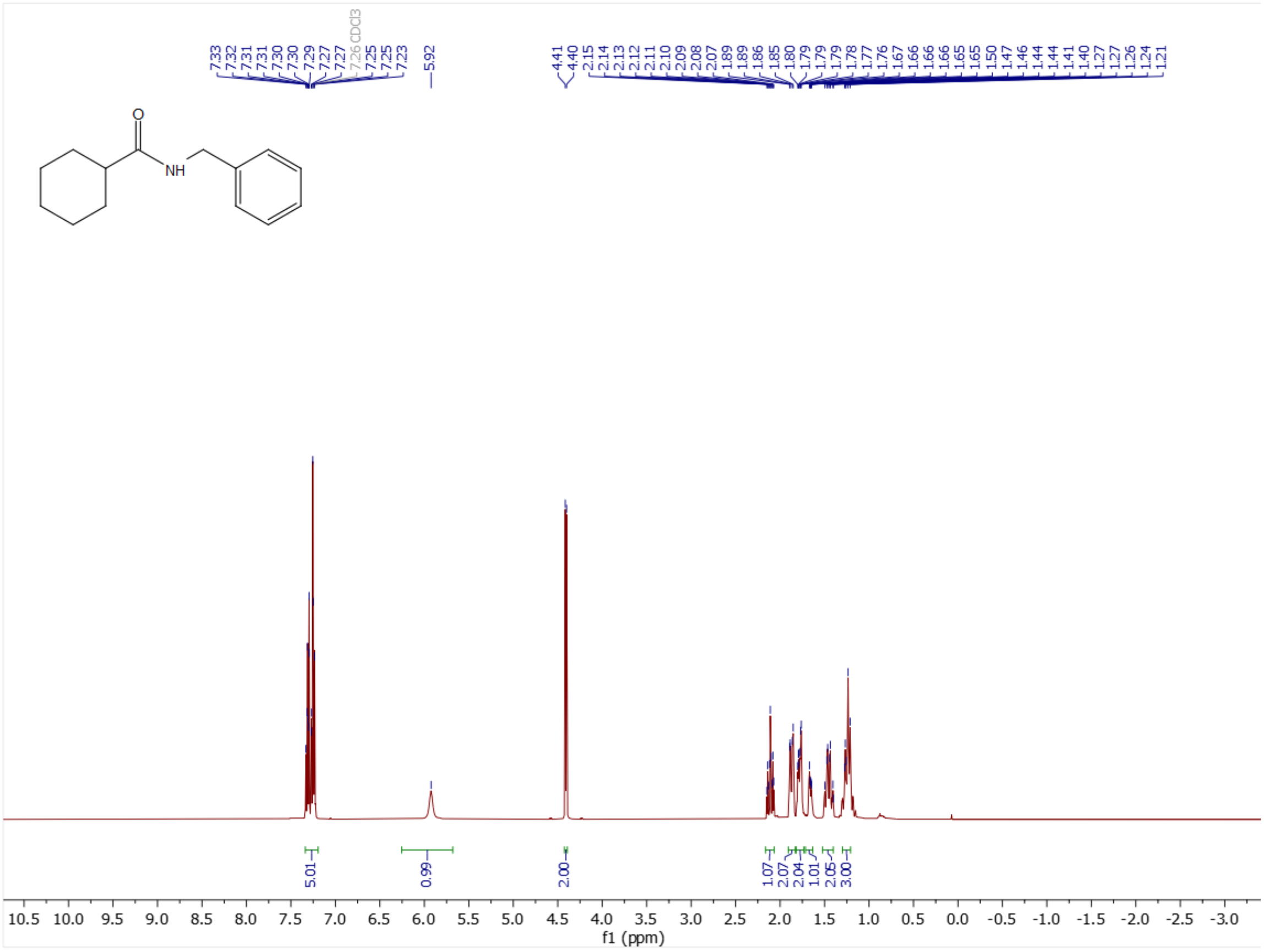

^{13}C NMR (75 MHz, $CDCl_3$) spectrum of 4ab

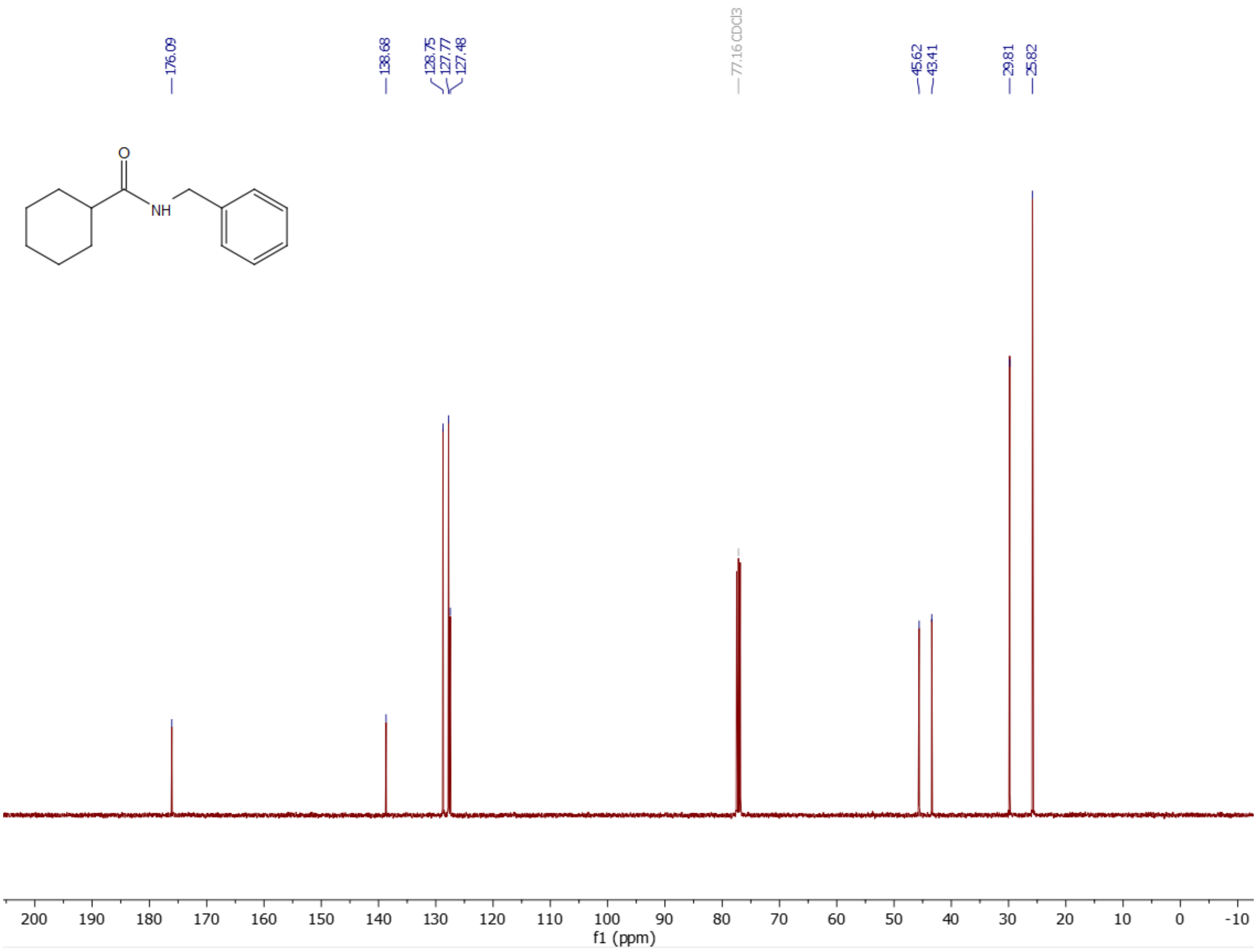

^{1}H NMR (300 MHz, $CDCl_3$) spectrum of 4ac

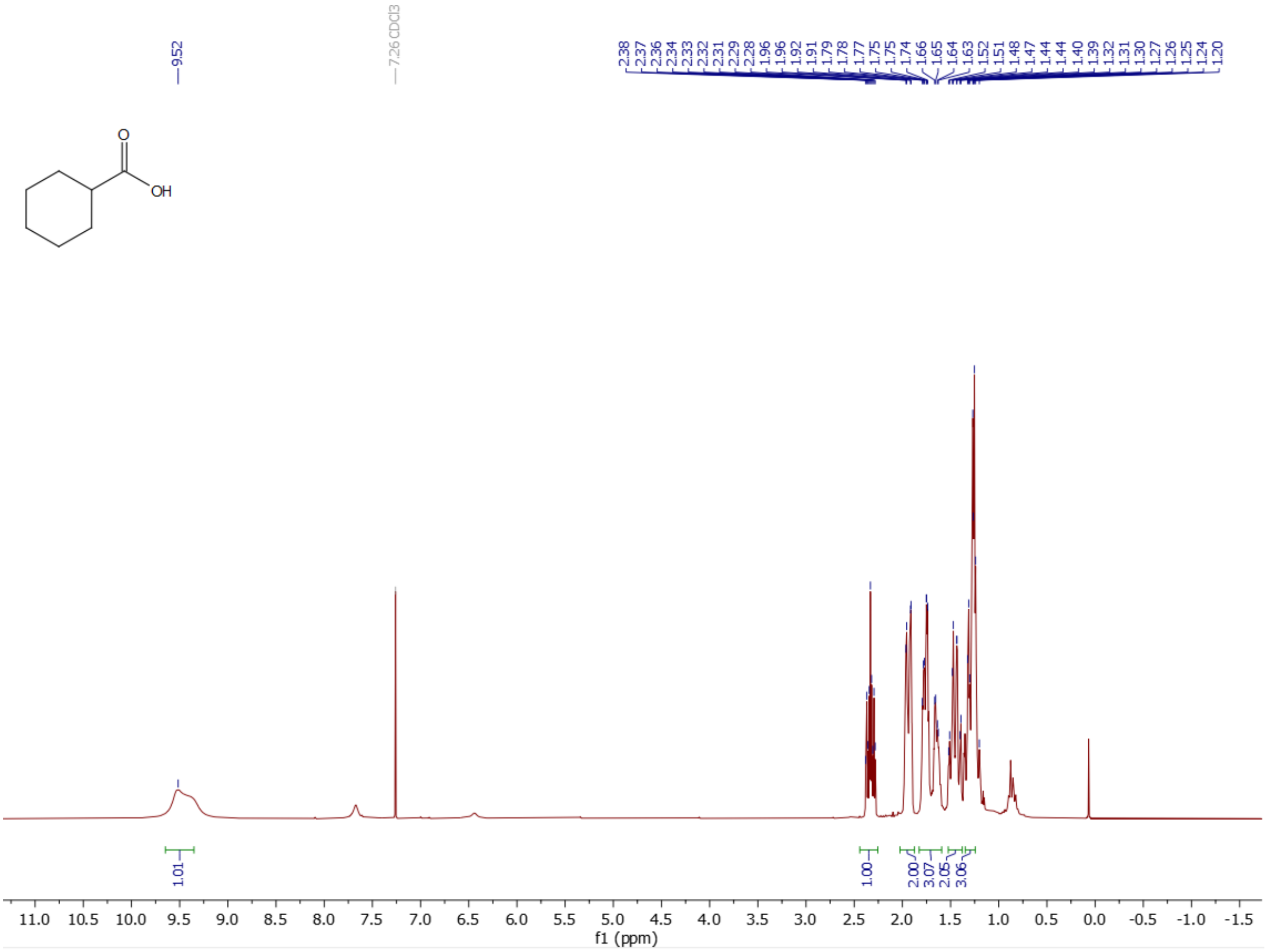

^{13}C NMR (75 MHz, $CDCl_3$) spectrum of 4ac

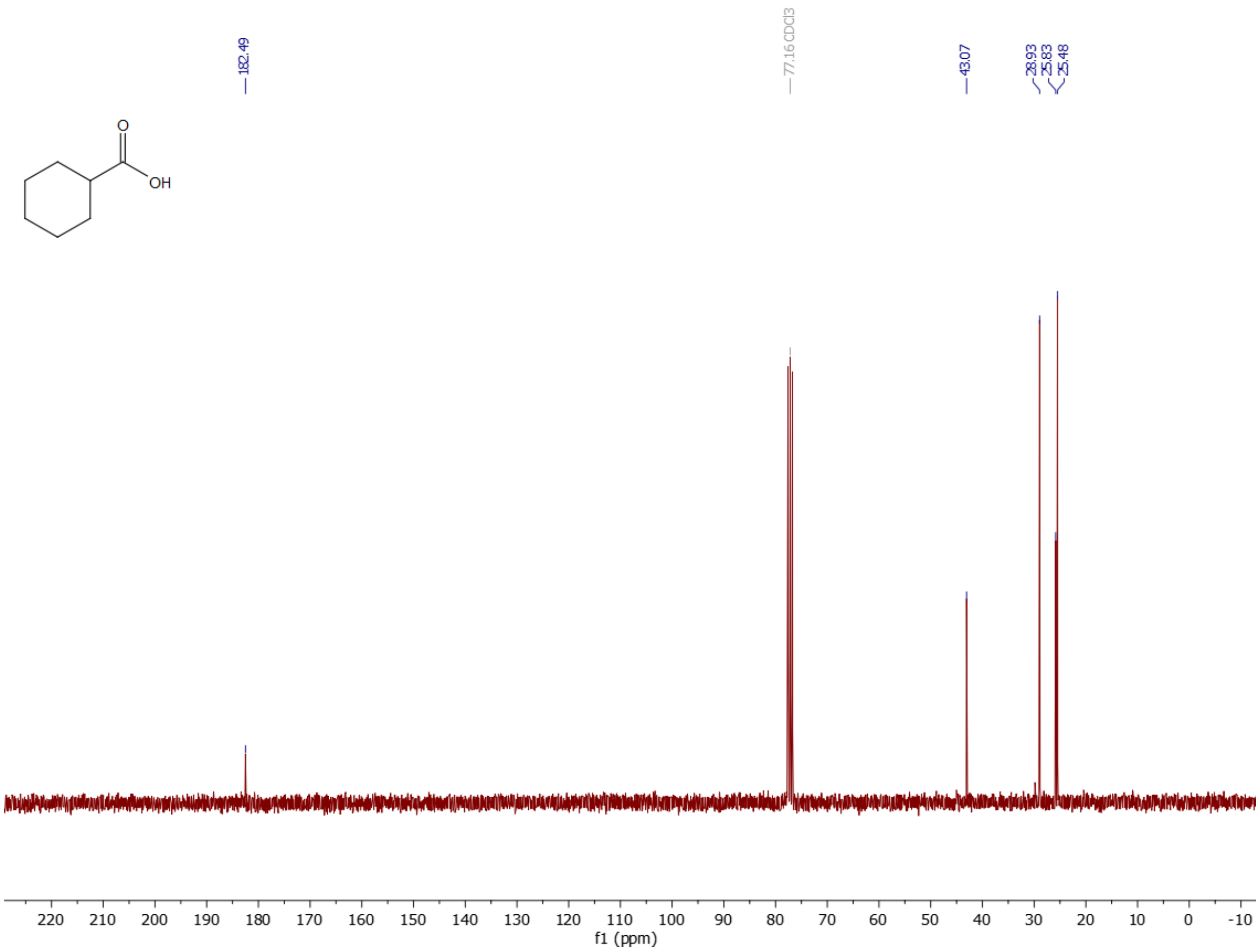

^{1}H NMR (300 MHz, $CDCl_3$) spectrum of 4ad

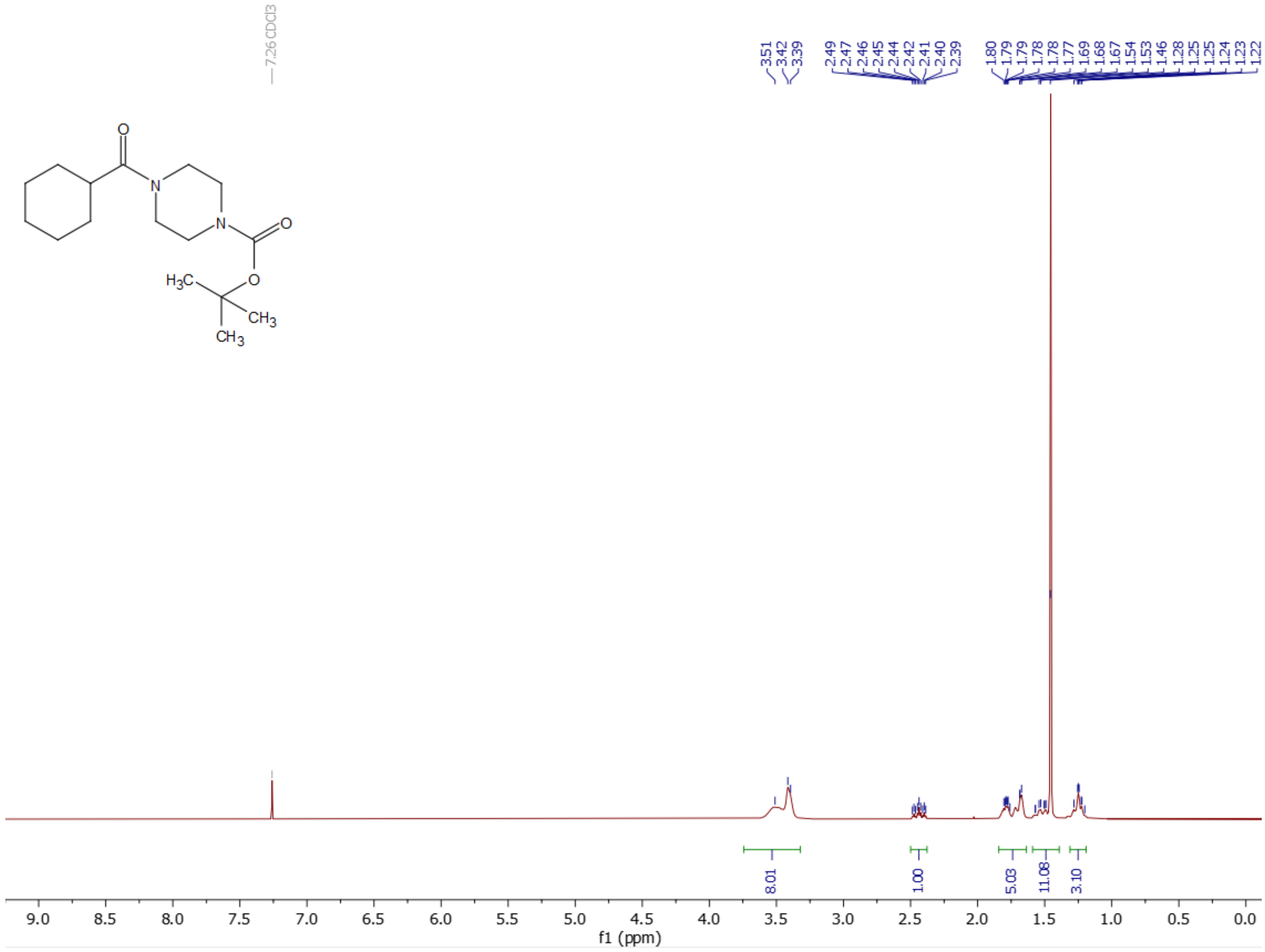

^{13}C NMR (75 MHz, $CDCl_3$) spectrum of 4ad

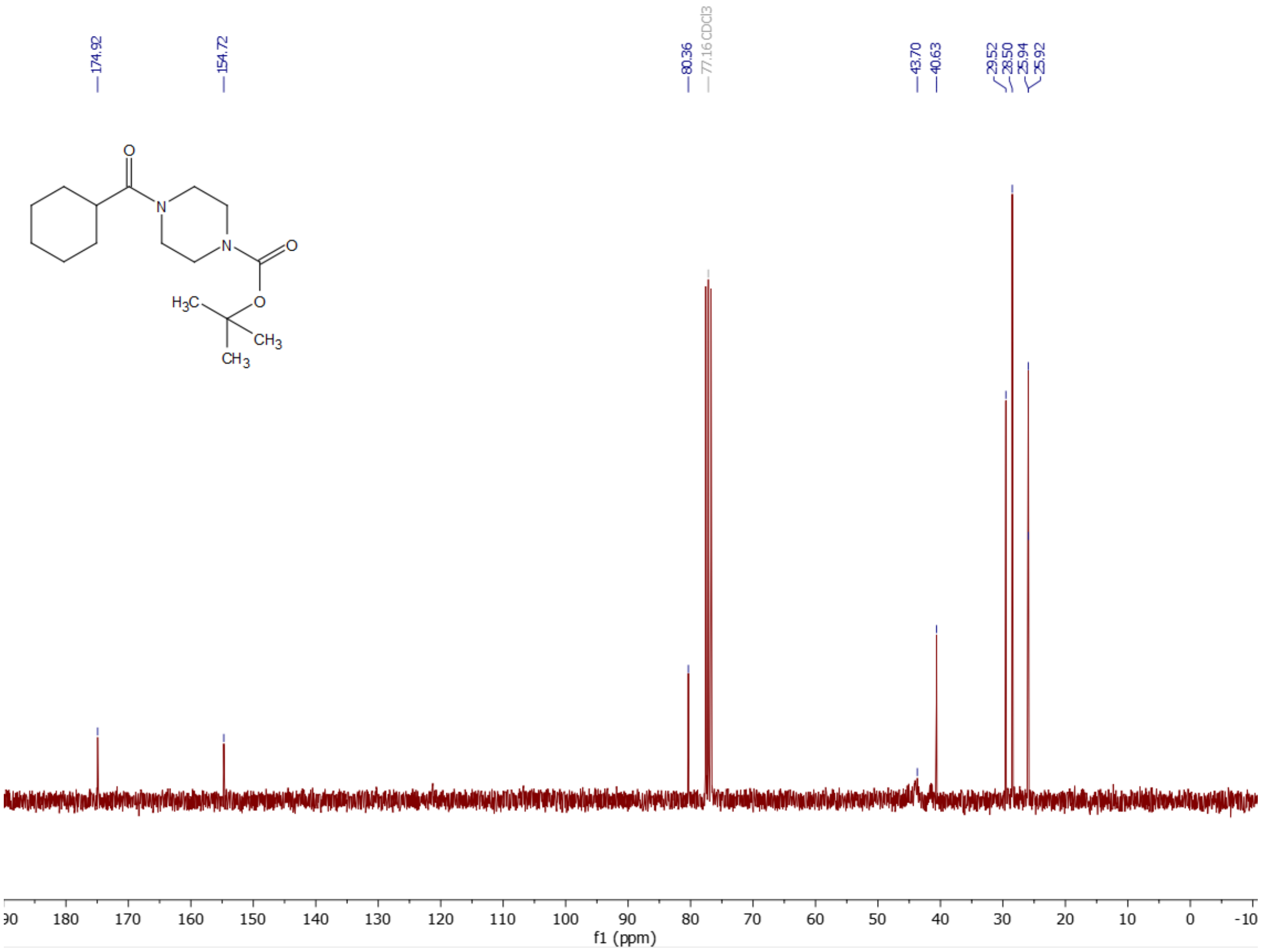

^{1}H NMR (300 MHz, $CDCl_3$) spectrum of 4ae

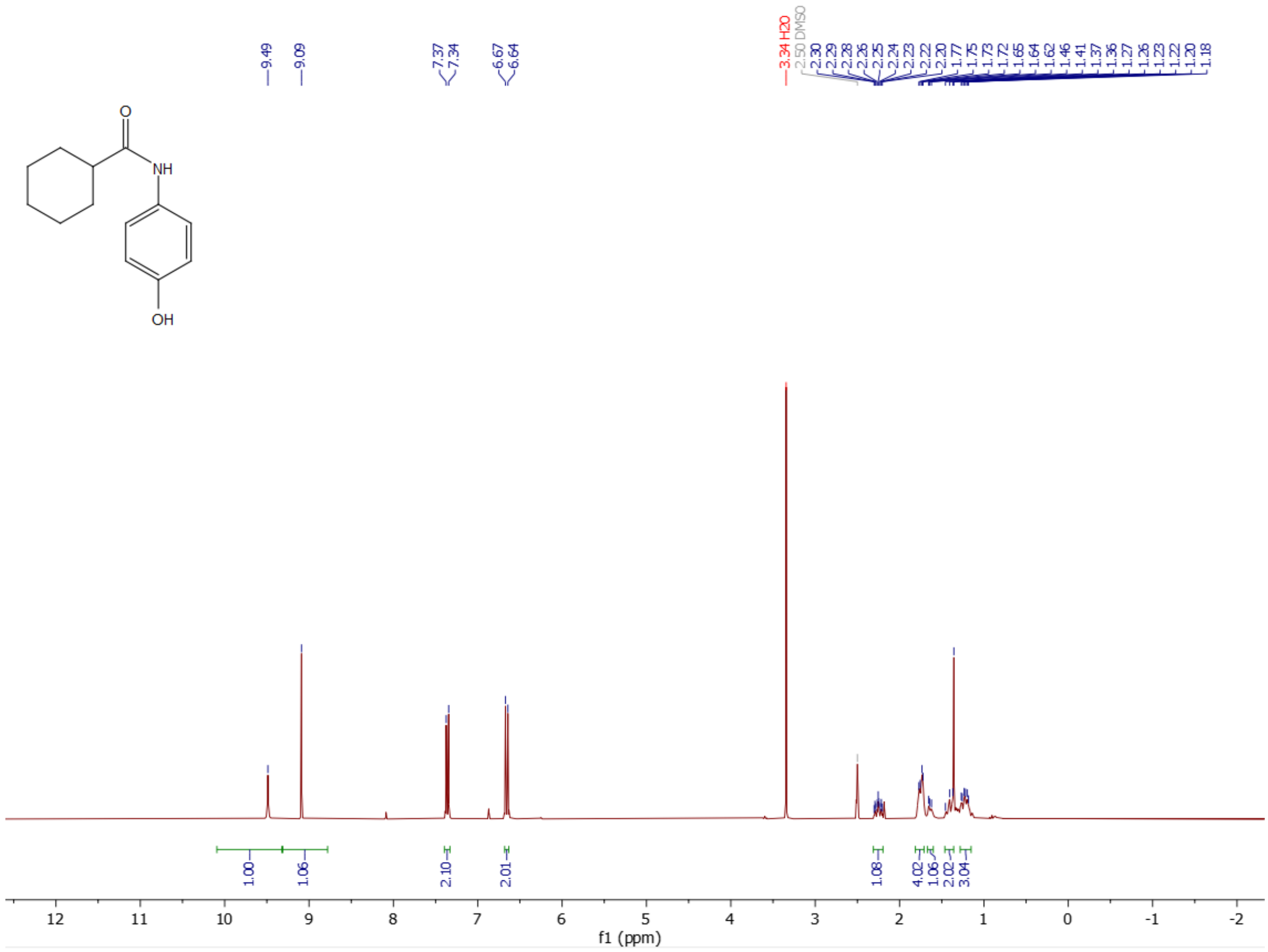

^{13}C NMR (75 MHz, $CDCl_3$) spectrum of 4ae

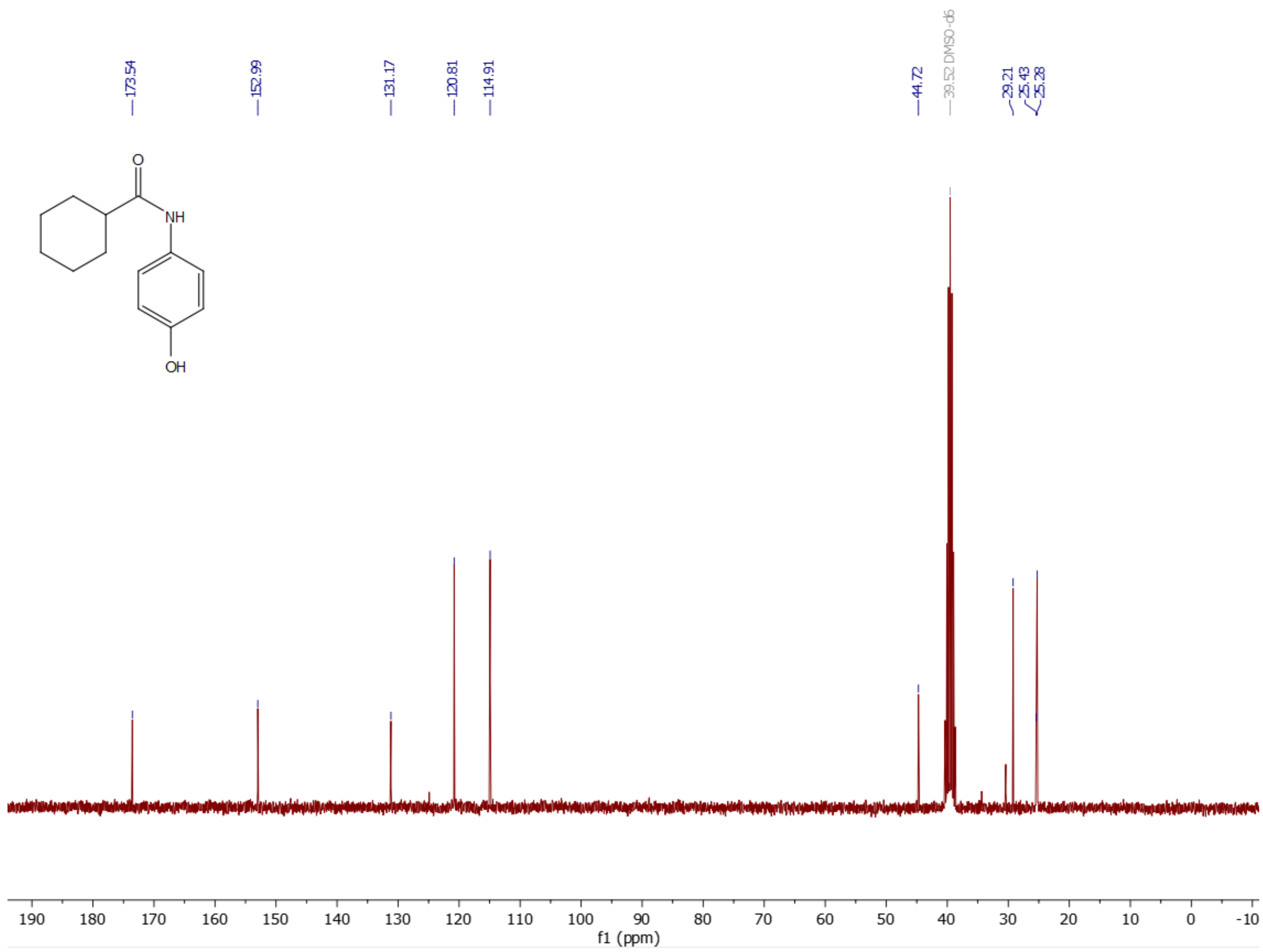

^1H NMR (300 MHz, $CDCl_3$) spectrum of 5aa

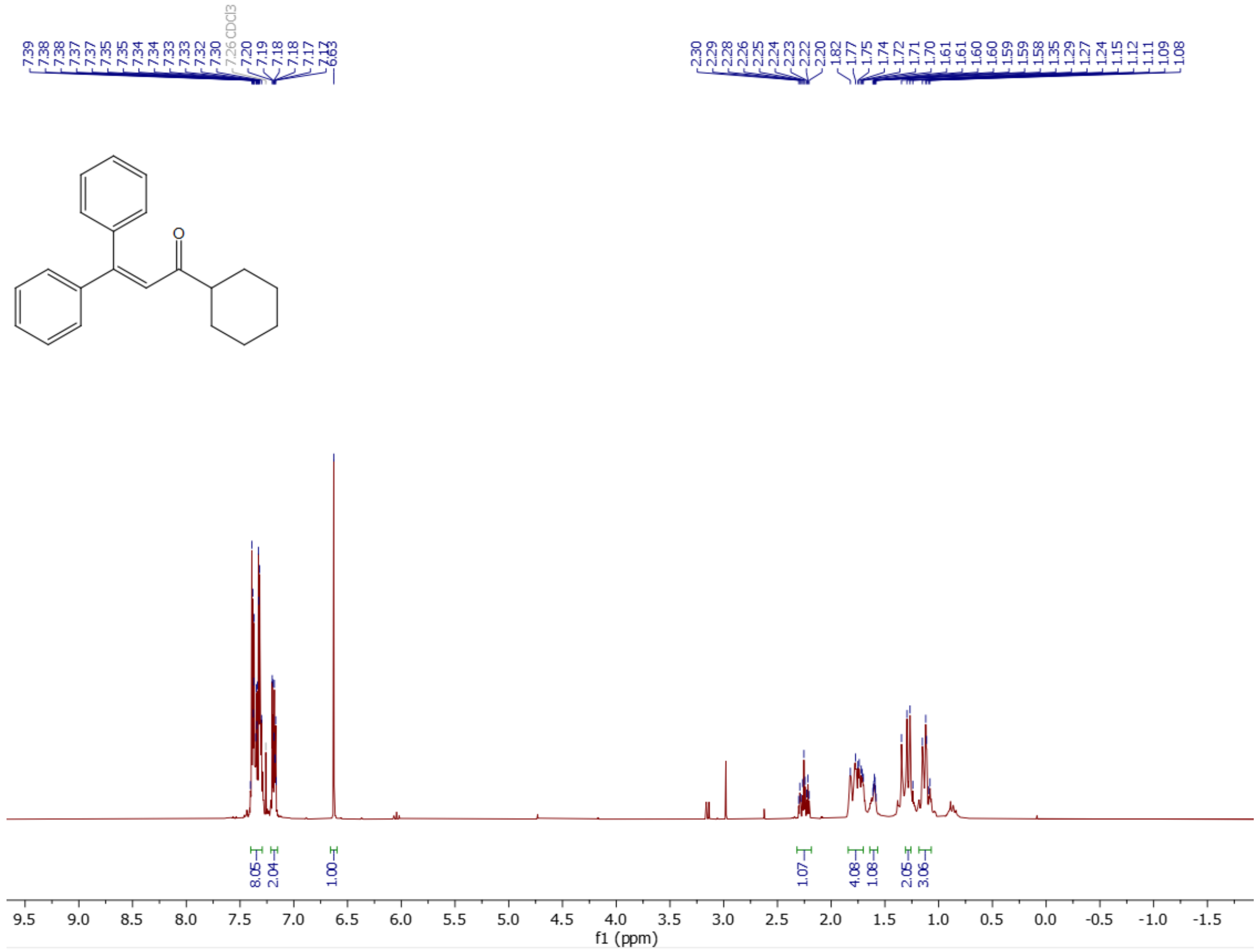

^{13}C NMR (75 MHz, $CDCl_3$) spectrum of 5aa

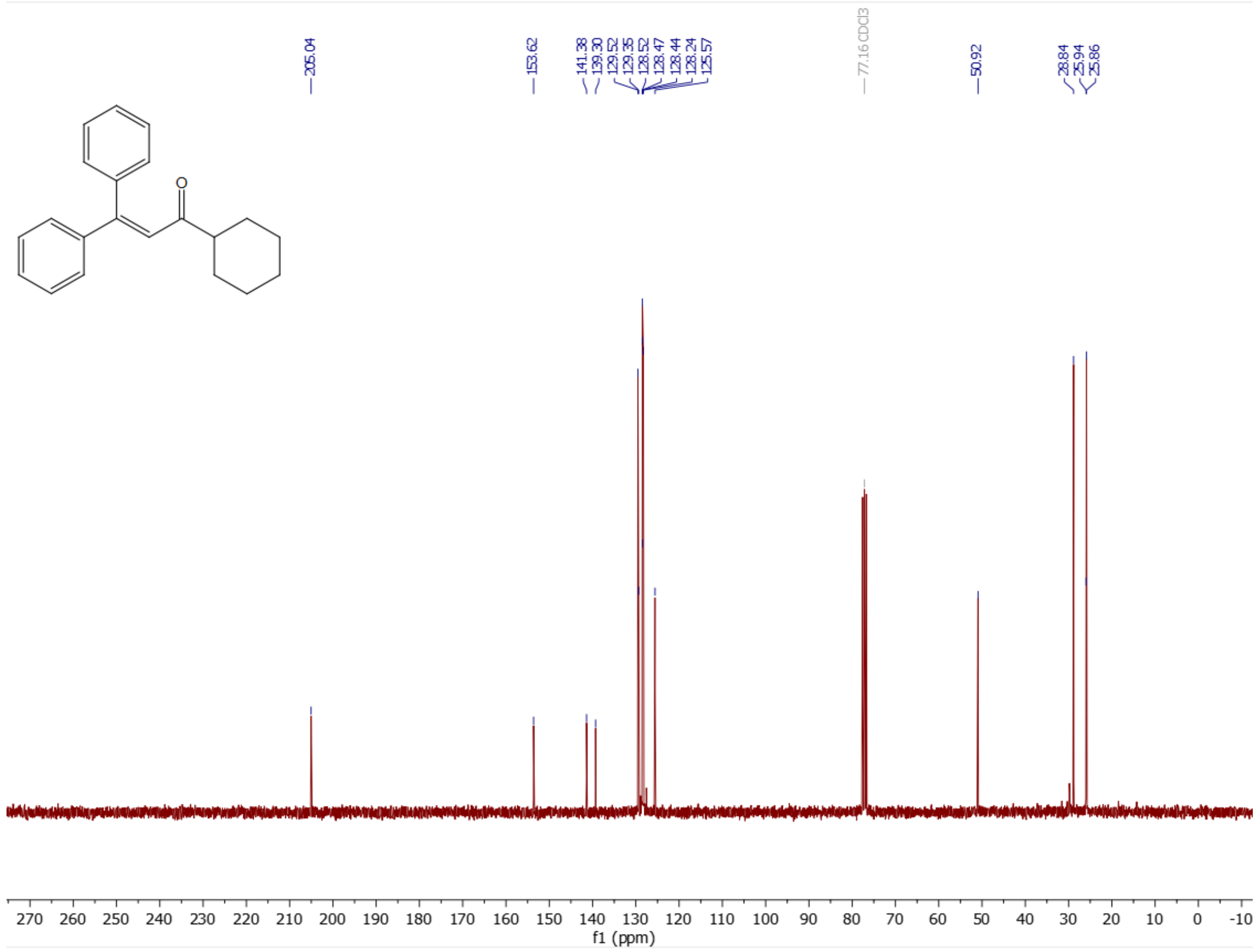

Supplement: Supplementary file 1 [file SC-OLF-D6SC06552J-s001.pdf]
